# Transcriptome and proteome profiling reveals complex adaptations of *Candida parapsilosis* cells assimilating hydroxyaromatic carbon sources

**DOI:** 10.1371/journal.pgen.1009815

**Published:** 2022-03-07

**Authors:** Andrea Cillingová, Renáta Tóth, Anna Mojáková, Igor Zeman, Romana Vrzoňová, Barbara Siváková, Peter Baráth, Martina Neboháčová, Zuzana Klepcová, Filip Brázdovič, Hana Lichancová, Viktória Hodorová, Broňa Brejová, Tomáš Vinař, Sofia Mutalová, Veronika Vozáriková, Giacomo Mutti, Ľubomír Tomáška, Atilla Gácser, Toni Gabaldón, Jozef Nosek

**Affiliations:** 1 Department of Biochemistry, Faculty of Natural Sciences, Comenius University in Bratislava, Bratislava, Slovakia; 2 HCEMM-USZ Department of Microbiology, University of Szeged, Szeged, Hungary; 3 MTA-SZTE Lendület Mycobiome Research Group, University of Szeged, Szeged, Hungary; 4 Institute of Chemistry, Slovak Academy of Sciences, Bratislava, Slovakia; 5 Department of Computer Science, Faculty of Mathematics, Physics and Informatics, Comenius University in Bratislava, Bratislava, Slovakia; 6 Department of Applied Informatics, Faculty of Mathematics, Physics and Informatics, Comenius University in Bratislava, Bratislava, Slovakia; 7 Department of Genetics, Faculty of Natural Sciences, Comenius University in Bratislava, Bratislava, Slovakia; 8 Institute for Research in Biomedicine (IRB), The Barcelona Institute of Science and Technology, Barcelona, Spain; 9 Barcelona Supercomputing Centre (BSC-CNS), Barcelona, Spain; 10 Catalan Institution for Research and Advanced Studies (ICREA), Barcelona, Spain; 11 Centro de Investigación Biomédica En Red de Enfermedades Infecciosas (CIBERINFEC), Barcelona, Spain; The University of North Carolina at Chapel Hill, UNITED STATES

## Abstract

Many fungal species utilize hydroxyderivatives of benzene and benzoic acid as carbon sources. The yeast *Candida parapsilosis* metabolizes these compounds via the 3-oxoadipate and gentisate pathways, whose components are encoded by two metabolic gene clusters. In this study, we determine the chromosome level assembly of the *C*. *parapsilosis* strain CLIB214 and use it for transcriptomic and proteomic investigation of cells cultivated on hydroxyaromatic substrates. We demonstrate that the genes coding for enzymes and plasma membrane transporters involved in the 3-oxoadipate and gentisate pathways are highly upregulated and their expression is controlled in a substrate-specific manner. However, regulatory proteins involved in this process are not known. Using the knockout mutants, we show that putative transcriptional factors encoded by the genes *OTF1* and *GTF1* located within these gene clusters function as transcriptional activators of the 3-oxoadipate and gentisate pathway, respectively. We also show that the activation of both pathways is accompanied by upregulation of genes for the enzymes involved in β-oxidation of fatty acids, glyoxylate cycle, amino acid metabolism, and peroxisome biogenesis. Transcriptome and proteome profiles of the cells grown on 4-hydroxybenzoate and 3-hydroxybenzoate, which are metabolized via the 3-oxoadipate and gentisate pathway, respectively, reflect their different connection to central metabolism. Yet we find that the expression profiles differ also in the cells assimilating 4-hydroxybenzoate and hydroquinone, which are both metabolized in the same pathway. This finding is consistent with the phenotype of the Otf1p-lacking mutant, which exhibits impaired growth on hydroxybenzoates, but still utilizes hydroxybenzenes, thus indicating that additional, yet unidentified transcription factor could be involved in the 3-oxoadipate pathway regulation. Moreover, we propose that bicarbonate ions resulting from decarboxylation of hydroxybenzoates also contribute to differences in the cell responses to hydroxybenzoates and hydroxybenzenes. Finally, our phylogenetic analysis highlights evolutionary paths leading to metabolic adaptations of yeast cells assimilating hydroxyaromatic substrates.

## Author summary

Benzene and its derivatives are simple aromatic compounds representing key substances for the chemical industry. While benzene itself is toxic and carcinogenic, benzoic acid is commonly used in the food industry and some of its derivatives are used in pharmacology (aspirin) or cosmetics (parabens). The benzene ring of aromatic molecules is relatively stable, but many microorganisms including yeasts break it enzymatically and, in a series of biochemical reactions, utilize resulting metabolites as carbon sources. Understanding the genetic basis of corresponding metabolic pathways and their regulation opens a venue for applications in biotechnology and bioremediation of polluted environments. Here we investigate the yeast *Candida parapsilosis* which assimilates various hydroxybenzenes and hydroxybenzoates via the 3-oxoadipate and gentisate pathways. We show that the genes coding for the substrate transporters and enzymes involved in both pathways are co-expressed and regulated by the transcriptional activators Otf1p and Gtf1p, respectively. Our results also reveal the connections of both pathways to central metabolism and organelle biogenesis and provide an insight into evolution of metabolism of hydroxyaromatic compounds.

## Introduction

Metabolic gene clusters (MGCs) are composed of co-localized genes, whose products participate in the same metabolic pathway. In most cases, their functions are linked to the production of secondary metabolites or the assimilation of unconventional substrates. Such biochemical pathways are usually nonessential, but in specific circumstances they may provide a growth benefit for the host organism. In general, MGCs encode the enzymes catalyzing reactions in a biochemical pathway, membrane transporters for substrates or metabolites, as well as transcription factors that control the expression of corresponding genes. Gene clustering thus generates functional genetic modules whose co-regulated expression facilitates rapid adaptation of cellular metabolism to environmental changes [1,2]. The occurrence of MGCs in eukaryotic genomes was originally considered to be rare. However, bioinformatic analyses of a constantly increasing number of sequenced genomes show that the gene clusters are their typical feature, especially in case of fungal and plant genomes [3–5]. The formation of MGCs also facilitates their transmission via horizontal gene transfer, thus contributing to metabolic diversity of fungal species and their ecological adaptation [6]. Investigations of MGCs provide a venue for elucidating their evolutionary origin, genetic organization, and expression, as well as the coordination of the corresponding biochemical pathways with the central cellular metabolism.

Previously, we identified and characterized several genes from the pathogenic yeast *Candida parapsilosis* arranged in two MGCs, which are conserved in the genomes of yeast species from the ‘CUG-Ser1’ clade of the subphylum Saccharomycotina [7–12]. These MGCs code for enzymes of the gentisate (GP) and 3-oxoadipate (3-OAP) pathways that are involved in catabolic degradation of a broad spectrum of hydroxyderivatives of benzene and benzoic acid. While 3-hydroxybenzoate and gentisate (2,5-dihydroxybenzoate) are metabolized via the GP, 4-hydroxybenzoate, 2,4-dihydroxybenzoate, protocatechuate (3,4-dihydroxybenzoate), hydroquinone, and resorcinol are degraded via the hydroxyhydroquinone (HHQ) branch of the 3-OAP [13,14]. The resulting products of both biochemical pathways (i.e. fumarate and pyruvate in the GP; succinate and acetyl-CoA in the 3-OAP) can be channeled into tricarboxylic acid (TCA) cycle operating in mitochondria. Interconnection of both pathways with these organelles is mediated by metabolite carriers in the inner mitochondrial membrane (i.e. Sfc1p, Leu5p, Yhm2p, and Mpc1p). Moreover, the enzymes catalyzing the last two steps of the 3-OAP (i.e. 3-oxoadipate:succinyl-CoA transferase (Osc1p) and 3-oxoadipyl-CoA thiolase (Oct1p)) are imported into mitochondria [9,11]. In addition, we have previously identified a family of genes coding for the plasma membrane transporters for hydroxybenzoates [10]. While both pathways are repressed in cells assimilating glucose, corresponding genes are highly induced during cultivation on media containing a hydroxyaromatic substrate as a sole carbon source [7,9–11]. Although the transcriptional factors involved in this regulation have not yet been identified, both MGCs contain a gene for uncharacterized zinc cluster transcription factor representing a candidate transcriptional activator of the corresponding pathway.

In this study, we investigate the regulation of the 3-OAP and GP as well as the coordination of both pathways with central metabolism and organelle biogenesis. Using the analysis of transcriptomic and proteomic profiles of *C*. *parapsilosis* cells assimilating hydroxyaromatic compounds we show that the induction of both pathways is accompanied by the upregulation of genes whose products are involved in β-oxidation of fatty acids (FA), glyoxylate cycle, metabolism of amino acids, and the biogenesis of peroxisomes. Our results also highlight the differences between the metabolism of hydroxybenzoates and hydroxybenzenes. Moreover, we demonstrate experimentally that putative transcription factors named Gtf1p and Otf1p function as transcriptional activators of the GP and 3-OAP genes, respectively. Their phylogenetic analysis shed additional insight into the evolution of both biochemical pathways.

## Results and discussion

### Gene expression landscape of *C*. *parapsilosis* cells assimilating hydroxyaromatic carbon sources

**Fig 1:**
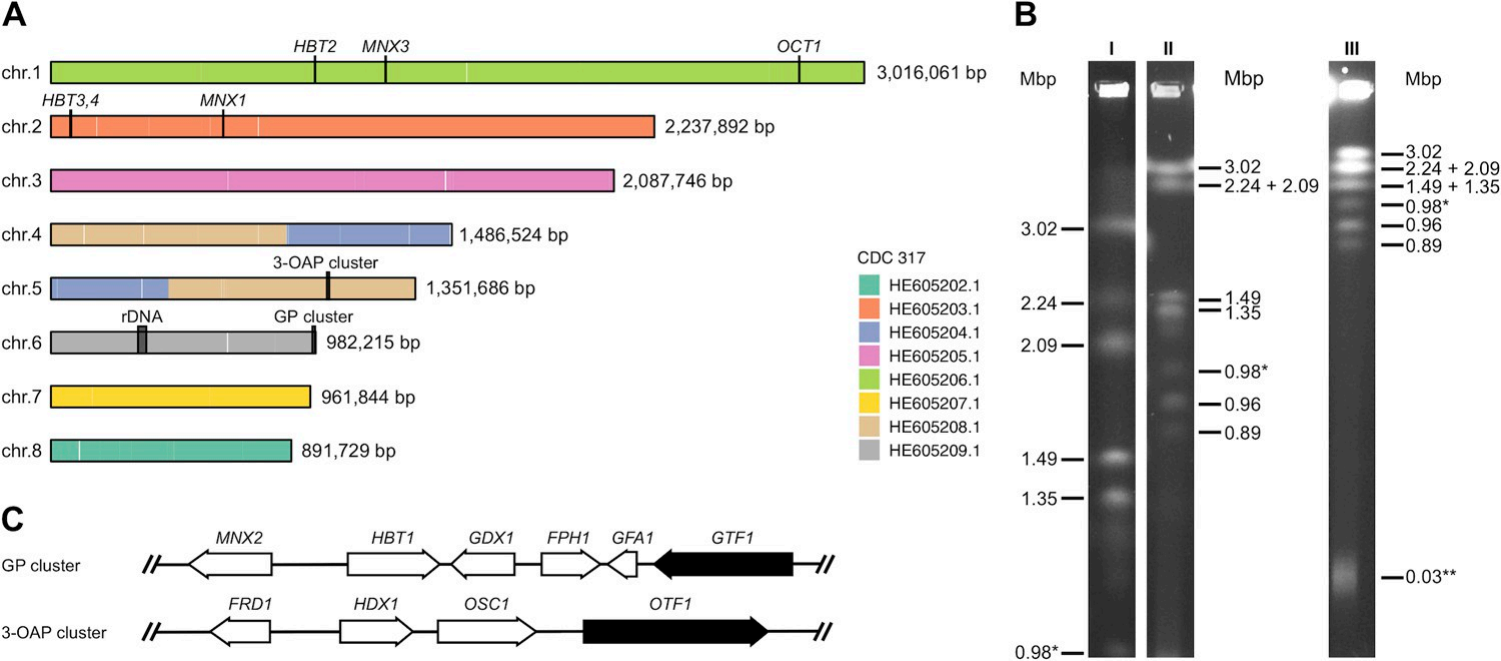
Nuclear genome organization of the *C*. *parapsilosis* strain CLIB214. (A) Chromosomal contigs of *C*. *parapsilosis* CLIB214. The colouring is based on alignments with the nuclear contigs of the reference genome sequence (CDC317) (see Materials and Methods for details). (B) Electrophoretic karyotype of CLIB214. DNA samples prepared in agarose blocks were separated by PFGE at three different conditions (I, II, and III) as described in Materials and Methods. The bands corresponding to the chromosome containing an rDNA array (0.98 Mbp) and the linear mitochondrial DNA (32.8 kbp) are indicated by one and two asterisks, respectively. (C) Organization of the GP and 3-OAP gene clusters. The genes coding for the transcription activators Gtf1p and Otf1p investigated in this study are shown in black.

Several studies have demonstrated that *C*. *parapsilosis* assimilates a broad spectrum of hydroxyderivatives of benzene and benzoic acid via the GP and 3-OAP [7,13]. To investigate the regulation of both pathways we analyzed *C*. *parapsilosis* cells grown in media containing hydroxyaromatic compounds degraded either via the 3-OAP or GP. The gene expression analysis was performed in the strain CLIB214 (CBS604), which is, together with derived mutants, commonly used in experimental studies [e.g. 15–18]. This strain was originally isolated from a patient with tropical diarrhea in Puerto Rico [19] and it represents the type strain of *C*. *parapsilosis*. Although a genome sequence survey of CLIB214 was carried out in 2005 by Sanger sequencing [20], the complete genome sequence of this strain was not available. Here, we determined the chromosome level genome assembly of the CLIB214 strain by combining Oxford Nanopore and Illumina sequencing technologies and used it for analyses of cells utilizing hydroxyaromatic substrates (see below). The resulting CLIB214 assembly has a total length of 13.0 Mbp and consists of 8 nuclear chromosomes corresponding to the electrophoretic karyotype determined by PFGE (Fig 1). Alignments with the reference genome sequence of the strain CDC317 [21] cover 99.5% of the assembly and have a 99.9% identity. Compared to the CDC317 assembly, there is a single large-scale translocation between chromosomes 4 and 5 (CDC317 contigs HE605208.1 and HE605204.1). Annotation of the nuclear chromosomes contains 5,856 predicted protein-coding genes; 5,797 of them overlap with protein coding genes mapped from the CDC317 strain. The components of 3-OAP are encoded by a cluster comprising four genes (i.e. *FRD1*, *HDX1*, *OSC1*, *OTF1*) located on chromosome 5, as well as by several additional loci on chromosomes 1 and 2. The six genes coding for the GP components (i.e. *MNX2*, *HBT1*, *GDX1*, *FPH1*, *GFA1*, *GTF1*) are localized in a single MGC which is located in the subtelomeric region of chromosome 6 (Fig 1**)**. The GP gene cluster is similar to the MGC encoding stilbene dioxygenase which is present in Pezizomycotina species [22,23], although it lacks a gene for this enzyme and instead of salicylate hydroxylase it codes for 3-hydroxybenzoate 6-hydroxylase [7].

**Fig 2:**
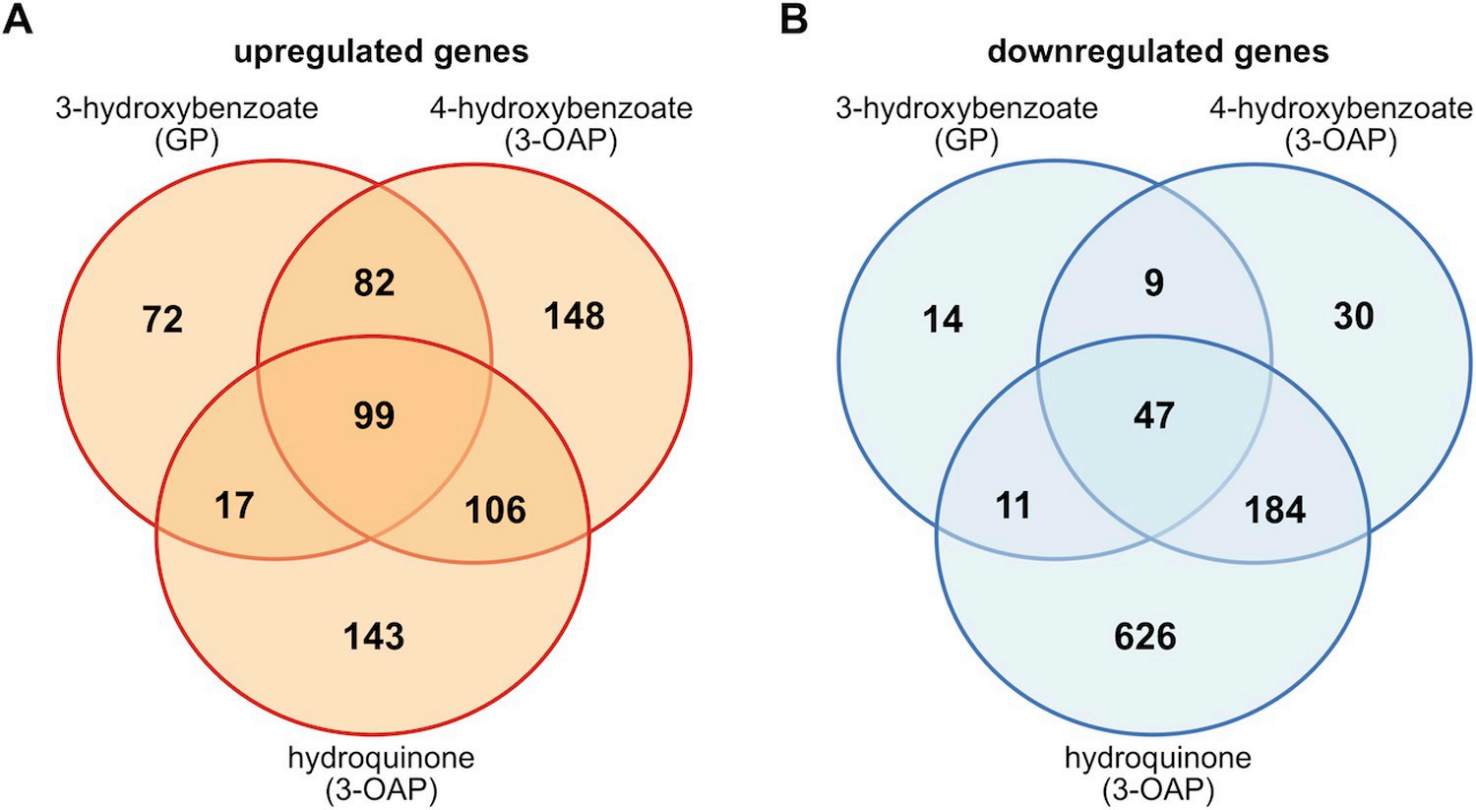
Differentially expressed genes identified by RNA-Seq analysis. The Venn diagrams show numbers of upregulated (log_2_ fold change ≥ 2; adjusted p-value ≤ 0.05; (A)) or downregulated (log_2_ fold change ≤ -2; adjusted p-value ≤ 0.05; (B)) genes in CLIB214 cells assimilating 3-hydroxybenzoate, 4-hydroxybenzoate or hydroquinone compared to galactose. The results are based on the lists of differentially expressed genes (S1 Table). The diagrams were drawn with a web tool (http://bioinformatics.psb.ugent.be/webtools/Venn/).

Next, we used the CLIB214 genome assembly as a reference for transcriptomic and proteomic experiments to investigate the activation of genes involved in the 3-OAP and GP and their links to central cellular metabolism and organelle biogenesis. In these experiments, we compared CLIB214 cells assimilating 4-hydroxybenzoate, hydroquinone (both metabolized via the 3-OAP) and 3-hydroxybenzoate (metabolized via the GP), with those utilizing galactose as a control carbon source. By RNA-Seq analysis, we identified 270, 435, and 365 genes upregulated four-fold or more in cells cultivated in media containing 3-hydroxybenzoate, 4-hydroxybenzoate, and hydroquinone, respectively, compared to control cells grown on galactose (Fig 2 and S1 Table).

**Fig 3:**
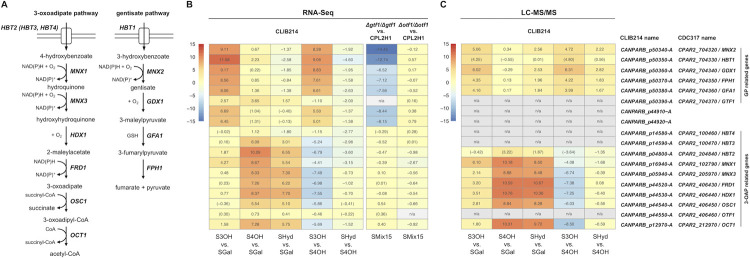
The 3-OAP and GP are induced in *C*. *parapsilosis* cells assimilating hydroxyaromatic compounds. (A) The simplified schemes depicting the enzymes and hydroxybenzoate transporters involved in the 3-OAP and GP in *C*. *parapsilosis*. (B) Differential expression of selected *C*. *parapsilosis* genes involved in the metabolism of hydroxyaromatic compounds. The expression was analyzed in CLIB214 cells grown to OD_600_ ~ 1 in synthetic media containing 3-hydroxybenzoate, 4-hydroxybenzoate or hydroquinone as a sole carbon source compared to the cells cultivated in medium with galactose (i.e. S3OH vs. SGal, S4OH vs. SGal, SHyd vs. SGal) or 4-hydroxybenzoate (S3OH vs. S4OH, SHyd vs. S4OH). Analysis of the mutants *Δgtf1/Δgtf1* and *Δotf1/Δotf1* is based on the comparison to the parental strain CPL2H1 (*Δgtf1/Δgtf1* vs. CPL2H1 and *Δotf1/Δotf1* vs. CPL2H1) grown in an SMix15 medium containing three hydroxyaromatic carbon sources (i.e. 3-hydroxybenzoate, 4-hydroxybenzoate, and hydroquinone). The log_2_ fold change values are shown (S1 and S2 Tables). Note that the values that are not statistically significant (i.e. adjusted p-value > 0.05) are shown in parentheses. (C) LC-MS/MS analysis of protein extracts from *C*. *parapsilosis* CLIB214. The cells were pre-cultivated overnight in an S3OH medium, inoculated to SGal, S3OH, S4OH, and SHyd media, and grown to ~ 10^7^ cells/ml. Soluble proteins were extracted and analyzed by LC-MS/MS. Log_2_ values of mean LFQ intensity ratios are shown (S3 Table). For proteins that were not identified on all carbon sources the LFQ values imputed from a normal distribution were used in the calculation (indicated in parentheses).

In line with our previous reports [7,9,10], the RNA-Seq analysis showed that the genes encoding the enzymes catalyzing reactions in each pathway as well as the plasma membrane carriers facilitating the transport of hydroxybenzoates (Fig 3A) are co-regulated in a substrate-specific manner. Specifically, the GP cluster genes are highly upregulated (i.e. between 267- (*GFA1*) and 3,061-fold (*HBT1*)) in the cells assimilating 3-hydroxybenzoate, which is metabolized via the GP. These genes exhibit only minor changes in media containing 4-hydroxybenzoate, except for *GTF1* and *HBT1* showing about 12.6- and 4.7-fold induction on this substrate, respectively (Fig 3B and S1 Table). The genes for the 3-OAP enzymes and two plasma membrane transporters (*HBT2* and its paralog *HBT3*) are highly upregulated on both 4-hydroxybenzoate (i.e. between 46.5- (*OSC1*) to 1,090-fold (*HBT2*)) and hydroquinone (i.e. between 8.1- (*HBT3*) and 208-fold (*HDX1*)). Expression of these genes changes only slightly on the GP substrate, except *MNX1* which exhibits about 19-fold increase (Fig 3B and S1 Table).

Next, we analyzed the proteins in the cellular extracts prepared from the CLIB214 cultures by LC-MS/MS. In total, we identified 1451 proteins, of which 1176 had significantly different relative abundance (based on LFQ values) as evaluated by ANOVA test. The comparison of the 3-OAP and GP related proteins identified in the cells assimilating hydroxyaromatic substrates with those utilizing galactose shows a pattern similar to the RNA-Seq results, i.e. the 3-OAP enzymes are highly enriched on both 4-hydroxybenzoate and hydroquinone, and the GP enzymes are enriched on 3-hydroxybenzoate (Fig 3C and S3 Table). However, we did not identify several proteins (i.e. Hbt3p, Hbt4p, Gtf1p, Otf1p). We presume that this is caused by overall low abundance of these polypeptides in the cells or their depletion from the prepared extracts due to insolubility or subcellular localization.

The MGCs contain yet uncharacterized genes *FRD1* and *GFA1* which are highly induced on hydroxyaromatic substrates and appear to be co-regulated with the genes for the 3-OAP or GP enzymes, respectively. This indicates that their products could participate in the metabolism of hydroxyaromatic substrates. Based on the expression profiles and identified protein domains, we hypothesize that *FRD1* (flavin reductase 1; *CANPARB_p44520-A* (*CPAR2_406430* in CDC317)) and *GFA1* (glutathione-dependent formaldehyde-activating enzyme 1; *CANPARB_p50380-A* (*CPAR2_704360* in CDC317)) code for maleylacetate reductase and glutathione-dependent maleylpyruvate isomerase involved in the 3-OAP and GP, respectively. Moreover, the transcriptome analysis also revealed that two neighboring open reading frames (ORFs) *CANPARB_p44920-A* and *CANPARB_p44910-A* also belong to highly upregulated genes (i.e. 83- and 103-fold, respectively) in CLIB214 cells assimilating 3-hydroxybenzoate compared to galactose. These ORFs are not annotated in the reference genome (CDC317), although their sequences are identical in both strains. The deduced amino acid sequences of CANPARB_p44920-A and CANPARB_p44910-A are highly similar to the N- and C-terminal half, respectively, of bacterial proteins from the amidohydrolase superfamily (S1 Fig, see below for a more detailed discussion). This protein family includes 2-amino-3-carboxymuconate-6-semialdehyde decarboxylase (ACMSD), 2,3-dihydroxybenzoate decarboxylase, 2,4-dihydroxy-6-methylbenzoate (orsellinate) decarboxylase (OrsB), 6-methylsalicylate decarboxylase (YanB), and 2-hydroxybenzoate (salicylate) decarboxylase involved in metabolism of various hydroxyaromatic substrates and, in some fungi, the corresponding genes are present in the MGCs linked to degradation of phenolic compounds [22,24].

### Metabolic pathways activated in cells assimilating hydroxyaromatic compounds

**Fig 4:**
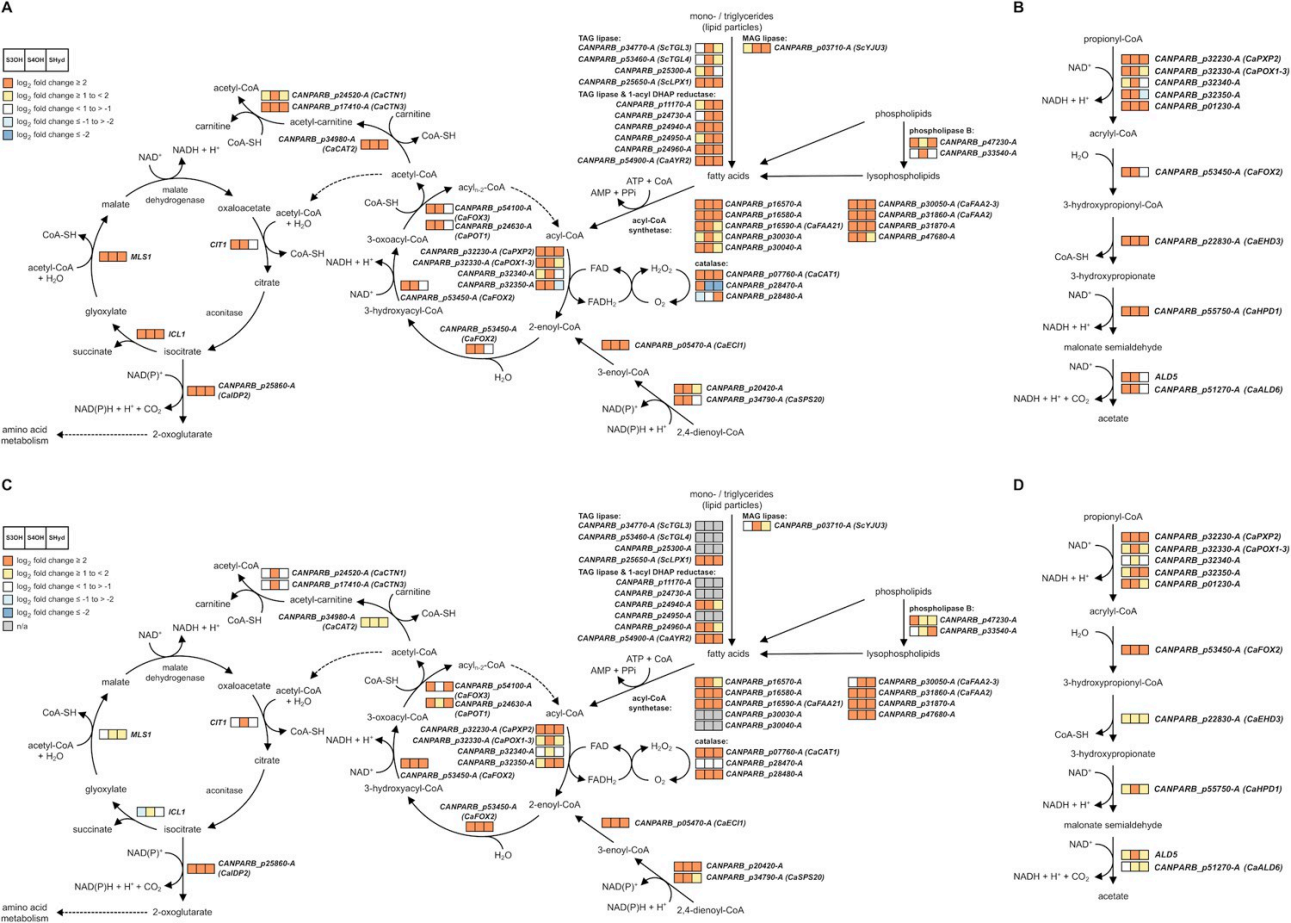
Major metabolic pathways upregulated in the cells utilizing 3-hydroxybenzoate, 4-hydroxybenzoate or hydroquinone as a sole carbon source. (A,C) The glyoxylate cycle, β-oxidation, and (B,D) modified β-oxidation pathway [35] are depicted in a simplified form. The expression profiles obtained by the RNA-Seq (A,B; S1 Table) and LC-MS/MS analyses (C,D; S3 Table) are shown. The three squares illustrate the gene expression changes on different hydroxyaromatic substrates compared to galactose as indicated in the legend in the upper left corner on panels (A) and (C). Only the genes whose transcription was overexpressed on at least one hydroxyaromatic substrate are shown. Note that not all enzymes listed in S2 and S6 Figs are shown on the scheme.

To identify metabolic enzymes and the corresponding pathways, we examined the lists of genes that were highly upregulated on the hydroxyaromatic substrates (Fig 2 and S1 Table). Using KEGG mapper we identified 163 metabolic enzymes upregulated at least on one hydroxyaromatic substrate (S2 Fig). In addition, searches using FungiDB revealed enrichment of several metabolic pathways involved in the metabolism of FA, amino acids, aromatic compounds, butanoate, propanoate, glyoxylate and dicarboxylate (S4 Table and Fig 4). A large proportion of the enzymes participating in FA metabolism [25] include those involved in β-oxidation and lipases mediating mobilization of FAs from mono- and triglycerides (MAG and TAG lipases) and (lyso)phospholipids (phospholipases B/A2). FA degradation is accompanied by production of hydrogen peroxide and the resulting oxidative stress is likely reduced by upregulation of the genes for catalases, superoxide dismutase, and glutathione-*S*-transferases. Although the subcellular localization of these proteins was not tested experimentally, our results confirm the increased catalase activity on hydroxyaromatic substrates (S3 Fig and S5 Table) supporting its protective role in β-oxidation [26]. Furthermore, the β-oxidation cycle is provided by acyl-CoA by the action of fatty acid-CoA synthetases (FAAs) that constitute an unusually large family of isoenzymes in *C*. *parapsilosis*. In *Saccharomyces cerevisiae*, there are four FAAs with various roles in FA metabolism, transport, acylation of proteins, vesicular transport, and transcription regulation [27]. *C*. *albicans*, *C*. *auris*, *C*. *dubliniensis*, and *C*. *glabrata* contain up to five FAAs, whereas *C*. *parapsilosis* contains twelve FAA-encoding genes. Nine of them are highly upregulated (log_2_ fold change > 2) on at least one substrate and five on all three tested hydroxyaromatic carbon sources (S4 Fig). Acetyl-CoA resulting from β-oxidation is feeding downstream metabolic processes including glyoxylate cycle. Indeed, the genes for citrate synthase (*CIT1*), isocitrate lyase (*ICL1*), and malate synthase (*MLS1*) are upregulated and so CoA can be provided back to β-oxidation [28]. The gene encoding a peroxisomal CoA diphosphatase (*PCD1*) regenerating CoA within peroxisomes [29] is also upregulated. In addition, the genes encoding enzymes involved in carnitine shuttle, such as *CAT2* encoding a homolog of a major form of carnitine acetyltransferase with dual localization to mitochondria and peroxisomes, are upregulated supplying a shuttle of acetyl units between these organelles [30]. Finally, upregulated genes for enzymes involved in metabolism of amino acids, vitamins, purines, and pyrimidines contribute to the metabolic needs of the cells utilizing hydroxyaromatic substrates (S2 Fig). In many cases, the gene expression profiles based on the RNA-Seq experiment more or less correspond to those obtained by the LC-MS/MS analysis. However, there are several notable differences. In particular, the genes coding for the three glyoxylate cycle enzymes, namely citrate synthase (Cit1p), isocitrate lyase (Icl1p), and malate synthase (Mls1p) exhibit upregulated transcription (log_2_ fold change ≥ 2) both on 3-hydroxybenzoate and 4-hydroxybenzoate, yet the LC-MS/MS analysis indicates a slight decrease of the corresponding proteins on the former substrate. Discordances between mRNA and protein levels are usually caused by posttranscriptional regulation of protein synthesis and/or degradation [31,32]. The observed differences in transcriptome and proteome profiles imply that the interconnection of final products of the GP and 3-OAP with the intermediate metabolism differs. The last step of the GP occurs in cytosol [9] producing pyruvate and fumarate. The former could be carboxylated to oxaloacetate [33] thus supplying the substrate for sugar synthesis in the same compartment. On the other hand, the 3-OAP producing succinate and acetyl-CoA in mitochondria [11] needs the peroxisomal glyoxylate cycle to convert the latter C2 unit to C4 to supply the gluconeogenesis with a C4 substrate [34]. To support this conclusion, we constructed the *Δicl1/Δicl1* mutant lacking isocitrate lyase and examined its growth on the 3-OAP and GP substrates. We demonstrate that this mutant is unable to grow on both 4-hydroxybenzoate and hydroquinone, yet its growth on 3-hydroxybenzoate is only slightly reduced compared to the wild type cells (S5 Fig).

### Peroxisomes participate in the cellular adaptation to hydroxyaromatic compounds

**Fig 5:**
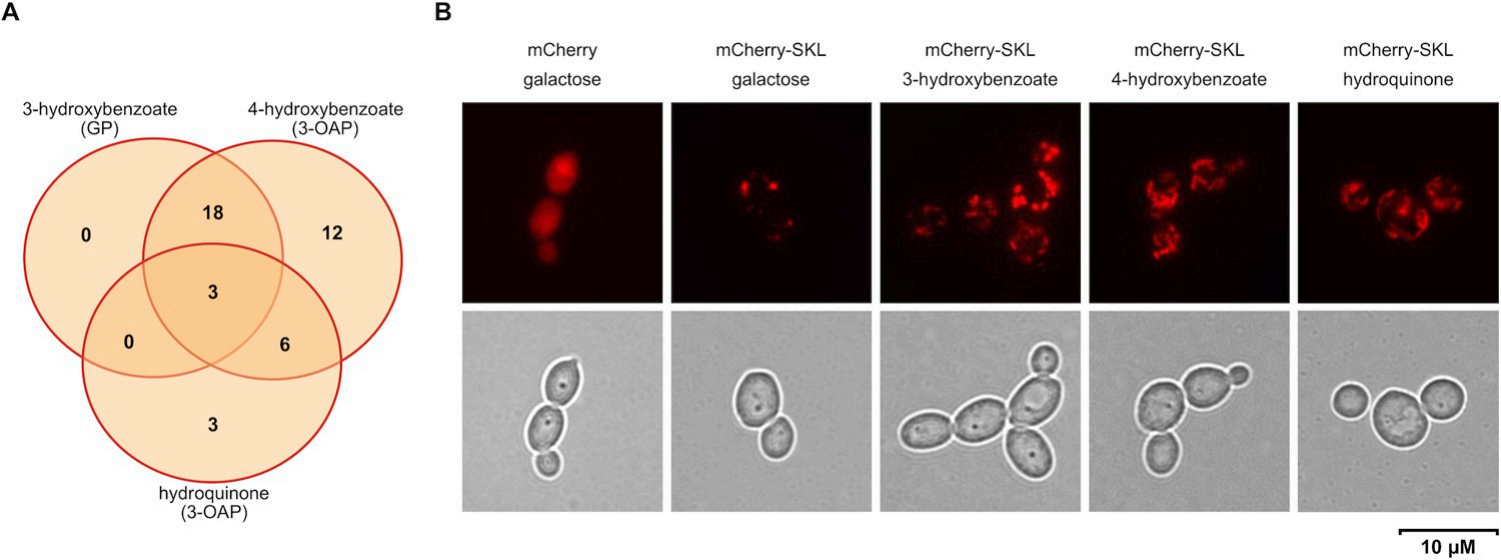
Peroxisomes are involved in the catabolism of hydroxyaromatic compounds. (A) The Venn diagram illustrating numbers of highly upregulated genes (log_2_ fold change ≥ 2; adjusted p-value ≤ 0.05) involved in the metabolism and biogenesis of peroxisomes identified by RNA-Seq analysis of CLIB214 cells assimilating 3-hydroxybenzoate, 4-hydroxybenzoate or hydroquinone compared to galactose (S1 Table). The diagram was drawn with a web tool (http://bioinformatics.psb.ugent.be/webtools/Venn/). (B) *C*. *parapsilosis* CDU1 cells expressing cytosolic (mCherry) and peroxisomal (mCherry-SKL) versions of the marker protein. The cells were transformed with pBP7-mCherry or pBP7-mCherry-SKL plasmids and the transformants were cultivated on a synthetic medium with galactose, 3-hydroxybenzoate, 4-hydroxybenzoate or hydroquinone at 28°C.

Previously, we reported that catabolism of hydroxyaromatic compounds is linked to mitochondria [9]. Here we show that peroxisomes also play a role in cellular response to these substrates. These organelles are highly dynamic and tightly regulated by processes of *de novo* formation, division, and autophagic degradation. In yeast cells, their number depends on the utilized carbon source [36]. The GO cellular component analysis showed that the lists of genes highly upregulated on the hydroxyaromatic substrates (S1 Table) are enriched for peroxisome-related categories (S6 Table). The fact that boosting FA catabolism in the cells assimilating hydroxyaromatic substrates is accompanied by proliferation of peroxisomes is underlined not only by upregulation of genes for metabolic enzymes, but also those involved in peroxisome biogenesis (Figs 5A and S6) including Pex11p involved in peroxisome proliferation [37,38], Pex3p and Pex19p essential for the formation of peroxisomal membrane [39], receptor Pex5p and the components of matrix protein importomer, namely Pex1p, Pex2p, Pex4p, Pex12p, Pex13p, and Pex14p [40]. In addition, the gene coding for inheritance protein Inp1p which secures a balanced distribution of peroxisomes between mother and daughter cells is also upregulated [41]. To demonstrate the presence of peroxisomes in cells assimilating the 3-OAP and GP substrates, we constructed the plasmid pBP7-mCherry-SKL expressing a soluble codon-optimized mCherry protein [42] tagged with peroxisomal targeting signal type 1 (PTS1) serine–lysine–leucine (SKL) at its C-terminus. The plasmid pBP7-mCherry expressing an unmodified marker was used as a control. Both plasmids were introduced into *C*. *parapsilosis* CDU1 cells and the transformants were grown in synthetic media containing galactose. Cells containing pBP7-mCherry-SKL were also cultivated in synthetic media containing 3-hydroxybenzoate, 4-hydroxybenzoate or hydroquinone as a sole carbon source. Examination of the transformants by fluorescence microscopy showed the presence of multiple bright foci in the cells expressing mCherry-SKL protein, while the control cells carrying the pBP7-mCherry plasmid show cytosolic localization of the marker. This result indicates that cells utilizing hydroxyaromatic substrates metabolized via the 3-OAP or GP contain multiple peroxisomes, whose number seems to be modestly increased compared to the cells growing on galactose (Fig 5B).

### *C*. *parapsilosis* response to hydroxybenzenes and hydroxybenzoates

**Fig 6:**
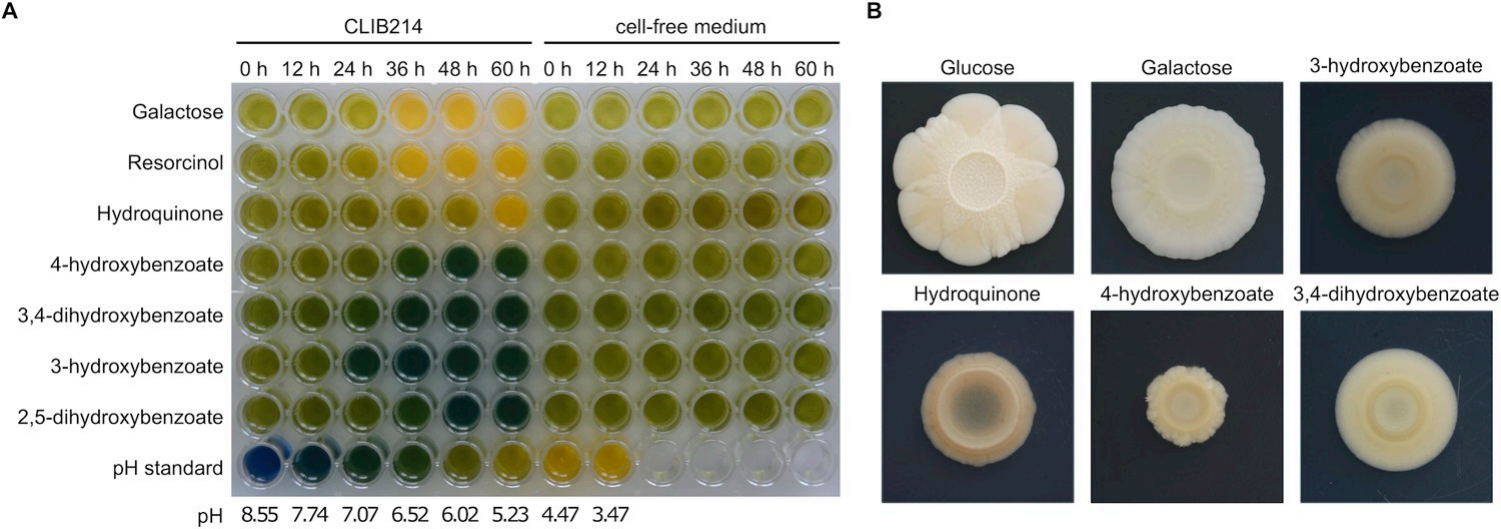
*C*. *parapsilosis* cells assimilating hydroxyaromatic substrates exhibit pH changes of the cultivation media and differences of colony morphology. (A) CLIB214 cells assimilating hydroxyaromatic substrates alter the pH of cultivation media. The cells were cultivated in liquid synthetic media containing indicated carbon source and bromothymol blue as pH indicator (see Materials and Methods for details). (B) Morphology of CLIB214 colonies cultivated for 30 days at 28°C in synthetic media containing indicated carbon source.

The RNA-Seq analysis of the cells utilizing 3-hydroxybenzoate, 4-hydroxybenzoate or hydroquinone shows that although there is a group of ninety nine genes upregulated on any of the three carbon sources, many genes are selectively induced only on a single substrate (Fig 2A). As 3-hydroxybenzoate and 4-hydroxybenzoate are catabolized by distinct biochemical pathways producing different metabolites (i.e. acetyl-CoA and succinate in the 3-OAP, fumarate and pyruvate in the GP), the differences in transcription profiles of cells utilizing these substrates may reflect, at least in part, different links of these pathways to central metabolism. However, 4-hydroxybenzoate and hydroquinone are degraded in the same pathway (i.e. 3-OAP), yet only about a half of the upregulated genes are induced on both substrates and the difference in the lists of downregulated genes on these substrates is even greater (Fig 2). In the 3-OAP, hydroxybenzoates are decarboxylated to hydroxybenzenes (i.e. 4-hydroxybenzoate to hydroquinone; 2,4-dihydroxybenzoate and protocatechuate to hydroxyhydroquinone). The decarboxylation step is catalyzed by the monooxygenase Mnx1p which has broad substrate specificity [43,44]. This reaction releases a molecule of carbon dioxide, which can be readily converted by carbonic anhydrase to a bicarbonate anion (HCO_3_^-^). Carbon dioxide, carbonic acid and bicarbonate anions are components of a buffering system that may affect the pH in cultivation media. To monitor the pH changes we cultivated CLIB214 cells in synthetic media containing various carbon sources and a pH indicator (bromothymol blue, p*K*a = 7). We observed dramatic pH changes in the cultures grown on hydroxybenzenes compared to those assimilating hydroxybenzoates. As judged from the color of the pH indicator observed at later cultivation stages (> 12 hours), the media were acidified when the cells assimilated hydroquinone or resorcinol, which is also typical for sugar utilization [45]. In contrast, the cells utilizing hydroxybenzoates (i.e. 4-hydroxybenzoate, protocatechuate, 3-hydroxybenzoate, gentisate) alkalinized the medium (i.e. the pH increased from 6.1 to about 7.1) (Fig 6A). Although the observed differences in the pH of cultivation media cannot be explained solely by the buffering effect of bicarbonate, these anions have a role in intracellular signaling (via activation of adenylyl cyclase) involved the control of metabolism, phenotypic switching, and morphology [46]. Metabolic changes, including those associated with changes in external pH, affect the development of yeast colonies [47]. Indeed, the size and morphology of the *C*. *parapsilosis* colonies grown in synthetic media indicate that the cells respond differently to the utilized carbon source (Fig 6B). These results illustrate that assimilation of hydroxyaromatic substrates triggers a global cellular response, reflected by changes in morphogenesis and/or cell differentiation, which depends on utilized hydroxyaromatic compound.

### *OTF1* and *GTF1* code for Zn(II)_2_Cys_6_ transcription activators involved in the control of the 3-OAP and GP genes, respectively

As mentioned above the 3-OAP and GP gene clusters contain the genes *OTF1* and *GTF1*, respectively, coding for putative transcription factors. The predicted proteins are 963 and 741 amino acids long, respectively, and contain Gal4-like Zn(II)_2_Cys_6_ zinc cluster DNA-binding and fungal transcription factor domains as well as putative nuclear localization signals (NLS) indicating their import into the cell nucleus (S7 Fig). As the orthologs of these genes are conserved in several species belonging to the ‘CUG-Ser1’ clade (see below) which assimilate hydroxyaromatic compounds [7–9] we hypothesized that *OTF1* (3-oxoadipate pathway transcription factor 1; *CANPARB_p44550-A* (*CPAR2_406460* in CDC317)) and *GTF1* (gentisate pathway transcription factor 1; *CANPARB_p50390-A* (*CPAR2_704370* in CDC317)) control the expression of corresponding MGC.

**Fig 7:**
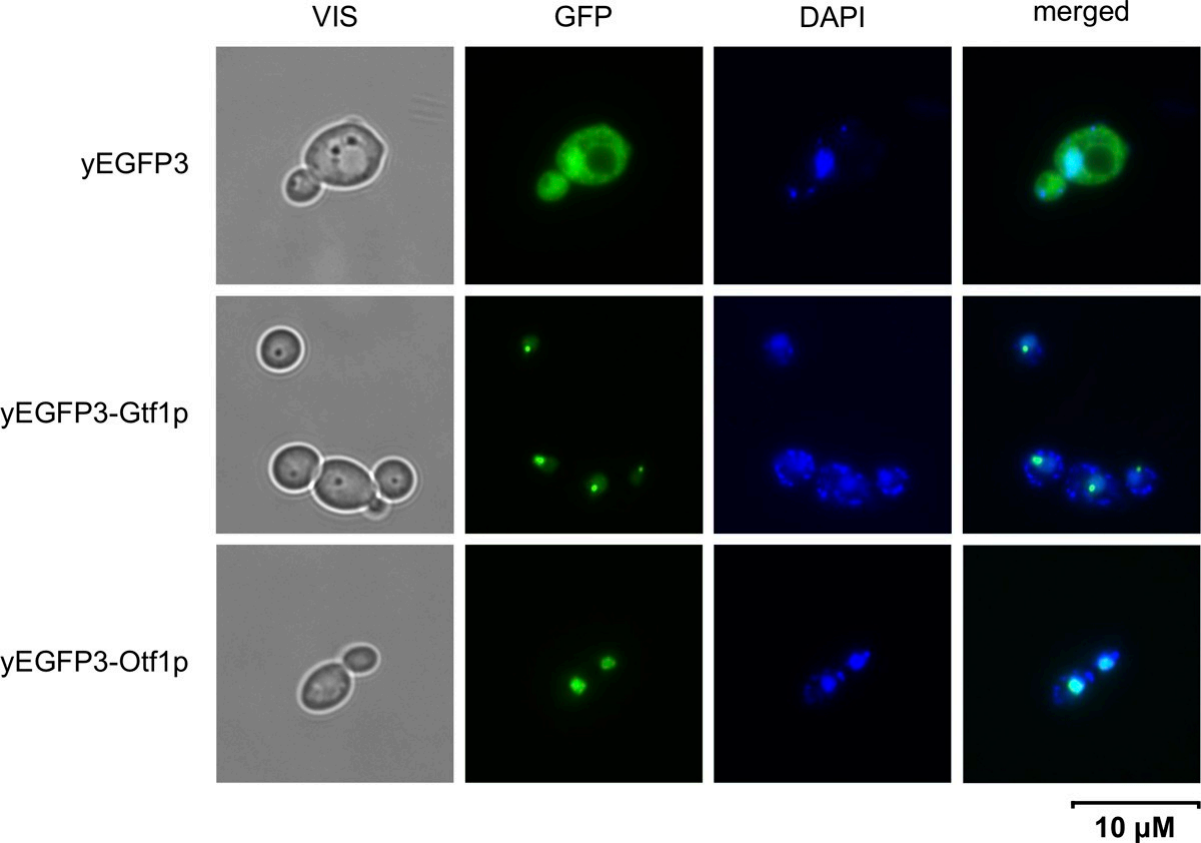
Transcription factors Otf1p and Gtf1 tagged with yEGFP3 at their N-termini exhibit nuclear localization. *C*. *parapsilosis* SR23 *met-1* cells transformed with pPK6, pPK6-OTF1 or pPK6-GTF1 plasmids were pre-grown overnight in an SD medium, washed with water, inoculated into an SGal medium, and cultivated overnight. DNA in cells was stained with DAPI.

First, we confirmed that Otf1p and Gtf1p are targeted into the cell nucleus. We prepared the plasmid constructs expressing these proteins tagged with yEGFP3 at their N-termini (i.e. yEGFP3-Otf1p, yEGFP3-Gtf1p) in *C*. *parapsilosis* SR23 *met-1* cells. Examination by fluorescence microscopy showed that both proteins co-localize with DAPI-stained nuclear DNA. Moreover, yEGFP3-Gtf1p appears to be concentrated in distinct foci pointing to its specific subnuclear localization possibly reflecting its association with the regulatory regions of its target genes located in the subtelomeric region of chromosome 6 (Fig 7). However, we cannot exclude a possibility that the yEGFP3-Gtf1p foci represent an aggregate formed in the perinuclear region from mis-folded fusion proteins.

**Fig 8:**
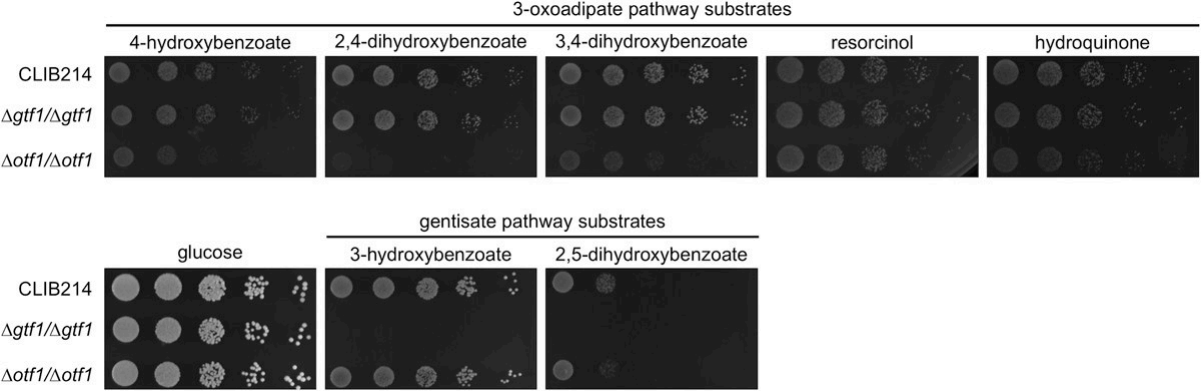
*C*. *parapsilosis* mutants *Δotf1*/*Δotf1* and *Δgtf1*/*Δgtf1* exhibit impaired growth on hydroxyaromatic substrates. Indicated strains were pre-grown overnight in a complex medium (YPD) at 28°C, washed with water and resuspended to ~ 6×10^6^ cells/ml. Serial fivefold dilutions were then spotted on solid synthetic media containing indicated carbon sources. The plates were incubated for 4 days at 28°C.

To demonstrate that Otf1p and Gtf1p are involved in the transcriptional control of the 3-OAP and GP, respectively, we constructed knockout strains lacking both alleles of *OTF1* or *GTF1* and tested their ability to utilize different hydroxybenzenes and hydroxybenzoates as a sole carbon source. We found that the *Δotf1*/*Δotf1* mutant exhibits a growth defect on several substrates metabolized via the 3-OAP. While its growth is impaired in media containing hydroxybenzoates (i.e. 4-hydroxybenzoate, 2,4-dihydroxybenzoate, 3,4-dihydroxybenzoate), we did not observe a growth defect in media containing hydroxybenzenes (resorcinol, hydroquinone). On the other hand, the *Δgtf1*/*Δgtf1* mutant is unable to grow in media containing 3-hydroxybenzoate or gentisate, which are degraded via the GP (Fig 8). The phenotypes of both mutants indicate that Otf1p and Gtf1p are involved in the control of the 3-OAP and GP, respectively.

To investigate the role of Otf1p and Gtf1p in the control of the 3-OAP and GP genes, respectively, we compared the transcriptomic profiles of the knockout mutants with the parental strain CPL2H1 cultivated in synthetic medium containing a mixture of hydroxyaromatic carbon sources (i.e. 3-hydroxybenzoate, 4-hydroxybenzoate, and hydroquinone) metabolized via the GP or 3-OAP. The RNA-Seq experiment demonstrated that expression of the genes present in the GP gene cluster is substantially decreased in the *Δgtf1*/*Δgtf1* mutant compared to the parental strain (Fig 3 and S2 Table and S8 Fig). In addition, we found that the transcript(s) derived from *CANPARB_p44920-A* and *CANPARB_p44910-A* ORFs coding for a predicted amidohydrolase superfamily protein is almost absent in this mutant.

A comparison of the *Δotf1*/*Δotf1* mutant and CPL2H1 cells revealed more subtle differences in the expression of genes for the 3-OAP enzymes. We found that *MNX1* and *HBT2* are downregulated by 6.37- and 1.97-fold, respectively (Fig 3 and S2 Table and S8 Fig). As these genes code for 4-hydroxybenzoate 1-hydroxylase decarboxylating hydroxybenzoates to hydroxybenzenes [7,43,44] and a hydroxybenzoate transporter [10], their decreased expression goes in line with the observation that the *Δotf1*/*Δotf1* mutant has impaired growth on hydroxybenzoates (Fig 8). The expression of the genes *MNX3*, *HDX1*, *FRD1*, *OSC1*, and *OCT1* encoding remaining enzymes of the 3-OAP is also slightly decreased (i.e. by 1.45 to 1.90-fold). However, as the mutant grows on media with hydroquinone or resorcinol, we assume that expression of these genes is sufficient for utilization of both hydroxybenzenes.

### Otf1p and Gtf1p recognize specific motifs in promoter sequences

As described above, the genes coding for the enzymes of the 3-OAP and GP are highly upregulated in the cells assimilating hydroxyaromatic substrates. To identify potential regulatory motifs involved in their transcriptional control, we searched corresponding promoter sequences for putative Otf1p- and Gtf1p-binding sites. Both transcription factors belong to the Gal4-like family whose members recognize sequences containing CGG triplets oriented as inverted repeats separated by a distinct number of nucleotides, although other terminal nucleotides such as GGA were also identified in the binding sites [48,49]. Moreover, Otf1p is a homolog of the transcription factor *qa-1F* activating expression of the quinic acid gene cluster in *Neurospora crassa*, which recognizes a 16-mer motif GGATAATCGATTATCC [50]. The search of the *MNX1* promoter revealed a similar motif GGRN_10_WCC (S9 and S10 Figs) which may represent a binding site for the transcription factor Otf1p. This is also supported by the presence of this motif in the upstream regions of the other genes coding for the 3-OAP enzymes (i.e. *FRD1*, *HDX1*, *MNX3*, *OCT1*, *OSC1*), hydroxybenzoate transporter *HBT2* and its paralog *HBT3*, which are co-induced in the cells grown in media with 4-hydroxybenzoate or hydroquinone (Fig 3), although in some cases the motif is present on the opposite strand (S9 Fig). Similarly, we searched the promoter regions of the GP cluster genes for putative Gtf1p-binding sites. We identified a motif GGAN_7_TCC which occurs upstream of each ORF in the GP gene cluster, except for the *GTF1* gene (S9 and S10 Figs). This motif is also present upstream of *CANPARB_p44920-A*, which along with the GP cluster genes is also highly induced in media containing 3-hydroxybenzoate (Fig 3).

As our previous studies [7,9,10] indicated that the 3-OAP and GP genes are repressed in media containing glucose, we also searched the promoter sequences for sequence motifs potentially mediating this process. We have found several copies of the SYGGRG motif which is recognized by transcriptional repressors Mig1/Mig2 both in *S*. *cerevisiae* and *C*. *albicans* [51–54]. Some of these sites (e.g. -139 to -134 and -108 to -103 upstream of *MNX2* and *HBT1* ORFs, respectively) are located near an A/T-box, which is known to be associated with bending of a DNA molecule upon Mig1/Mig2-binding [51] supporting the idea that at least some of the SYGGRG sites are functional in *C*. *parapsilosis*.

**Fig 9:**
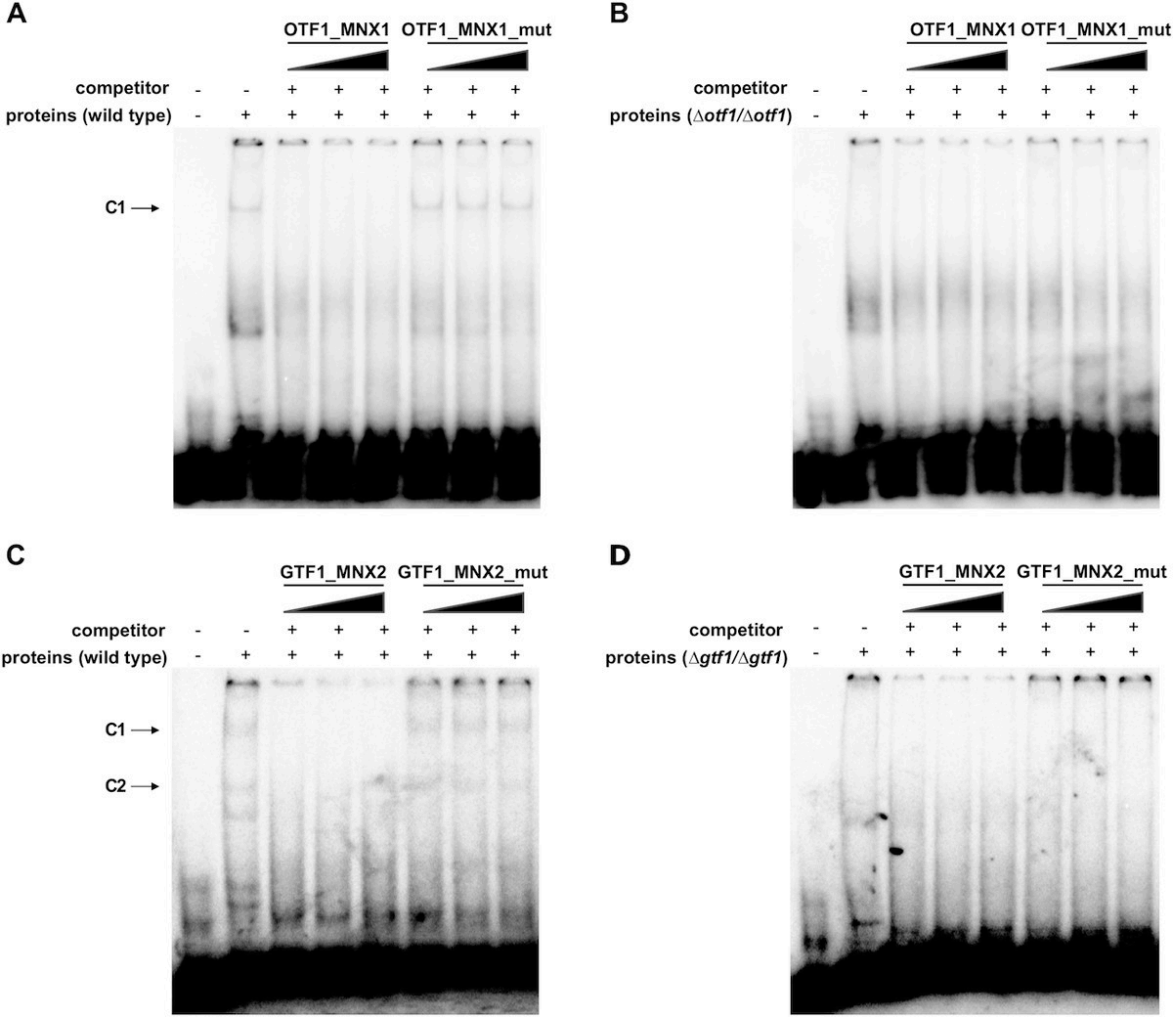
Transcription factors Otf1p and Gtf1p bind to predicted sequence motifs. The EMSA experiments were performed using the protein extracts prepared from CPL2H1 (A),(C), *Δotf1*/*Δotf1* (B), and *Δgtf1*/*Δgtf1* (D) cells and the 5’ end-labeled dsDNA probes containing the predicted Otf1p-binding site from the *MNX1* promoter (OTF1_MNX1; (A),(B)) or the Gtf1p-binding site from the *MNX2* promoter (GTF1_MNX2; (C),(D)). The ds-oligonucleotide competitors containing either the wild type (OTF1_MNX1, GTF1_MNX2) or mutated binding motifs (OTF1_MNX1_mut, GTF1_MNX2_mut) were used with increasing amounts of 100, 300, and 500 ng as indicated above lanes.

To demonstrate that transcription factors Otf1p and Gtf1p recognize the predicted motifs, we performed EMSA experiments using the protein extracts prepared from the wild type cells (CPL2H1) as well as the mutants lacking a functional copy of the corresponding transcription factor and the labeled ds-oligonucleotide probes OTF1-MNX1 and GTF1-MNX2 derived from the promoters of *MNX1* and *MNX2* genes, respectively. These probes contain a single copy of the predicted binding site. In DNA-binding reactions performed using the extract from the wild type cells we identified one and two bands using the probes OTF1-MNX1 and GTF1-MNX2, respectively (Figs 9 and S11). As these bands were absent when the extracts were prepared from the mutant cells (i.e. *Δotf1/Δotf1* for OTF1-MNX1; *Δgtf1/Δgtf1* for GTF1-MNX2), we assume that they correspond to the DNA-protein complexes containing the corresponding transcription factor and the probe. To further support this idea, we showed that the 50-fold and higher molar excess of unlabeled oligonucleotide used in the assay as a specific competitor outcompetes the labeled probe from the complex. Importantly, the oligonucleotides OTF1-MNX1_mut and GTF1_MNX2_mut carrying alterations in the conserved positions of predicted binding sites (i.e. TTTN_10_TAA and TTTN_7_AAA, respectively), did not interfere with the complex formation.

Taken together, we demonstrate that Otf1p and Gtf1p are Gal4p-like transcription factors present in the extracts from the wild type cells and they specifically bind to DNA fragments carrying the motifs GGRN_10_WCC and GGAN_7_TCC, respectively. Gtf1p appears as the main transcriptional activator of the GP gene cluster. On the other hand, although Otf1p contributes to transcriptional activation of the 3-OAP genes, it predominantly controls the expression of *MNX1* encoding decarboxylating mononoxygenase [7,44]. This conclusion is supported by differences in the gene expression profiles of the cells grown on 4-hydroxybenzoate compared to those assimilating hydroquinone (Fig 3) and the growth phenotypes (Fig 8) and underscores the physiological differences in catabolic degradation of hydroxybenzenes and hydroxybenzoates. These results imply that, besides Otf1p, activation of the 3-OAP genes requires additional transcription factor(s). As 4-hydroxybenzoate and hydroquinone differ by the presence of carboxyl group, we speculate that bicarbonate anions generated upon Mnx1p-catalyzed decarboxylation of 4-hydroxybenzoate and corresponding cellular response may also contribute to identified differences.

### Phylogenetic analyses

**Fig 10:**
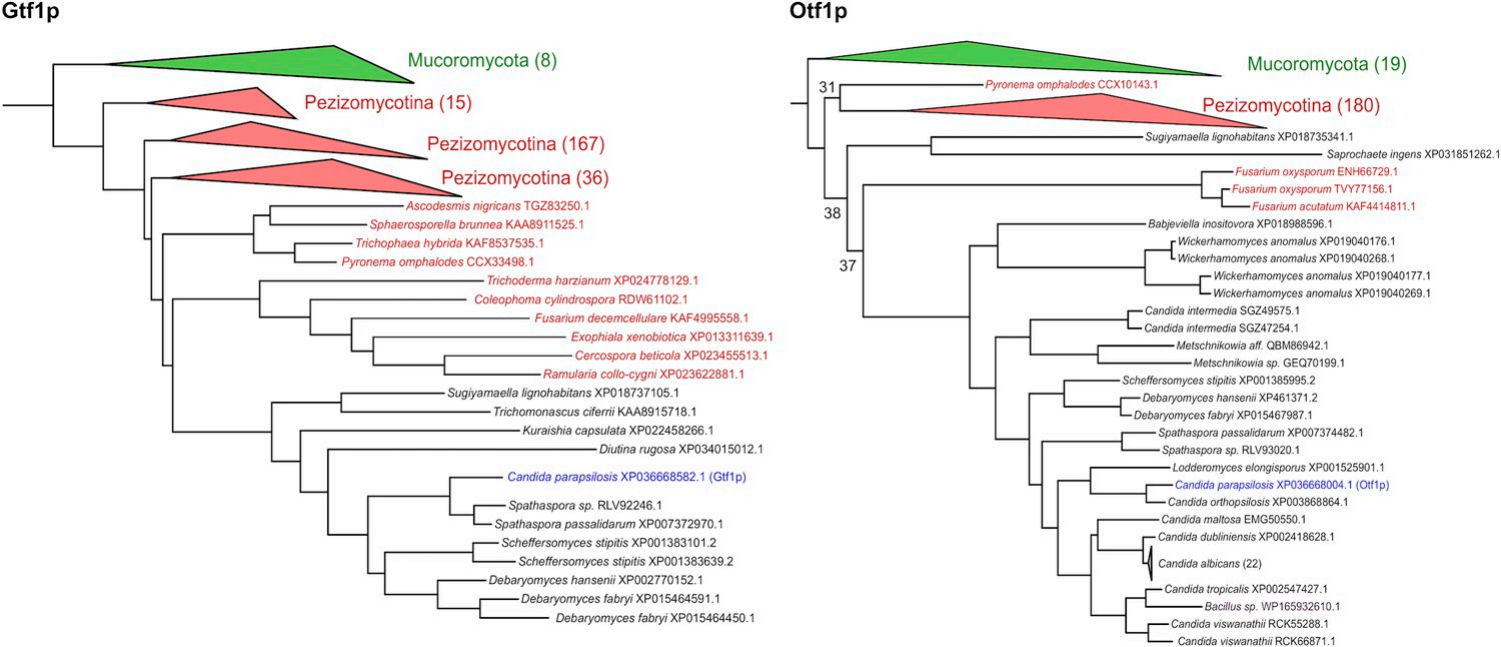
Phylogenetic relationships of the transcription factors Gtf1p (left), Otf1p (right), and their closest homologs. For simplicity, some monophyletic tree partitions including sequences from the same taxonomic classification are collapsed and their number is indicated in brackets. Lowly supported nodes (<80) are displayed indicating the specific branch support. Mucoromycota sequences or partitions are indicated in green, Pezizomycotina sequences and partitions are indicated in red, Saccharomycotina sequences are indicated in black, and the *C*. *parapsilosis* sequence used as a seed in the blast searches is indicated in blue. Note several *C*. *albicans* sequences likely correspond to redundant sequences from different strains or sequencing projects. Note as well WP_165932610 sequence assigned to a *Bacillus* strain, which could correspond to a taxonomic miss-assignment or contamination. The full phylogenetic trees in newick format, including all sequence names and branch support is provided as supplemental information (S1 and S2 Texts).

The phylogenetic relationships of the transcription factors Otf1p and Gtf1p, as well as the twelve FAAs in *C*. *parapsilosis* were first assessed by investigating pre-computed phylogenies and orthology and paralogy relationships in MetaPhORs v2 [55] and PhylomeDB v4 [56] as of October 2020. As Otf1p and Gtf1p display a very sparse distribution among Saccharomycotina, we performed new phylogenetic reconstructions (see Materials and Methods) with the first 250 best Blast hits (e-value < 10^−20^) in a search against NCBI non-redundant database (as of October 2020). *GTF1* phylogeny (Fig 10) closely resembles that previously reported for other genes of the GP cluster such as *GDX1* [9], with a sparse distribution within Saccharomycotina and closely related to Pezizomycotina and Mucoromycota sequences. Importantly, this cluster was found to be significantly conserved between Saccharomycotina, Pezizomycotina, and some Mucoromycota species in an earlier large-scale analysis of gene order conservation in fungi (cluster CF_000060 in [5]). *OTF1* phylogenetic reconstruction reveals a somewhat broader distribution within Saccharomycotina but also a close relationship with Pezizomycotina sequences (Fig 10). Similar patterns were obtained when relaxing Blast filters in the initial search (e-value < 10^−10^) or when performing phylogenetic analysis restricted to all members of the fungal orthogroups to which these species belong according to the EggNOG database (ENOG503NX3W for OTF1, ENOG503NYPS for GTF1, see S12 Fig).

Previously we proposed that the 3-OAP variant occurring in *C*. *parapsilosis* emerged in an ancestral lineage before the divergence of the ‘CUG-Ser1’ clade from other Saccharomycotina lineages by an upgrade of a shorter version of this pathway (such as seen in *C*. *albicans*), which allows degradation of only hydroxybenzenes [10]. The principal difference between the two variants is the presence of both the Mnx1p-catalyzed decarboxylation step and the functional uptake of hydroxybenzoates provided by Hbt2p and possibly also by its paralogs Hbt3p and Hbt4p, in the longer 3-OAP version. The acquisition or co-option of *OTF1* might have served the need for a specific regulation of this upgraded pathway. Differences in the transcriptional control of *MNX1* and to some extent also *HBT2* and *HBT3* compared to remaining 3-OAP genes (see above) supports the upgrade scenario and provides additional insight into the evolution of this pathway.

The evolutionary relationships of the twelve *FAA* genes in *C*. *parapsilosis* is well represented in PhylomeDB trees (see a simplified example in S4 Fig). This reveals an intricate evolution of this family with at least ten nested gene duplications at different ages leading to the twelve paralogs present in *C*. *parapsilosis* and with complex one-to-many orthology and paralogy relationships with the four *FAA* genes present in *S*. *cerevisiae* and *C*. *albicans*. This highlights a dynamic gene copy evolution leading to complexification of the FA metabolism in the *C*. *parapsilosis* clade.

Finally, we investigated the possible origin of the putative amidohydrolase gene (*CANPARB_p44920-A* and *CANPARB_p44910-A*) identified in this work. PhylomeDB searches rendered no results, but MetaPhOrs identified an ortholog in *C*. *metapsilosis* (g2237) sharing 64% protein identity. The *C*. *metapsilosis* gene has a single reading frame indicating that in *C*. *parapsilosis* ancestor the gene was split up into two ORFs by an in-frame stop codon UGA. In general, this alteration would inactivate a gene function, although stop codon bypassing or readthrough events [57] could generate a full-length protein corresponding to the polypeptide translated from uninterrupted ORF. Although both *C*. *parapsilosis* ORFs are transcribed on 3-hydroxybenzoate and the transcript is regulated by transcriptional activator Gtf1p, we did not identify peptides derived neither from individual ORFs nor from a deduced full-length protein by LC-MS/MS analysis. Searches in NCBI non-redundant database identified only bacterial sequences among the top 500 hits, with the best matches belonging to various *Pseudomonas* species with e-values ranging from 10^−105^ to 10^−102^ and sequence identities between 49 and 53% at the protein level. A multiple sequence alignment of the first 100 hits and the two *Candida* sequences revealed conservation of numerous amino acid residues (S1 Fig). To validate these results using a phylogenetic approach, we used the amidohydrolase gene as a seed in an eggNOG-mapper search [58]. All resulting orthologs were from different Actinobacteria species further supporting the bacterial origin of this gene. We then extracted all 7549 sequences contained in the amidohydrolase orthologous group (COG2159) and computed a phylogenetic tree with FastTree [59]. Next, we refined the phylogenetic reconstruction around the relevant gene by extracting a highly supported subtree (local support value > 0.9) comprising the closest 250 sequences to the putative *C*. *parapsilosis* amidohydrolase gene and subjecting these sequences to a more exhaustive phylogenetic reconstruction with IQ-Tree [60]. The resulting tree (S13 Fig) separates fungal and bacterial species, with the amidohydrolase gene clearly falling within the Actinobacteria group.

This result indicates that this gene represents a relatively recent transfer of a gene encoding a putative amidohydrolase from an actinobacterial ancestor to the common ancestor of *C*. *parapsilosis* and *C*. *metapsilosis*. Alternatively, considering the relatively low sequence identity between the two transferred genes and their location in different, non-syntenic chromosomal locations in each of the species, two independent origins from a related bacterial donor might be postulated. The low levels of similarity exhibited between the transferred genes and their closest bacterial donors preclude us to pinpoint a specific bacterial species. Interestingly, when limiting the search to Saccharomycotina three significant hits appeared from sequences in the unrelated yeasts *Wickerhamiella sorbophila* (acc.no. XP_024665283.1), *Trichomonascus ciferrii* (KAA8915622.1), and *Naumovozyma castellii* (XP_003673849.1). However, their sequence identities with the *C*. *parapsilosis* protein were lower than that of the bacterial homologs (39%, 35%, and 29%, respectively), suggesting they are more distantly related. Indeed, searches using these other yeast proteins as queries identified other bacteria (for *W*. *sorbophila*) or non-overlapping species of Pezizomycotina fungi (for *T*. *ciferrii* and *N*. *castellii*) among the first 100 significant hits, suggesting each of these yeasts acquired a different amidohydrolase gene in independent horizontal gene transfers. Such recurrent horizontal gene transfer scenario is reminiscent of other metabolic genes including amino acid racemases, which are also present in *C*. *parapsilosis* and other yeast species [61].

## Materials and methods

### Yeast strains

*C*. *parapsilosis* strains CLIB214 (identical to the type strain CBS604), CPL2H1 (CLIB214 *Δleu2*/*Δleu2*, *Δhis1/Δhis1*; [62]), its mutant derivatives (*Δgtf1*/*Δgtf1*, *Δicl1*/*Δicl1*, and *Δotf1*/*Δotf1*; this study), CDU1 (CLIB214 *Δura3/Δura3*; [63]), and SR23 *met-1* (*ade*^*-*^, *lys4*^*-*^, *met2*^*-*^; [64]) were used in this study.

### DNA isolation and sequencing

Genomic DNA was isolated from the strain CLIB214. Briefly, a yeast culture grown overnight at 28°C in 100 ml of YPD medium (1% [wt/vol] yeast extract, 2% [wt/vol] peptone, 2% [wt/vol] glucose) at 28°C was harvested by centrifugation (5 min, 2,100 *g* at 4°C), the cells were resuspended in 20 ml of 2% [vol/vol] 2-mercaptoethanol and incubated for 30 min at room temperature. The spheroplasts were prepared in 6 ml of 1 M sorbitol, 10 mM EDTA (pH 8.0) containing 0.125 mg of Zymolyase 20T (Seikagaku) at 37°C, pelleted by centrifugation (5 min, 2,100 *g* at 4°C) and lysed in 3 ml of 0.15 M NaCl, 0.1 M EDTA (pH 8.0), 0.1% [wt/vol] SDS. Proteins were removed by three extractions with equal volume of phenol buffered with 10 mM Tris-HCl, 1 mM EDTA (pH 8.0) and by one extraction with equal volume of chloroform: isoamyl alcohol (24: 1). Nucleic acids were precipitated using 0.1 M NaCl and 1 volume of 96% [vol/vol] ethanol, pelleted by centrifugation (10 min, 16,100 *g* at 4°C), washed with 70% [vol/vol] ethanol and air dried. The precipitate was dissolved in 1 ml of 10 mM Tris-HCl, 1 mM EDTA (pH 7.5), 0.1 mg/ml RNase A and incubated for 45 min at 37°C. DNA was extracted by phenol and chloroform: isoamyl alcohol, precipitated using 0.1 M NaCl and 2 volumes of 96% [vol/vol] ethanol, washed with 70% [vol/vol] ethanol, air dried, dissolved in 150 μl 10 mM Tris-HCl, 1 mM EDTA (pH 7.5) and purified on a Genomic-tip 100/G (Qiagen) according to the manufacturer’s instructions. A paired-end (2×151-nt) TruSeq PCR-free DNA library was sequenced on a NovaSeq6000 platform at Macrogen Korea, yielding 81,578,508 reads (12.32 Gbp; 944x coverage). Nanopore sequencing was performed on a MinION Mk-1B device with an R9.4.1 flow cell using a Rapid barcoding kit (SQK-RBK004; Oxford Nanopore Technologies). 119,788 reads were obtained (mean and median lengths are 9,200.2 and 5,938 nucleotides, respectively) totaling 1.1 GBp (84x coverage). Nanopore reads were assembled by Canu version 1.9 [65], resulting in 20 contigs, which were manually examined. Chromosomes 1, 2, 3, 4 and 7 were used as assembled by Canu. Chromosome 8 was created by connecting two Canu contigs. In the contig corresponding to chromosome 5, a 8 kbp region was replaced by a longer 14.5 kb version from one of the excluded shorter contigs. This region contains two copies of the *PDR5* gene, possibly with a copy number variation. Finally, regions directly upstream and downstream of ribosomal DNA (rDNA) arrays on chromosome 6 were misassembled in the Canu assembly. These regions were replaced by sequences assembled from Illumina reads by SPAdes version 3.12 [66]. After these manual modifications, the entire assembly was polished first by Medaka version 0.11.5 [67] using nanopore reads, and then by three iterations of Pilon version 1.12 [68]. The rDNA repeat poses problems for polishing due to its repetitive nature, and thus a single copy of the repeat was polished separately by Pilon and then used in the final assembly. Mitochondrial DNA was taken from the GenBank acc. no. DQ376035.2. A whole genome alignment to the reference genome sequence from the strain CDC317 (GCA_000182765.2; [21,69]) was computed by Last version 830 [70] followed by last-split to assign to each portion of CDC317 sequence a unique position in our assembly. To annotate protein coding genes, the genes from the strain CDC317 were aligned to our assembly by Blat version v. 36x2 [71] and supplied as hints to Augustus version 3.2.3 [72]. Augustus was run originally with parameters for *Candida albicans*, then retrained on the predictions matching CDC317 genes. Disagreements with CDC317 annotation were manually inspected, and as a result, 61 genes were modified, 12 genes removed and 72 genes added.

### Electrophoretic karyotyping

About 1x10^9^ cells of the strain CLIB214 grown overnight in a YPD medium were resuspended in 0.5 ml of 1.2 M sorbitol, 40 mM citric acid, 120 mM disodium phosphate, 20 mM EDTA (pH 8.0), 5 mM DTT, 0.2 mg/ml zymolyase 20T (Seikagaku) and incubated for 90 min at 37°C. Protoplasts were then harvested by centrifugation (1 min, 2,100 *g*), resuspended in 1 ml of molten 1% [wt/vol] low melting point agarose in 50 mM EDTA (pH 8.5) cooled to 45°C and poured into the sample forms. The agarose embedded samples were incubated for 30 min at 37°C in 10 mM Tris.Cl, 0.5 M EDTA (pH 8.5) and then overnight at 50°C in 1% [wt/vol] N-lauroylsarcosine, 0.5 M EDTA (pH 8.5), 0.5 mg/ml proteinase K. Pulsed-field gel electrophoresis (PFGE) was performed in a CHEF Mapper XA Chiller System (Bio-Rad) with 120° angle between the electric fields at the following settings: (I) 120 s pulses for 24 h followed by 240 s pulses for 36 h at 4.5 V/cm; (II) 120 s pulses for 20 h followed by 240 s pulses for 28 h at 4 V/cm; (III): 60 s pulses for 15 hours followed by 90 s pulses for 9 hours at 6 V/cm. The samples were separated in 0.8% (settings I and II) or 1% [wt/vol] agarose gels (settings III) in 0.5x TBE buffer (45 mM Tris-borate, 1 mM EDTA, pH 8.0) at 14°C, throughout. After PFGE, the gels were stained with ethidium bromide (0.5 μg/ml) for 45 min and washed in water for additional 45 min.

### RNA-Seq analysis

*C*. *parapsilosis* CLIB214 was pre-cultivated in an SD medium (0.67% [wt/vol] yeast nitrogen base w/o amino acids (Difco), 2% [wt/vol] glucose) for 24 h at 28°C, then washed in water and re-grown in SMix10 medium (0.67% [wt/vol] yeast nitrogen base w/o amino acids (Difco), 3.3 mM 3-hydroxybenzoate, 3.3 mM 4-hydroxybenzoate, and 3.3 mM hydroquinone) for additional 24 h at 28°C. The pre-culture was inoculated (OD_600_ ~ 0.3) in triplicates into synthetic media (0.67% [wt/vol] yeast nitrogen base w/o amino acids (Difco)) containing 2% [wt/vol] galactose (SGal) or 10 mM hydroxyaromatic compound (i.e. 3-hydroxybenzoate (S3OH), 4-hydroxybenzoate (S4OH) or hydroquinone (SHyd)) as a sole carbon source and cultivated at 28°C till OD_600_ ~ 1. The consumption of hydroxyaromatic compounds in the cultivation media was analyzed spectrophotometrically (S14 Fig and S7 Table). The cultures of CPL2H1, *Δgtf1*/*Δgtf1*, and *Δotf1*/*Δotf1* were prepared similarly, except that the cultivation media were supplemented with leucine (20 μg/ml) and histidine (20 μg/ml) and the cells were grown in SMix15 medium (0.67% [wt/vol] yeast nitrogen base w/o amino acids (Difco), 5 mM 3-hydroxybenzoate, 5 mM 4-hydroxybenzoate, and 5 mM hydroquinone). Total RNA was isolated by extraction with hot acid phenol essentially as described in [73] and purified using an RNeasy mini kit (Qiagen) according to the manufacturer’s instructions. Transcriptome sequencing reads were generated from TruSeq stranded mRNA LT paired-end (2×151-nt) libraries on a NovaSeq6000 platform at Macrogen Korea. Reads were processed with Trimmomatic 0.39 [74] and mapped to the CLIB214 genome sequence using HiSat2 2.1.0 [75]. Duplicated reads were removed using samtools rmdup and the coverage was calculated using samtools depth (samtools version 1.9; [76]). Differential gene expression analysis was performed using the Geneious 11.1.5 package (Biomatters); the DESeq2 method [77] was used for samples in biological triplicates (*i*.*e*. CLIB214 grown in SGal, S3OH, S4OH, and SHyd media), the Geneious method was used for comparisons of CPL2H1 vs. *Δgtf1*/*Δgtf1* and CPL2H1 vs. *Δotf1*/*Δotf1* grown in SMix15 medium. Heatmaps were generated using the pheatmap package 1.0.12 (https://CRAN.R-project.org/package=pheatmap; [78]). Metabolic pathway and gene ontology (GO) enrichment analyses and the searches of Kyoto Encyclopedia of Genes and Genomes (KEGG) were performed using FungiDB (release 55; https://fungidb.org/; [79]) and the KEGG mapper (https://www.genome.jp/kegg/tool/map_pathway.html; [80]), respectively.

### Analyses of cultivation media

The consumption of hydroxyaromatic compounds and pH in cultivation media were analyzed before and at the end of cultivation using a Multiskan GO spectrophotometer (Thermo Scientific) and PH CHECK pH meter (Dostmann Electronic), respectively. The measurements were performed at room temperature. Following absorption maxima and media dilutions were used in the substrate consumption analyses; A_max_ = 297 nm, 2-fold dilution (3-hydroxybenzoate), A_max_ = 255 nm, 20-fold dilution (4-hydroxybenzoate), and A_max_ = 290 nm, 5-fold dilution (hydroquinone). To monitor pH changes during cultivations, synthetic media (see above) varying by the carbon source were supplemented with 0.01% [wt/vol] bromothymol blue and adjusted to pH 6.1–6.4 with NaOH. The cultures were inoculated to 6×10^6^ cells/ml and grown at 28°C to mid exponential phase (up to 60 hours). Cell-free media were used as a control. To document color, the cultures were centrifuged (1 min, 2,100 *g*) to remove the cells and 100 μl of cultivation media were transferred into wells of a 96-well plate and photographed using a Nikon D7000 camera.

### Colony morphology

Yeast cells were pre-grown in a complex medium (YPD) for 24 h at 28°C, washed with water, resuspended to ~ 10^7^ cells/ml and 40 μl aliquots were spotted onto Petri plates containing synthetic media differing by the carbon source (i.e. 2% glucose, 2% galactose or 10 mM hydroxyaromatic substrate). The plates were incubated for 30 days at 28°C.

### Proteomic analysis

Protein extracts were prepared in triplicates from *C*. *parapsilosis* CLIB214 cells pre-cultivated overnight at 28°C in an S3OH medium, inoculated (5×10^6^ cells/ml) to SGal, S3OH, S4OH, and SHyd media and grown at 28°C till ~ 10^7^ cells/ml. The cells were harvested by centrifugation (5 min, 2,100 *g* at 4°C), resuspended in 50 mM Tris-HCl (pH 8.8), 1 mM EDTA (pH 8.0) and homogenised using FastPrep-24 (MP Biomedicals). Cell debris was removed by centrifugation (15 min, 16,000 *g* at 4°C) and protein concentration was determined using Bradford`s method [81]. For LC-MS/MS analysis, protein aliquots (50 μg) were diluted in 100 μl of 25 mM Tris-HCl (pH 7.8), 0.1 mM CaCl_2_, treated using 5 mM dithiothreitol for 30 min at 60°C and alkylated in 40 mM chloroacetamide for 1 hour at 37°C. The proteins were digested overnight by trypsin (1:30 [wt/wt]) at 37°C. Acidified (0.5% [vol/vol] trifluoroacetic acid (TFA)) peptide solution was clarified by centrifugation and purified on a microtip C18 SPE. The concentration of eluted peptides was determined by Pierce Quantitative Fluorometric Peptide Assay (Thermo Scientific). The peptides were dissolved in 0.1% [vol/vol] TFA and 2% [vol/vol] acetonitrile (ACN), loaded (500 ng per run) onto a trap column (PepMap100 C18, 300 μm x 5 mm, Dionex, CA, USA) and separated with an EASY-Spray C18 column (75 μm x 500 mm, Thermo Scientific) on Ultimate 3000 RSLCnano system (Dionex) in a 120-minute gradient (3–43% B), curve 7, and flow-rate 250 nl/min. The two mobile phases were used: 0.1% [vol/vol] formic acid (A) and 80% [vol/vol] ACN with 0.1% [vol/vol] formic acid (B). Eluted peptides were sprayed directly into Orbitrap Elite mass spectrometer (Thermo Scientific, MA, USA) and spectral datasets were collected in the data dependent mode using Top15 strategy for the selection of precursor ions for the HCD fragmentation [82]. Each of the three experimental replicates was analysed in technical triplicates. Protein spectra were analyzed by MaxQuant software (version 1.6.17.0) using carbamidomethylation (C) as permanent and oxidation (M) and N-terminal acetylation as variable modifications, with engaged ‘match between the runs’ feature and label-free quantification (LFQ) and further examined in Perseus version 1.6.15.0 [83,84]. The search was performed against the *C*. *parapsilosis* CLIB214 protein database containing 5856 entries. Proteins were evaluated and annotated based on information from CDC317 strain orthologs. Contaminating peptides, reverse peptides and peptides only identified by site were removed, then the protein entries were further filtered to have at least two LFQ values in at least one of the biological conditions (different carbon sources). Following an imputation, differentially expressed proteins were identified by ANOVA test (permutation-based FDR 0.01).

### Catalase activity assay

*C*. *parapsilosis* CLIB214 cells were cultivated as described above for the RNA-Seq analysis. Cell extracts were prepared by homogenization of ~ 10^9^ cells resuspended in ice-cold 50 mM potassium phosphate buffer (pH 7.0) in a FastPrep 24 cell disrupter (MP Biomedicals) (3×20 s at a speed setting of 6.5 ms^−1^) using Lysis Matrix C (MP Biomedicals) and the lysates were centrifuged (10 min, 1,000 *g* at 4°C). Total catalase activity was determined by the decay rate of hydrogen peroxide monitored using an Evolution 350 UV-Vis Spectrophotometer (Thermo Scientific) at 240 nm (ɛ = 43.6 cm^−1^ mol^−1^ dm^3^) essentially as described previously [8,85]. One unit of catalase activity is defined as the amount of enzyme catalyzing the degradation of 1 mmol of hydrogen peroxide per minute at 25°C.

### Preparation of knockout strains

The mutants lacking either *GTF1*, *ICL1* or *OTF1* gene were generated in the strain CPL2H1 essentially as described in [62,86]. Deletion constructions contained the upstream (UpFw primer and UpRev primer; S8 Table) and downstream (DownFw primer and DownRev primer) homologous regions of the target ORF and either *Candida dubliniensis HIS1* or *Candida maltosa LEU2* sequences as selection markers. For selection marker amplification the primers ‘pSN52/pSN40 Fw’ and ‘pSN52/pSN40 Rev’ were used. DownFw, UpRev, and the primers used for marker amplification also harbored fusion sequences for later fragment joining. The reverse primer (‘pSN52/pSN40 Rev’) used for marker amplification also carried a TAG sequence between the mentioned fusion sequences. Deletion cassettes were transformed into CPL2H1 strain and the transformants were plated onto selective media. Heterozygous mutants were obtained and used to prepare homozygous mutants. Mutant strains were verified by colony polymerase chain reaction (PCR) using the primers specific for both the marker sequences and the outside of the integration sites at both the upstream and downstream homologous regions. The ORF specific primer ‘5’- check primer’ was used as forward primer together with ‘*LEU1/HIS1 primer’* as reverse primer, while the ORF specific primer ‘3’- check primer’ was applied as reverse primer together with the ‘*LEU2/HIS2* primer’ as forward primer.

Assimilation tests of the wild type (CLIB214) and mutant strains were performed on solid synthetic media (0.67% [wt/vol] yeast nitrogen base w/o amino acids (Difco), 2% [wt/vol] agar) differing by the carbon source (i.e. 2% [wt/vol] glucose (SD), 10 mM 3-hydroxybenzoate (S3OH), 10 mM 4-hydroxybenzoate (S4OH), 10 mM 2,4-dihydroxybenzoate (S24diOH), 10 mM 2,5-dihydroxybenzoate (S25diOH), 10 mM 3,4-dihydroxybenzoate (S34diOH), 10 mM hydroquinone (SHyd) or 10 mM resorcinol (SRes)). Prior to the addition to the media, hydroxyaromatic compounds were dissolved in dimethyl sulfoxide (DMSO) as 0.5 M stocks.

### Fluorescence microscopy

To visualize peroxisomes in *C*. *parapsilosis* cells, we constructed a plasmid pBP7-mCherry-SKL expressing the mCherry protein tagged with peroxisomal targeting signal ‘SKL’ at its C-terminus and a control plasmid pBP7-mCherry expressing the unmodified protein. The mCherry coding sequence was amplified by PCR using the primers shown in S8 Table and the plasmid pMG2254 [42] as a template. The PCR products were inserted into the *Xba*I site of the pBP7 vector [87] using a Gibson assembly cloning kit (New England Biolabs). The cloned genes are placed downstream of the *GAL1* promoter in the resulting plasmid constructs. The constructs were transformed into *C*. *parapsilosis* cells CDU1 by the standard protocol [88]. The transformants were cultivated for 48 hours in liquid synthetic medium (0.67% [wt/vol] yeast nitrogen base w/o amino acids (Difco)) containing 2% [wt/vol] glucose (SD) at 28°C. The cells were then washed with water and inoculated to synthetic media differing by the carbon source (i.e. 2% [wt/vol] galactose (SGal), 10 mM 3-hydroxybenzoate (S3OH), 10 mM 4-hydroxybenzoate (S4OH), 10 mM hydroquinone (SHyd)), cultivated for 24 (SGal) or 48 hours (S3OH, S4OH, SHyd) at 28°C and examined by fluorescence microscopy using Olympus BX61 microscope with filter set U-MWIG3 and a digital camera Olympus XM10. The obtained images were colorized using Fiji (version 2.1.0/1.53c) [89]. To investigate the intracellular localization of Gtf1p and Otf1p, we constructed yEGFP3-tagged versions of both proteins as follows. The coding sequences of *GTF1* and *OTF1* were PCR-amplified from the CLIB214 genomic DNA using gene specific primers (S8 Table) and the PCR products were inserted into the *Sma*I site of the pPK6 vector [87] using a Gibson assembly cloning kit (New England Biolabs). This allows the expression of cloned genes under the control of the *GAL1* promoter. The plasmid constructs were transformed into *C*. *parapsilosis* cells SR23 *met-1* as described in [88]. The transformants were grown overnight in an SD medium, washed with water, inoculated into an SGal medium, and cultivated overnight at 28°C. Prior to fluorescent microscopy, the cellular DNA was stained with 4′,6-diamidino-2-phenylindole (DAPI, 1 μg/ml) for 20 min. The cells were observed using Zeiss Axio Imager.Z2 microscope (objective "Plan-Apochromat" 100x) with filter sets 38 HE (for GFP) and 49 (for DAPI) and a digital camera Zeiss Axiocam 506 mono. The obtained images were colorized using Fiji (version 2.1.0/1.53c) [89].

### Electrophoretic Mobility Shift Assay (EMSA)

The wild type (CPL2H1) and mutant (*Δgtf1/Δgtf1*, *Δotf1/Δotf1*) cells were grown in synthetic media containing combinations of hydroxyaromatic substrates (i.e. 7.5 mM 3-hydroxybenzoate and 7.5 mM 4-hydroxybenzoate (wild type); 2.5 mM 3-hydroxybenzoate, 2.5 mM 4-hydroxybenzoate, and 10 mM hydroquinone (*Δgtf1/Δgtf1*); 10 mM 3-hydroxybenzoate and 5 mM 4-hydroxybenzoate (*Δotf1/Δotf1*)). The medium for cultivation of the wild type strain was supplemented with leucine (40 μg/ml) and histidine (40 μg/ml). Protein extracts were prepared according to Winkler *et al*. [90] with some modifications. Ice-cold solutions were used throughout the experiment and all incubations were performed on ice. Cells were harvested at exponential growth phase by centrifugation (10 min, 3,600 *g* at 4°C), washed with water, resuspended in 5 volumes of 200 mM Tris-HCl (pH 8.0), 400 mM (NH_4_)_2_SO_4_, 10 mM MgCl_2_, 1 mM EDTA, 7 mM 2-mercaptoethanoI, 10% [vol/vol] glycerol, 1 mM phenylmethylsulfonyl fluoride (PMSF), 1 × cOmplete protease inhibitor cocktail tablet (Roche Applied Science). The cells were disrupted by vortexing with glass-beads (0.45–0.5 mm in diameter, 0.8 g/ml) 7 times for 1 min with intermittent cooling on ice for 1 min. Lysates were incubated for 30 min, centrifuged at 9,000 *g* for 60 min and proteins in supernatant were precipitated by addition of (NH_4_)_2_SO_4_ in 10 mM HEΡΕS (pH 8.0), 5 mM EDTA, 1 mM PMSF for 30 min (the final concentration of (NH_4_)_2_SO_4_ was 40% [wt/vol] in total volume of 1.5 ml). The sample was centrifuged at 9,000 *g* for 15 min and the pellet was resuspended in 100–150 μl of 10 mM HEΡΕS (pH 8.0), 5 mM EDTA, 7 mM 2-mercaptoethanol, 20% [vol/vol] glycerol, 1 mM PMSF, 1× cOmplete protease inhibitor cocktail tablet (Roche Applied Science). The protein extracts were stored at -80°C prior to the use in DNA-binding assays. Oligonucleotide probes were prepared as follows. Direct strand oligonucleotides (S8 Table) were labeled at 5′ end by T4 polynucleotide kinase (Thermo Scientific) and [γ-^32^P]ATP (Hartmann Analytic), mixed with 3-fold molar excess of the unlabeled complementary oligonucleotide, heated at 100°C for 10 min and slowly cooled down to room temperature to allow efficient formation of the double-stranded probes. The probes were purified using Illustra MicroSpin G-25 Columns (GE Healthcare). The DNA binding assays were carried out in 10 μl of 10 mM Tris-HCl (pH 7.5), 50 mM NaCl, 0.1 mM EDTA containing 15 μg of proteins, 2 ng of the ^32^P-labeled probe, 2 μg of poly(dA-dC) • poly(dG-dT). Unlabeled double-stranded oligonucleotides were used as specific competitors. The reaction mixtures were incubated for 15 min at room temperature and immediately loaded on 5% polyacrylamide gels in TG buffer (25 mM Tris-HCl (pH 8.3), 192 mM glycine). The electrophoresis was performed at 4°C in the TG buffer at 10 V/cm for 90 min. Gels were fixed with 10 ml of 10% [vol/vol] methanol, 10% [vol/vol] acetic acid for 10 min, dried and exposed to the storage phosphor screen. Signal detection was performed using a Personal Molecular Imager FX (Bio-Rad) or Amersham Typhoon Biomolecular Imager (GE Healthcare Bio-Sciences AB).

### Phylogenetic analysis

Sequences were aligned with Muscle v3.8 [91] with default parameters and maximum likelihood phylogenetic trees were built using IQ-Tree v2.0 [60] allowing full exploration of model parameters and estimating the support of tree partitions using ultrafast bootstrap support with 1000 iterations [92]. Orthology and paralogy relationships, as well as duplication nodes were inferred with the species overlap algorithm [93], with the relative age inferred from topological analysis [94]. Blast searches were performed at NCBI website (https://blast.ncbi.nlm.nih.gov/) using default parameters unless indicated otherwise. Phylogenetic analysis of the *C*. *parapsilosis* amidohydrolase gene was done as follows: EggNOG-mapper v2 was used through the web server at http://eggnog-mapper.embl.de/ with default parameters. Due to an error in the FASTA file of COG2159, which contained only 6738 bacterial sequences but missed the eukaryotic and archaeal genes, we added the two homologous archeal (arCOG01931, 198 sequences) and eukaryotic (KOG4245, 613 sequences) orthologous groups, which are contained within COG2159. These sequences were aligned with MAFFT [95] with the auto option enabled. The resulting alignment was cleaned with TrimAl [96] with the gappyout method and the phylogenetic tree was then computed with FastTree 2 [59]. The subset of highly supported closest homologous sequences to the amidohydrolase gene was manually selected and realigned with the same strategy. The phylogenetic tree this time was computed with IQ-Tree [60] with ModelFinder [97] finding LG+F+R8 as the best model.

## Supporting information

S1 Table RNA-Seq analysis of *C*. *parapsilosis* cells. Lists of genes differentially expressed in *C*. *parapsilosis* CLIB214 cells grown in synthetic media containing 3-hydroxybenzoate, 4-hydroxybenzoate or hydroquinone compared to the cells assimilating galactose (i.e. S3OH vs. SGal, S4OH vs. SGal, SHyd vs. SGal) or 4-hydroxybenzoate (S3OH vs. S4OH, SHyd vs. S4OH). The estimate of fold expression change was computed by DESeq2 [77] based on three replicates of each growth condition. DESeq2 uses a generalized linear model and empirical Bayes shrinking to estimate log_2_ fold expression change and the standard error of this estimate; the p-value of a gene being differentially expressed is then obtained using a Wald test based on the z-score of the log_2_ fold change as the test statistics (multiple testing correction by false discovery rate method). The base mean is the average of count values over all experiments, normalized by library size factors.
(XLSX)

S2 Table RNA-Seq analysis of *C*. *parapsilosis* mutants lacking Otf1p or Gtf1p. Lists of genes differentially expressed in the *C*. *parapsilosis* mutants *Δgtf1/Δgtf1* and *Δotf1/Δotf1* compared to the parental strain CPL2H1 (i.e. *Δgtf1/Δgtf1* vs. CPL2H1 and *Δotf1/Δotf1* vs. CPL2H1). The cells were grown in synthetic media (SMix15) containing three hydroxyaromatic carbon sources (i.e. 3-hydroxybenzoate, 4-hydroxybenzoate, and hydroquinone).
(XLSX)

S3 Table LC-MS/MS analysis of proteins extracted from *C*. *parapsilosis* cells. Lists of proteins identified in the extracts of *C*. *parapsilosis* CLIB214 cells grown in synthetic media containing 3-hydroxybenzoate (S3OH), 4-hydroxybenzoate (S4OH), hydroquinone (SHyd) or galactose (SGal) as a carbon source. Protein spectra were subjected to label-free quantification (LFQ) and statistically evaluated.
(XLSX)

S4 Table Metabolic pathways enrichment analysis. The analysis was performed using FungiDB (release 55; https://fungidb.org/; [79]). *Candida parapsilosis* CDC317 and KEGG were used as an organism and pathways source in settings. Note that only the genes highly upregulated (log_2_ fold change ≥ 2) on indicated synthetic media with 3-hydroxybenzoate, 4-hydroxybenzoate or hydroquinone compared to galactose-containing medium (i.e. S3OH vs. SGal, S4OH vs. SGal, SHyd vs. SGal) were included in the analysis.
(XLSX)

S5 Table Catalase activity in cells assimilating hydroxyaromatic substrates. The dataset used for preparing the bar graph shown in S3 Fig.
(XLSX)

S6 Table Gene ontology (GO) enrichment analysis. The analysis was performed using FungiDB (release 55; https://fungidb.org/; [79]). *Candida parapsilosis* CDC317 was used as an organism in settings. Note that only the genes highly upregulated (log_2_ fold change ≥ 2) on indicated synthetic media with 3-hydroxybenzoate, 4-hydroxybenzoate or hydroquinone compared to galactose-containing medium (i.e. S3OH vs. SGal, S4OH vs. SGal, SHyd vs. SGal) were included in the analysis.
(XLSX)

S7 Table Spectrophotometric analysis of hydroxyaromatic substrate consumption in cultivation media. The datasets used for preparing the plots shown in S14 Fig.
(XLSX)

S8 Table List of synthetic oligonucleotides. (XLSX)

S1 Text Phylogenetic tree of GTF1 sequences in the Newick format. (TXT)

S2 Text Phylogenetic tree of OTF1 sequences in the Newick format. (TXT)

S1 Fig Homologs of amidohydrolase family proteins. Amino acid sequence alignment of conceptual translation of *C*. *parapsilosis* CANPARB_p44920-A (red shading), short intergenic spacer, and CANPARB_p44910-A (blue shading) with yeast (*C*. *metapsilosis* (g2237), *T*. *ciferrii* (KAA8915622.1), *W*. *sorbophila* (XP_024665283.1), *N*. *castellii* (XP_003673849.1)) and bacterial (*Pseudomonas aestus* (P308_18355), *Paraburkholderia megapolitana* (SAMN05192543_101920), and *Variovorax* sp. (VAR608DRAFT_1163)) homologs. The alignment was calculated using MAFFT [95].
(TIF)

S2 Fig Expression profiles of *C*. *parapsilosis* genes coding for metabolic enzymes. The heatmaps show the expression profiles obtained by the RNA-Seq and LC-MS/MS analyses. The log_2_ fold change values obtained by the RNA-Seq analysis (S1 Table) are shown on the left panel. Only the genes that are upregulated (log_2_ fold change ≥ 2; adjusted p-value ≤ 0.05) on at least one hydroxyaromatic substrate and code for protein products classified as metabolic enzymes (based on the searches using the BlastKOALA (https://www.kegg.jp/blastkoala/; [98]) and KEGG Mapper tools (https://www.kegg.jp/kegg/tool/map_pathway.html; [80]) are included. Note that the values that are not statistically significant (i.e. adjusted p-value > 0.05) are shown in parentheses. The values on the right panel represent log_2_ of mean LFQ intensity ratios taken from the LC-MS/MS analysis (S3 Table). Note that the LFQ values imputed from a normal distribution were used for proteins that were not identified on all carbon sources (shown in parentheses). Proteins CANPARB_p24940-A and CANPARB_p24960-A, and similarly also CANPARB_p56420-A and CANPARB_p56500-A, have almost identical sequences and therefore could not be distinguished by the LC-MS/MS analysis. Orthologs or best hits (indicated by an asterisk) from the *C*. *parapsilosis* reference strain CDC317, *C*. *albicans*, and *S*. *cerevisiae*, and the KEGG IDs are indicated.
(PDF)

S3 Fig Catalase activity in *C*. *parapsilosis* cells assimilating hydroxyaromatic substrates. *C*. *parapsilosis* CLIB214 cells were grown in synthetic minimal media containing galactose, 3-hydroxybenzoate, 4-hydroxybenzoate or hydroquinone as a carbon source. Catalase activity was measured spectrophotometrically in cell lysates (see Materials and methods for details). The assays were performed in three independent experiments with three parallel measurements in each case, the bar graph shows the mean value ± standard deviation (S5 Table). The significance of differences between the samples (3-hydroxybenzoate, 4-hydroxybenzoate, hydroquinone) and the control (galactose) was evaluated by Student’s t-test (* P < 0.05; **** P < 0.0001). Note that the *C*. *parapsilosis* genome encodes three catalase homologs (i.e. CANPARB_p07760−A/CPAR2_207780, CANPARB_p28470-A/CPAR2_803840, and CANPARB_p28480−A/CPAR2_803850).
(TIF)

S4 Fig Acyl-CoA synthetases in *C*. *parapsilosis*. (A) The heatmaps show the expression profiles of *C*. *parapsilosis FAA* genes. The log_2_ fold change values obtained by the RNA-Seq analysis (S1 Table) are shown on the left panel. Note that the values that are not statistically significant (i.e. p > 0.05) are shown in parentheses. The values on the right panel represent log_2_ of mean LFQ intensity ratios taken from the LC-MS/MS analysis (S3 Table). (B) Phylogenetic relationships of *C*. *parapsilosis FAA* genes and their homologs in other yeasts. The *CPAR2_200640* gene tree in phylome 498 from PhylomeDB (*Candida inconspicua* genome, described in [99]) was used as a template to create this figure, which is only shown partially here. Sequences from *C*. *parapsilosis* (black), *C*. *albicans* (red), and *S*. *cerevisiae* (blue) are highlighted with their names. Shadowed rectangles around them indicate, respectively, the spread of species from the *C*. *parapsilosis sensu lato*, *C*. *albicans / C*. *dubliniensis / C*. *tropicalis* clade, and *Saccharomyces / Nakaseomyces* clade. Colored circles indicate duplication nodes, with different colors indicating the relative age inferred from this duplication (see legend).
(PDF)

S5 Fig The *C*. *parapsilosis Δicl1*/*Δicl1* mutant shows impaired growth on substrates of the 3-oxoadipate pathway. *C*. *parapsilosis* CLIB214 (wild type) and *Δicl1*/*Δicl1* mutant cells were pre-grown overnight in a complex medium (YPD) at 28°C, washed with water and resuspended to ~ 6×10^6^ cells/ml. Serial fivefold dilutions were then spotted on solid synthetic media containing indicated carbon sources. The plates were incubated for 5 days at 28°C.
(TIF)

S6 Fig Expression profiles of *C*. *parapsilosis* genes involved in the biogenesis and metabolism of peroxisomes. The heatmaps show the expression profiles obtained from the RNA-Seq and LC-MS/MS analyses. The log_2_ fold change values obtained by the RNA-Seq analysis (S1 Table) are shown on the left panel. Only the genes that are upregulated (log_2_ fold change ≥ 2; adjusted p-value ≤ 0.05) on at least one hydroxyaromatic substrate and code for protein products classified into categories ‘peroxisome’, ‘peroxisomal matrix’, ‘peroxisomal membrane’ or ‘peroxisomal importomer complex’ (based on the GO enrichment analysis; S6 Table) are included. Note that the values that are not statistically significant (i.e. adjusted p-value > 0.05) are shown in parentheses. The values on the right panel represent log_2_ of mean LFQ intensity ratios taken from the LC-MS/MS analysis (S3 Table). Orthologs or best hits (indicated by an asterisk) from the *C*. *parapsilosis* reference strain CDC317, *C*. *albicans*, and *S*. *cerevisiae* are shown.
(PDF)

S7 Fig Amino acid sequence alignments of Otf1p and Gtf1p orthologs. (A) Amino acid sequence alignment of *C*. *parapsilosis* Otf1p with the counterparts from *C*. *metapsilosis* (CMET_1974), *C*. *orthopsilosis* (CORT0C05870), *C*. *albicans* (ZCF10), *C*. *tropicalis* (CTRG_01734), *Scheffersomyces stipitis* (PICST_62477), and *Spathaspora passalidarum* (SPAPADRAFT_137814). (B) Amino acid sequence alignment of *C*. *parapsilosis* Gtf1p with the counterparts from *C*. *metapsilosis* (CMET_1081), *S*. *passalidarum* (SPAPADRAFT_53773), *Debaryomyces hansenii* (DEHA2C00946g), and *S*. *stipitis* (PICST_57167 and PICST_65252). The alignments were calculated using MAFFT [95]. The GAL4-like domain (red shading) and fungal specific transcription factor domain (blue shading) were predicted using SMART 8.0 [100]. Nuclear localisation signal (NLS, shown in magenta) was identified using SeqNLS [101].
(TIF)

S8 Fig Downregulated genes in *C*. *parapsilosis* mutants lacking Otf1p or Gtf1p. The heatmap shows the genes downregulated (log_2_ fold change ≤ -2; adjusted p-value ≤ 0.05; S2 Table) in the mutants *Δgtf1/Δgtf1* and *Δotf1/Δotf1* compared to the parental strain CPL2H1 (*Δgtf1/Δgtf1* vs. CPL2H1 and *Δotf1/Δotf1* vs. CPL2H1). The cells were grown in an SMix15 medium containing three hydroxyaromatic carbon sources (i.e. 3-hydroxybenzoate, 4-hydroxybenzoate, and hydroquinone). Note that the values that are not statistically significant (i.e. adjusted p-value > 0.05) are shown in parentheses. Orthologs or best hits (indicated by an asterisk) from *C*. *parapsilosis* CDC317, *C*. *albicans*, and *S*. *cerevisiae*, and KEGG IDs are indicated.
(PDF)

S9 Fig Putative binding sites for Otf1p and Gtf1p in the promoters of 3-OAP and GP genes. The occurrence of putative Otf1p (A) and Gtf1p (B) binding sites in the upstream regions (+3 to -1000) of the genes encoding the components of the 3-OAP and GP, respectively. The sequences arranged in the 3-OAP or GP gene clusters are indicated in bold. Putative Mig1p-binding sites and the positions of probes used in the EMSA experiments are also depicted.
(TIF)

S10 Fig Predicted Otf1p and Gtf1p binding motifs. (A) Otf1p binding motif. (B) Gtf1p binding motif. The sequence logos were derived from predicted binding sites identified in the promoter sequences shown in S9 Fig. The sequences arranged in the 3-OAP or GP gene clusters are indicated in bold. Note that the Otf1p binding motif is asymmetrical and only the sites oriented toward the corresponding coding sequence (shown in blue in S9 Fig) were used in the alignment.
(TIF)

S11 Fig Transcription factors Otf1p and Gtf1p bind to predicted sequence motifs. The EMSA experiments were performed using the protein extracts (i.e. ~ 15 μg) prepared from CPL2H1 (A),(C), *Δotf1/Δotf1* (B), and *Δgtf1/Δgtf1* (D) cells and the 5’ end-labeled dsDNA probes containing the predicted Otf1p-binding site from the *MNX1* promoter (OTF1_MNX1; (A),(B)) or the Gtf1p-binding site from the *MNX2* promoter (GTF1_MNX2; (C),(D)). The ds oligonucleotide competitors containing either the wild type (OTF1_MNX1, GTF1_MNX2) or mutated binding motifs (OTF1_MNX1_mut, GTF1_MNX2_mut) were used with increasing amounts of 100, 300, and 500 ng as indicated above lanes. The equivalent aliquots of protein extracts from CPL2H1 (E), *Δotf1/Δotf1* (F), and *Δgtf1/Δgtf1* (G) cells were also examined by SDS-PAGE and stained with PageBlue Protein Staining Solution (Thermo Scientific). Spectra Multicolor Broad Range Protein Ladder (Thermo Scientific; 10 μl) was used as a molecular weight standard. The gels were photographed using a GelDoc-It^2^ Imager (UVP) and the images were processed by VisionWorks Acquisition and Analysis Software (Analytik Jena). The gels are shown in Coomassie Blue pseudocolor.
(TIF)

S12 Fig Maximum Likelihood phylogenetic trees of EggNOG fungal orthogroups for OTF1 and GTF1. (TIF)

S13 Fig Phylogenetic analysis of the *C*. *parapsilosis* amidohydrolase gene. Maximum Likelihood phylogenetic tree of the subset of the 250 closest homologs to the *C*. *parapsilosis* amidohydrolase gene (marked as Amidohydro_CANPA). The tree was rooted at midpoint and monophyletic nodes representing species from the same phylum were collapsed and color coded.
(TIF)

S14 Fig Consumption of hydroxyaromatic substrates by *C*. *parapsilosis* cells. *C*. *parapsilosis* CLIB214 cells grown in the synthetic media containing a hydroxyaromatic substrate as a sole carbon source at 28°C till OD_600_ ~ 1. Substrate consumption was inferred from the absorption spectra (200–350 nm) measured in the media of three parallel cultures (shown in red, blue, and green) after cultivation (t = 17.5, 25.5, and 16 hours for 3-hydroxybenzoate, 4-hydroxybenzoate, and hydroquinone, respectively) as well as in the control medium. Each measurement was performed in three technical replicates. The samples were diluted 2-, 20-, and 5-fold prior analysis of 3-hydroxybenzoate, 4-hydroxybenzoate, and hydroquinone consumption, respectively. The dataset for each panel is shown in S7 Table.
(TIF)

## References

[1] Slot JC. Fungal gene cluster diversity and evolution. Adv Genet. 2017;100:141–78. doi: 10.1016/bs.adgen.2017.09.005 29153399

[2] Nützmann HW, Scazzocchio C, Osbourn A. Metabolic gene clusters in eukaryotes. Annu Rev Genet. 2018;52:159–83. doi: 10.1146/annurev-genet-120417-031237 30183405

[3] Vesth TC, Brandl J, Andersen MR. FunGeneClusterS: Predicting fungal gene clusters from genome and transcriptome data. Synth Syst Biotechnol. 2016;1:122–9. doi: 10.1016/j.synbio.2016.01.002 29062935 PMC5640693

[4] Töpfer N, Fuchs LM, Aharon A. The PhytoClust tool for metabolic gene clusters discovery in plant genomes. Nucleic Acids Res. 2017;45:7049–63. doi: 10.1093/nar/gkx404 28486689 PMC5499548

[5] Marcet-Houben M, Gabaldón T. Evolutionary and functional patterns of shared gene neighbourhood in fungi. Nat Microbiol. 2019;4:2383–92. doi: 10.1038/s41564-019-0552-0 31527797

[6] Wisecaver JH, Rokas A. Fungal metabolic gene clusters-caravans traveling across genomes and environments. Front Microbiol. 2015;6:161. doi: 10.3389/fmicb.2015.00161 25784900 PMC4347624

[7] Holešová Z, Jakúbková M, Zavadiaková I, Zeman I, Tomáška Ľ, Nosek J. Gentisate and 3-oxoadipate pathways in the yeast *Candida parapsilosis*: identification and functional analysis of the genes coding for 3-hydroxybenzoate 6-hydroxylase and 4-hydroxybenzoate 1-hydroxylase. Microbiology (Reading). 2011;157:2152–63. doi: 10.1099/mic.0.048215-0 21474535

[8] Gérecová G, Neboháčová M, Zeman I, Pryszcz LP, Tomáška Ľ, Gabaldón T, et al. Metabolic gene clusters encoding the enzymes of two branches of the 3-oxoadipate pathway in the pathogenic yeast *Candida albicans*. FEMS Yeast Res. 2015;15:fov006. doi: 10.1093/femsyr/fov006 25743787

[9] Zeman I, Neboháčová M, Gérecová G, Katonová K, Jánošíková E, Jakúbková M, et al. Mitochondrial carriers link the catabolism of hydroxyaromatic compounds to the central metabolism in *Candida parapsilosis*. G3 (Bethesda). 2016;6:4047–58. doi: 10.1534/g3.116.034389 27707801 PMC5144973

[10] Cillingová A, Zeman I, Tóth R, Neboháčová M, Dunčková I, Hölcová M, et al. Eukaryotic transporters for hydroxyderivatives of benzoic acid. Sci Rep. 2017;7:8998. doi: 10.1038/s41598-017-09408-6 28827635 PMC5566891

[11] Vrzoňová R, Tóth R, Siváková B, Moťovská A, Gaplovská K, Baráth P, et al. OCT1 – a yeast mitochondrial thiolase involved in the 3-oxoadipate pathway. FEMS Yeast Res. 2021;21: foab034. doi: 10.1093/femsyr/foab034 34089318

[12] Mixão V, Hegedűsová E, Saus E, Pryszcz LP, Cillingová A, Nosek J, et al. Genome assembly of *Candida subhashii* reveals its hybrid nature and dual mitochondrial genome conformation. DNA Res. 2021;28:dsab006. doi: 10.1093/dnares/dsab006 34129020 PMC8311171

[13] Middelhoven WJ, Coenen A, Kraakman B, Sollewijn Gelpke MD. Degradation of some phenols and hydroxybenzoates by the imperfect ascomycetous yeasts *Candida parapsilosis* and *Arxula adeninivorans*: evidence for an operative gentisate pathway. Antonie van Leeuwenhoek 1992;62:181–7. doi: 10.1007/BF00582578 1416914

[14] Eppink MH, Cammaart E, Van Wassenaar D, Middelhoven WJ, van Berkel WJ. Purification and properties of hydroquinone hydroxylase, a FAD-dependent monooxygenase involved in the catabolism of 4-hydroxybenzoate in *Candida parapsilosis* CBS604. Eur J Biochem. 2000;267:6832–40. doi: 10.1046/j.1432-1033.2000.01783.x 11082194

[15] Csonka K, Vadovics M, Marton A, Vágvölgyi C, Zajta E, Tóth A, et al. Investigation of OCH1 in the virulence of *Candida parapsilosis* using a new neonatal mouse model. Front Microbiol. 2017;8:1197. doi: 10.3389/fmicb.2017.01197 28713338 PMC5491538

[16] Turner SA, Ma Q, Ola M, Martinez de San Vicente K, Butler G. Dal81 regulates expression of arginine metabolism genes in *Candida parapsilosis*. mSphere 2018;3:e00028–18. doi: 10.1128/mSphere.00028-18 29564399 PMC5853489

[17] Nguyen TN, Dubreucq E, Perrier V, Tran QH, Charpentier C, Charnay C, et al. Interactions between *trans*-resveratrol and *Cp*LIP2 lipase/acyltransferase: Evidenced by fluorescence and *in silico*. Food Chem. 2020;318:126482. doi: 10.1016/j.foodchem.2020.126482 32145543

[18] Pál SE, Tóth R, Nosanchuk JD, Vágvölgyi C, Németh T, Gácser A. A *Candida parapsilosis* overexpression collection reveals genes required for pathogenesis. J Fungi (Basel) 2021;7:97. doi: 10.3390/jof7020097 33572958 PMC7911391

[19] Ashford B. Certain conditions of the gastrointestinal tract in Puerto Rico and their relation to tropical sprue. Am J Trop Med Hyg. 1928;8:507–38.

[20] Logue ME, Wong S, Wolfe KH, Butler G. A genome sequence survey shows that the pathogenic yeast *Candida parapsilosis* has a defective MTLa1 allele at its mating type locus. Eukaryot Cell 2005;4:1009–17. doi: 10.1128/EC.4.6.1009-1017.2005 15947193 PMC1151992

[21] Butler G, Rasmussen MD, Lin MF, Santos MAS, Sakthikumar S, Munro CA, et al. Evolution of pathogenicity and sexual reproduction in eight *Candida* genomes. Nature 2009;459:657–62. doi: 10.1038/nature08064 19465905 PMC2834264

[22] Gluck-Thaler E, Vijayakumar V, Slot JC. Fungal adaptation to plant defences through convergent assembly of metabolic modules. Mol Ecol. 2018;27:5120–36. doi: 10.1111/mec.14943 30427102

[23] Greene GH, McGary KL, Rokas A, Slot JC. Ecology drives the distribution of specialized tyrosine metabolism modules in fungi. Genome Biol Evol. 2014;6:121–32. doi: 10.1093/gbe/evt208 24391152 PMC3914699

[24] Martins TM, Martins C, Guedes P, Silva Pereira C. Twists and turns in the salicylate catabolism of *Aspergillus terreus*, revealing new roles of the 3-hydroxyanthranilate pathway. mSystems 2021;6:e00230–20. doi: 10.1128/mSystems.00230-20 33500329 PMC7842363

[25] Van Roermund CWT, Waterham HR, Ijlst L, Wanders RJA. Fatty acid metabolism in *Saccharomyces cerevisiae*. Cell Mol Life Sci. 2003;60:1838–51. doi: 10.1007/s00018-003-3076-x 14523547 PMC11138760

[26] Hashimoto F, Hayashi H. Significance of catalase in peroxisomal fatty acyl-CoA beta-oxidation: NADH oxidation by acetoacetyl-CoA and H_2_O_2_. J Biochem. 1990;108:426–31. doi: 10.1093/oxfordjournals.jbchem.a123217 2277034

[27] Black PN, DiRusso CC. Yeast acyl-CoA synthetases at the crossroads of fatty acid metabolism and regulation. Biochim Biophys Acta – Mol Cell Biol Lipids 2007;1771:286–98. doi: 10.1016/j.bbalip.2006.05.003 16798075

[28] Kunze M, Pracharoenwattana I, Smith SM, Hartig A. A central role for the peroxisomal membrane in glyoxylate cycle function. Biochim Biophys Acta – Mol Cell Res. 2006;1763:1441–52. doi: 10.1016/j.bbamcr.2006.09.009 17055076

[29] Cartwright JL, Gasmi L, Spiller DG, McLennan AG. The *Saccharomyces cerevisiae PCD1* gene encodes a peroxisomal nudix hydrolase active toward coenzyme A and its derivatives. J Biol Chem. 2000;275:32925–30. doi: 10.1074/jbc.M005015200 10922370

[30] Strijbis K, Distel B. Intracellular acetyl unit transport in fungal carbon metabolism. Eukaryot Cell. 2010;9:1809–15. doi: 10.1128/EC.00172-10 20889721 PMC3008284

[31] Beyer A, Hollunder J, Nasheuer HP, Wilhelm T. Post-transcriptional expression regulation in the yeast *Saccharomyces cerevisiae* on a genomic scale. Mol Cell Proteomics. 2004;3:1083–92. doi: 10.1074/mcp.M400099-MCP200 15326222

[32] Wu G, Nie L, Zhang W. Integrative analyses of posttranscriptional regulation in the yeast *Saccharomyces cerevisiae* using transcriptomic and proteomic data. Curr Microbiol. 2008;57:18–22. doi: 10.1007/s00284-008-9145-5 18363056

[33] Pronk JT, Yde Steensma H, Van Dijken JP. Pyruvate metabolism in *Saccharomyces cerevisiae*. Yeast 1996;12:1607–33. doi: 10.1002/(sici)1097-0061(199612)12:16&lt;1607::aid-yea70&gt;3.0.co;2-4 9123965

[34] Carman AJ, Vylkova S, Lorenz MC. Role of acetyl coenzyme A synthesis and breakdown in alternative carbon source utilization in *Candida albicans*. Eukaryot Cell 2008;7:1733–41. doi: 10.1128/EC.00253-08 18689527 PMC2568070

[35] Otzen C, Bardl B, Jacobsen ID, Nett M, Brock M. *Candida albicans* utilizes a modified β-oxidation pathway for the degradation of toxic propionyl-CoA. J Biol Chem. 2014;289:8151–69. doi: 10.1074/jbc.M113.517672 24497638 PMC3961645

[36] Saraya R, Veenhuis M, van der Klei IJ. Peroxisomes as dynamic organelles: peroxisome abundance in yeast. FEBS J. 2010;277:3279–88. doi: 10.1111/j.1742-4658.2010.07740.x 20629743

[37] Thoms S, Erdmann R. Dynamin-related proteins and Pex11 proteins in peroxisome division and proliferation. FEBS J. 2005;272:5169–81. doi: 10.1111/j.1742-4658.2005.04939.x 16218949

[38] Koch J, Brocard C. Membrane elongation factors in organelle maintenance: the case of peroxisome proliferation. Biomol Concepts 2011;2:353–64. doi: 10.1515/BMC.2011.031 21984887 PMC3186938

[39] Jansen RLM, van der Klei IJ. The peroxisome biogenesis factors Pex3 and Pex19: multitasking proteins with disputed functions. FEBS Lett. 2019;593:457–74. doi: 10.1002/1873-3468.13340 30776093

[40] Walter T, Erdmann R. Current advances in protein import into peroxisomes. Protein J. 2019; 38:351–62. doi: 10.1007/s10930-019-09835-6 31054036

[41] Knoblach B, Rachubinski RA. How peroxisomes partition between cells. A story of yeast, mammals and filamentous fungi. Curr Opin Cell Biol. 2016;41:73–80. doi: 10.1016/j.ceb.2016.04.004 27128775

[42] Gerami-Nejad M, Dulmage K, Berman J. Additional cassettes for epitope and fluorescent fusion proteins in *Candida albicans*. Yeast 2009;26:399–406. doi: 10.1002/yea.1674 19504625 PMC3086567

[43] van Berkel WJ, Eppink MH, Middelhoven WJ, Vervoort J, Rietjens IM. Catabolism of 4-hydroxybenzoate in *Candida parapsilosis* proceeds through initial oxidative decarboxylation by a FAD-dependent 4-hydroxybenzoate 1-hydroxylase. FEMS Microbiol Lett. 1994;121:207–15. doi: 10.1111/j.1574-6968.1994.tb07100.x 7926672

[44] Eppink MH, Boeren SA, Vervoort J, van Berkel WJ. Purification and properties of 4-hydroxybenzoate 1-hydroxylase (decarboxylating), a novel flavin adenine dinucleotide-dependent monooxygenase from *Candida parapsilosis* CBS604. J Bacteriol. 1997;179:6680–7. doi: 10.1128/jb.179.21.6680-6687.1997 9352916 PMC179595

[45] Kotyk A, Lapathitis G, Křenková Š. Glucose- and K(+)-induced acidification in different yeast species. Folia Microbiol. 1999;44:295–8. doi: 10.1007/BF02818550 10664885

[46] Martin R, Pohlers S, Mühlschlegel FA, Kurzai O. CO_2_ sensing in fungi: at the heart of metabolic signaling. Curr Genet. 2017;63:965–72. doi: 10.1007/s00294-017-0700-0 28493119

[47] Váchová L, Čáp M, Palková Z. Yeast colonies: a model for studies of aging, environmental adaptation, and longevity. Oxid Med Cell Longev. 2012;2012:601836. doi: 10.1155/2012/601836 22928081 PMC3425895

[48] MacPherson S, Larochelle M, Turcotte B. A fungal family of transcriptional regulators: the zinc cluster proteins. Microbiol Mol Biol Rev. 2006;70:583–604. doi: 10.1128/MMBR.00015-06 16959962 PMC1594591

[49] Todd RB, Andrianopoulos A. Evolution of a fungal regulatory gene family: the Zn(II)2Cys6 binuclear cluster DNA binding motif. Fungal Genet Biol. 1997;21:388–405. doi: 10.1006/fgbi.1997.0993 9290251

[50] Baum JA, Geever R, Giles NH. Expression of *qa-1F* activator protein: identification of upstream binding sites in the *qa* gene cluster and localization of the DNA-binding domain. Mol Cell Biol. 1987;7:1256–66. doi: 10.1128/mcb.7.3.1256-1266.1987 2951591 PMC365200

[51] Lundin M, Nehlin JO, Ronne H. Importance of a flanking AT-rich region in target site recognition by the GC box-binding zinc finger protein MIG1. Mol Cell Biol. 1994;14:1979–85. doi: 10.1128/mcb.14.3.1979-1985.1994 8114729 PMC358557

[52] Zaragoza O, Rodríguez C, Gancedo C. Isolation of the *MIG1* gene from *Candida albicans* and effects of its disruption on catabolite repression. J Bacteriol. 2000;182:320–6. doi: 10.1128/JB.182.2.320-326.2000 10629176 PMC94279

[53] Murad AM, d’Enfert C, Gaillardin C, Tournu H, Tekaia F, Talibi D, et al. Transcript profiling in *Candida albicans* reveals new cellular functions for the transcriptional repressors *Ca*Tup1, *Ca*Mig1 and *Ca*Nrg1. Mol Microbiol. 2001;42:981–93. doi: 10.1046/j.1365-2958.2001.02713.x 11737641

[54] Lagree K, Woolford CA, Huang MY, May G, McManus CJ, Solis NV, et al. Roles of *Candida albicans* Mig1 and Mig2 in glucose repression, pathogenicity traits, and *SNF1* essentiality. PLoS Genet. 2020;16:e1008582. doi: 10.1371/journal.pgen.1008582 31961865 PMC6994163

[55] Chorostecki U, Molina M, Pryszcz LP, Gabaldón T. MetaPhOrs 2.0: integrative, phylogeny-based inference of orthology and paralogy across the tree of life. Nucleic Acids Res. 2020;48:W553–7. doi: 10.1093/nar/gkaa282 32343307 PMC7319458

[56] Huerta-Cepas J, Capella-Gutiérrez S, Pryszcz LP, Marcet-Houben M, Gabaldón T. PhylomeDB v4: zooming into the plurality of evolutionary histories of a genome. Nucleic Acids Res. 2014;42:D897–902. doi: 10.1093/nar/gkt1177 24275491 PMC3964985

[57] von der Haar T, Tuite MF. Regulated translational bypass of stop codons in yeast. Trends Microbiol. 2007;15:78–86. doi: 10.1016/j.tim.2006.12.002 17187982

[58] Cantalapiedra CP, Hernández-Plaza A, Letunic I, Bork P, Huerta-Cepas J. eggNOG-mapper v2: Functional annotation, orthology assignments, and domain prediction at the metagenomic scale. Mol Biol Evol. 2021;38:5825–9. doi: 10.1093/molbev/msab293 34597405 PMC8662613

[59] Price MN, Dehal PS, Arkin AP. FastTree 2 –approximately maximum-likelihood trees for large alignments. PLoS One 2010;5:e9490. doi: 10.1371/journal.pone.0009490 20224823 PMC2835736

[60] Nguyen LT, Schmidt HA, von Haeseler A, Minh BQ. IQ-TREE: a fast and effective stochastic algorithm for estimating maximum-likelihood phylogenies. Mol Biol Evol. 2015;32:268–74. doi: 10.1093/molbev/msu300 25371430 PMC4271533

[61] Naranjo-Ortíz MA, Brock M, Brunke S, Hube B, Marcet-Houben M, Gabaldón T. Widespread inter- and intra-domain horizontal gene transfer of d-amino acid metabolism enzymes in eukaryotes. Front Microbiol. 2016;7:2001. doi: 10.3389/fmicb.2016.02001 28066338 PMC5169069

[62] Holland LM, Schröder MS, Turner SA, Taff H, Andes D, Grózer Z, et al. Comparative phenotypic analysis of the major fungal pathogens *Candida parapsilosis* and *Candida albicans*. PLoS Pathog. 2014;10:e1004365. doi: 10.1371/journal.ppat.1004365 25233198 PMC4169492

[63] Ding C, Butler G. Development of a gene knockout system in *Candida parapsilosis* reveals a conserved role for BCR1 in biofilm formation. Eukaryot Cell. 2007;6:1310–19. doi: 10.1128/EC.00136-07 17586721 PMC1951126

[64] Nosek J, Adamíková L, Zemanová J, Tomáška Ľ, Zufferey R, Mamoun CB. Genetic manipulation of the pathogenic yeast *Candida parapsilosis*. Curr Genet. 2002;42:27–35. doi: 10.1007/s00294-002-0326-7 12420143

[65] Koren S, Walenz BP, Berlin K, Miller JR, Bergman NH, Phillippy AM. Canu: scalable and accurate long-read assembly via adaptive k-mer weighting and repeat separation. Genome Res. 2017;27:722–36. doi: 10.1101/gr.215087.116 28298431 PMC5411767

[66] Bankevich A, Nurk S, Antipov D, Gurevich AA, Dvorkin M, Kulikov AS, et al. SPAdes: a new genome assembly algorithm and its applications to single-cell sequencing. J Comput Biol. 2012;19:455–77. doi: 10.1089/cmb.2012.0021 22506599 PMC3342519

[67] Oxford Nanopore Technologies. Medaka. 2020. Available from: https://github.com/nanoporetech/medaka

[68] Walker BJ, Abeel T, Shea T, Priest M, Abouelliel A, Sakthikumar S, et al. Pilon: an integrated tool for comprehensive microbial variant detection and genome assembly improvement. PLoS ONE 2014;9:e112963. doi: 10.1371/journal.pone.0112963 25409509 PMC4237348

[69] Guida A, Lindstädt C, Maguire SL, Ding C, Higgins DG, Corton NJ, et al. Using RNA-seq to determine the transcriptional landscape and the hypoxic response of the pathogenic yeast *Candida parapsilosis*. BMC Genomics 2011;12:628. doi: 10.1186/1471-2164-12-628 22192698 PMC3287387

[70] Frith MC, Kawaguchi R. Split-alignment of genomes finds orthologies more accurately. Genome Biol. 2015;16:106. doi: 10.1186/s13059-015-0670-9 25994148 PMC4464727

[71] Kent WJ. BLAT – the BLAST-like alignment tool. Genome Res. 2002;12:656–64. doi: 10.1101/gr.229202 11932250 PMC187518

[72] Stanke M, Schöffmann O, Morgenstern B, Waack S. Gene prediction in eukaryotes with a generalized hidden Markov model that uses hints from external sources. BMC Bioinformatics 2006;7:62. doi: 10.1186/1471-2105-7-62 16469098 PMC1409804

[73] Collart MA, Oliviero S. Preparation of yeast RNA. Curr Protoc Mol Biol. 2001;Chapter 13: Unit 13.12. doi: 10.1002/0471142727.mb1312s23 18265096

[74] Bolger AM, Lohse M, Usadel B. Trimmomatic: A flexible trimmer for Illumina Sequence Data. Bioinformatics 2014;30:2114–20. doi: 10.1093/bioinformatics/btu170 24695404 PMC4103590

[75] Kim D, Paggi JM, Park C, Bennett C, Salzberg SL. Graph-based genome alignment and genotyping with HISAT2 and HISAT-genotype. Nat Biotechnol. 2019;37:907–15. doi: 10.1038/s41587-019-0201-4 31375807 PMC7605509

[76] Li H, Handsaker B, Wysoker A, Fennell T, Ruan J, Homer N, et al. The Sequence alignment/map (SAM) format and SAMtools. Bioinformatics 2009;25:2078–9. doi: 10.1093/bioinformatics/btp352 19505943 PMC2723002

[77] Love MI, Huber W, Anders S. Moderated estimation of fold change and dispersion for RNA-seq data with DESeq2. Genome Biol. 2014;15:550. doi: 10.1186/s13059-014-0550-8 25516281 PMC4302049

[78] Kolde R. Package ‘pheatmap’. 2019. Available from: https://CRAN.R-project.org/package=pheatmap

[79] Amos B, Aurrecoechea C, Barba M, Barreto A, Basenko EY, Bażant W, et al. VEuPathDB: the eukaryotic pathogen, vector and host bioinformatics resource center. Nucleic Acids Res. 2022;50:D898–D911. doi: 10.1093/nar/gkab929 34718728 PMC8728164

[80] Kanehisa M, Sato Y. KEGG Mapper for inferring cellular functions from protein sequences. Protein Sci. 2020;29:28–35. doi: 10.1002/pro.3711 31423653 PMC6933857

[81] Bradford MA. A rapid and sensitive method for the quantitation of microgram quantities of protein utilizing the principle of dye-binding. Anal Biochem. 1976;72: 248–54. doi: 10.1006/abio.1976.9999 942051

[82] Michalski A, Damoc E, Lange O, Denisov E, Nolting D, Müller M., et al. Ultra high resolution linear ion trap Orbitrap mass spectrometer (Orbitrap Elite) facilitates top down LC MS/MS and versatile peptide fragmentation modes. Mol Cell Proteomics 2012;11:O111.013698. doi: 10.1074/mcp.O111.013698 22159718 PMC3316736

[83] Cox J, Mann M. MaxQuant enables high peptide identification rates, individualized p.p.b.-range mass accuracies and proteome-wide protein quantification. Nat Biotechnol. 2008;26:1367–72. doi: 10.1038/nbt.1511 19029910

[84] Tyanova S, Temu T, Sinitcyn P, Carlson A, Hein MY, Geiger T, et al. The Perseus computational platform for comprehensive analysis of (prote)omics data. Nat Methods 2016;13:731–40. doi: 10.1038/nmeth.3901 27348712

[85] Roggenkamp R, Sahm H, Wagner F. Microbial assimilation of methanol induction and function of catalase in *Candida boidinii*. FEBS Lett. 1974;4:283–6. doi: 10.1016/0014-5793(74)81230-5 4853207

[86] Noble SM, Johnson AD. Strains and strategies for large-scale gene deletion studies of the diploid human fungal pathogen *Candida albicans*. Eukaryot Cell 2005;4: 298–309. doi: 10.1128/EC.4.2.298-309.2005 15701792 PMC549318

[87] Kosa P, Gavenčiaková B, Nosek J. Development of a set of plasmid vectors for genetic manipulations of the pathogenic yeast *Candida parapsilosis*. Gene 2007;396:338–45. doi: 10.1016/j.gene.2007.04.008 17512139 PMC1994580

[88] Gietz RD, Schiestl RH. Transforming yeast with DNA. Methods Mol Cell Biol. 1995;5:255–69.

[89] Schindelin J, Arganda-Carreras I, Frise E, Kaynig V, Longair M, Pietzsch T, et al. Fiji: an open-source platform for biological-image analysis. Nat Methods 2012;9:676–82. doi: 10.1038/nmeth.2019 22743772 PMC3855844

[90] Winkler H, Adam G, Mattes E, Schanz M, Hartig A, Ruis H. Co-ordinate control of synthesis of mitochondrial and non-mitochondrial hemoproteins: a binding site for the HAP1 (CYP1) protein in the UAS region of the yeast catalase T gene (*CTT1*). EMBO J. 1988;7:1799–804. doi: 10.1002/j.1460-2075.1988.tb03011.x 2844525 PMC457171

[91] Edgar RC. MUSCLE: a multiple sequence alignment method with reduced time and space complexity. BMC Bioinformatics 2004;5:113. doi: 10.1186/1471-2105-5-113 15318951 PMC517706

[92] Hoang DT, Chernomor O, von Haeseler A, Minh BQ, Vinh LS. UFBoot2: Improving the ultrafast bootstrap approximation. Mol Biol Evol. 2018;35:518–22. doi: 10.1093/molbev/msx281 29077904 PMC5850222

[93] Gabaldón T. Large-scale assignment of orthology: back to phylogenetics? Genome Biol. 2008;9: 235. doi: 10.1186/gb-2008-9-10-235 18983710 PMC2760865

[94] Huerta-Cepas J, Gabaldón T. Assigning duplication events to relative temporal scales in genome-wide studies. Bioinformatics 2011;27:38–45. doi: 10.1093/bioinformatics/btq609 21075746

[95] Katoh K, Misawa K, Kuma K, Miyata T. MAFFT: a novel method for rapid multiple sequence alignment based on fast Fourier transform. Nucleic Acids Res. 2002;30:3059–66. doi: 10.1093/nar/gkf436 12136088 PMC135756

[96] Capella-Gutiérrez S, Silla-Martínez JM, Gabaldón T. trimAl: a tool for automated alignment trimming in large-scale phylogenetic analyses. Bioinformatics. 2009;25:1972–3. doi: 10.1093/bioinformatics/btp348 19505945 PMC2712344

[97] Kalyaanamoorthy S, Minh BQ, Wong TKF, von Haeseler A, Jermiin LS. ModelFinder: fast model selection for accurate phylogenetic estimates. Nat Methods. 2017;14:587–9. doi: 10.1038/nmeth.4285 28481363 PMC5453245

[98] Kanehisa M, Sato Y, Morishima K. BlastKOALA and GhostKOALA: KEGG tools for functional characterization of genome and metagenome sequences. J Mol Biol. 2016;428:726–31. doi: 10.1016/j.jmb.2015.11.006 26585406

[99] Mixão V, Hansen AP, Saus E, Boekhout T, Lass-Florl C, Gabaldón T. Whole-genome sequencing of the opportunistic yeast pathogen *Candida inconspicua* uncovers its hybrid origin. Front Genet. 2019;10:383. doi: 10.3389/fgene.2019.00383 31105748 PMC6494940

[100] Letunic I, Bork P. 20 years of the SMART protein domain annotation resource. Nucleic Acids Res. 2018;46:D493–6. doi: 10.1093/nar/gkx922 29040681 PMC5753352

[101] Lin JR, Hu J. SeqNLS: nuclear localization signal prediction based on frequent pattern mining and linear motif scoring. PLoS ONE 2013;8:e76864. doi: 10.1371/journal.pone.0076864 24204689 PMC3812174

