# Supplementary figures and images for: Transcriptome and proteome profiling reveals complex adaptations of Candida parapsilosis cells assimilating hydroxyaromatic carbon sources
Source: PLoS Genet. 2022 Mar 7;18(3):e1009815. doi: 10.1371/journal.pgen.1009815 (PMC8929692; doi:10.1371/journal.pgen.1009815)

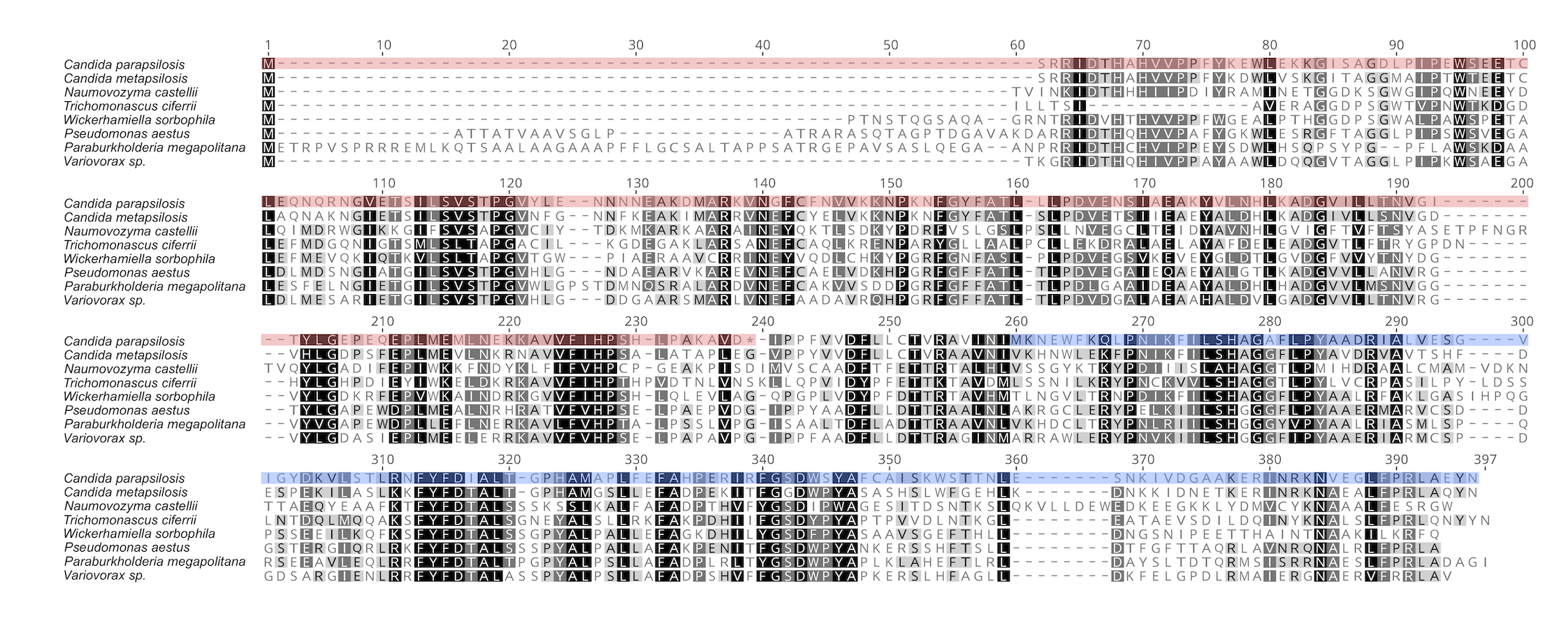

Supplement: S1 Fig — Amino acid sequence alignment of conceptual translation of C. parapsilosis CANPARB_p44920-A (red shading), short intergenic spacer, and CANPARB_p44910-A (blue shading) with yeast (C. metapsilosis (g2237), T. ciferrii (KAA8915622.1), W. sorbophila (XP_024665283.1), N. castellii (XP_003673849.1)) and bacterial (Pseudomonas aestus (P308_18355), Paraburkholderia megapolitana (SAMN05192543_101920), and Variovorax sp. (VAR608DRAFT_1163)) homologs. The alignment was calculated using MAFFT [95]. (TIF) [file pgen.1009815.s011.tif]

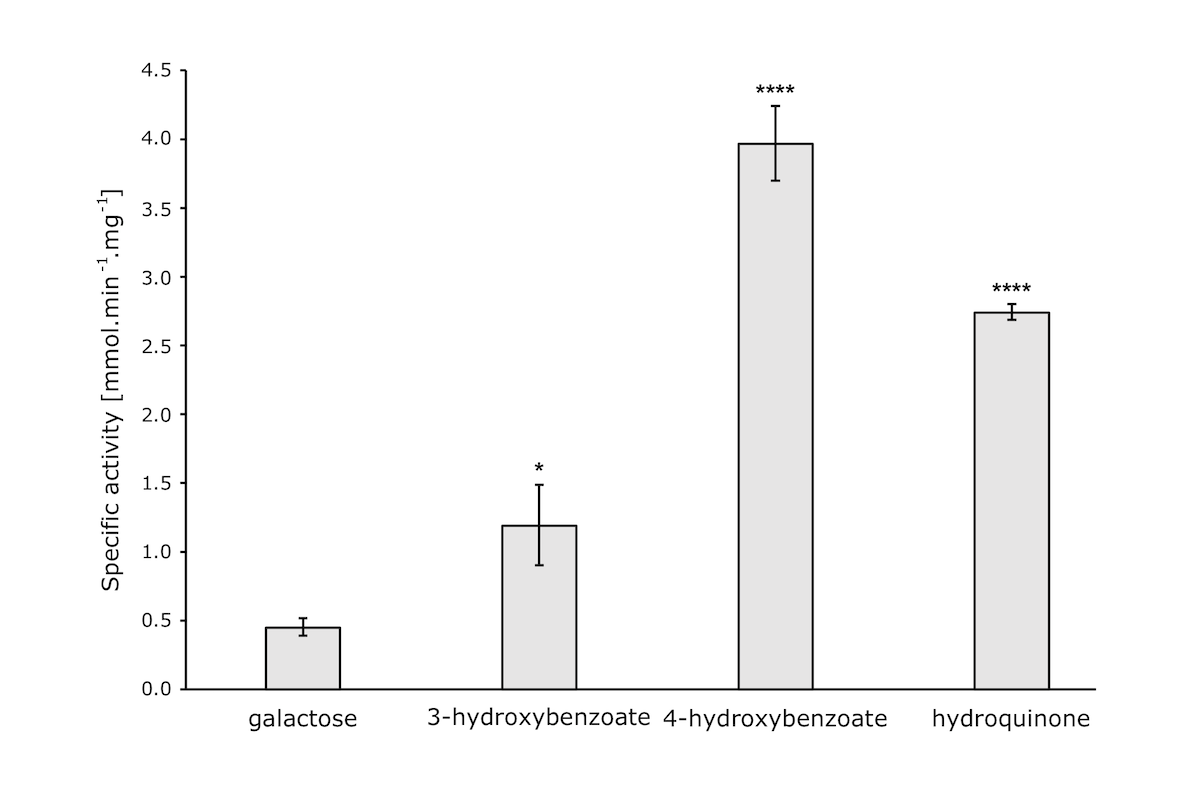

Supplement: S3 Fig — C. parapsilosis CLIB214 cells were grown in synthetic minimal media containing galactose, 3-hydroxybenzoate, 4-hydroxybenzoate or hydroquinone as a carbon source. Catalase activity was measured spectrophotometrically in cell lysates (see Materials and methods for details). The assays were performed in three independent experiments with three parallel measurements in each case, the bar graph shows the mean value ± standard deviation (S5 Table). The significance of differences between the samples (3-hydroxybenzoate, 4-hydroxybenzoate, hydroquinone) and the control (galactose) was evaluated by Student’s t-test (* P < 0.05; **** P < 0.0001). Note that the C. parapsilosis genome encodes three catalase homologs (i.e. CANPARB_p07760−A/CPAR2_207780, CANPARB_p28470-A/CPAR2_803840, and CANPARB_p28480−A/CPAR2_803850). (TIF) [file pgen.1009815.s013.tif]

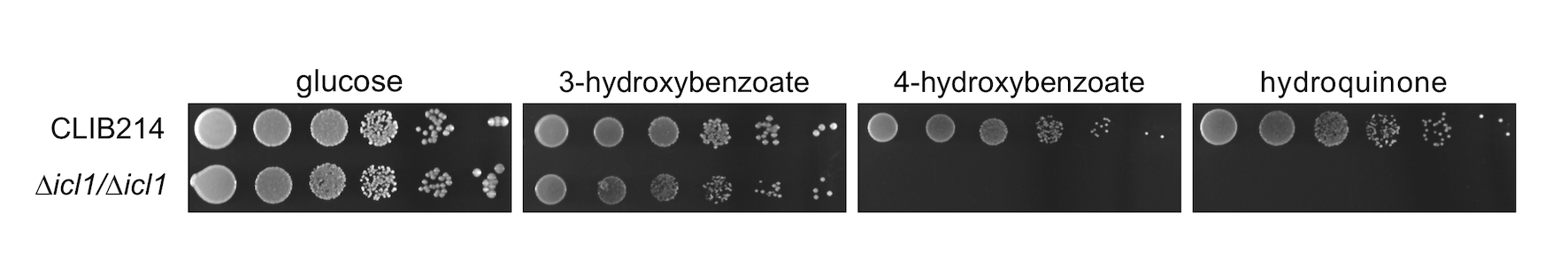

Supplement: S5 Fig — C. parapsilosis CLIB214 (wild type) and Δicl1/Δicl1 mutant cells were pre-grown overnight in a complex medium (YPD) at 28°C, washed with water and resuspended to ~ 6×10^6^ cells/ml. Serial fivefold dilutions were then spotted on solid synthetic media containing indicated carbon sources. The plates were incubated for 5 days at 28°C. (TIF) [file pgen.1009815.s015.tif]

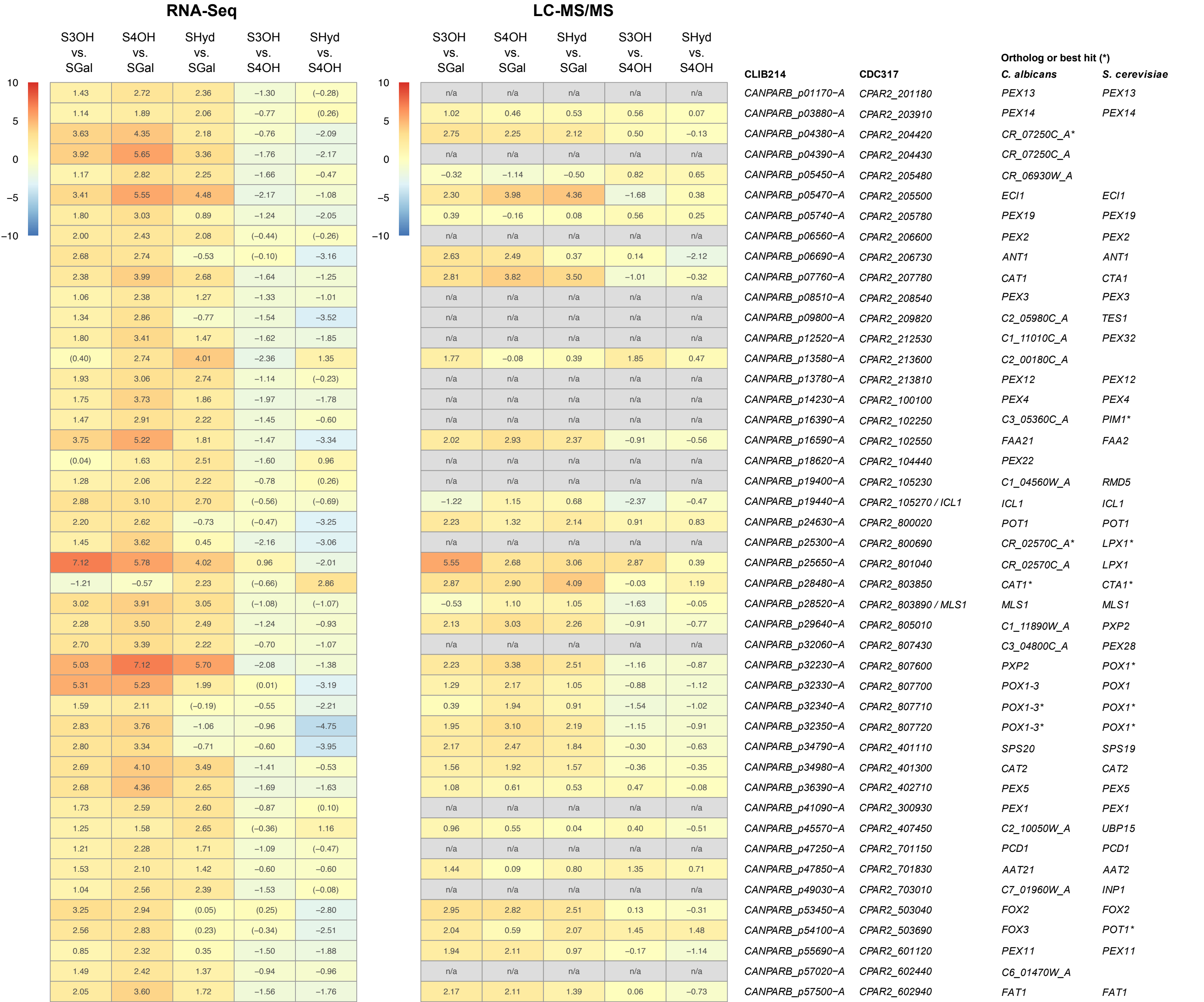

Supplement: S6 Fig — The heatmaps show the expression profiles obtained from the RNA-Seq and LC-MS/MS analyses. The log2 fold change values obtained by the RNA-Seq analysis (S1 Table) are shown on the left panel. Only the genes that are upregulated (log2 fold change ≥ 2; adjusted p-value ≤ 0.05) on at least one hydroxyaromatic substrate and code for protein products classified into categories ‘peroxisome’, ‘peroxisomal matrix’, ‘peroxisomal membrane’ or ‘peroxisomal importomer complex’ (based on the GO enrichment analysis; S6 Table) are included. Note that the values that are not statistically significant (i.e. adjusted p-value > 0.05) are shown in parentheses. The values on the right panel represent log2 of mean LFQ intensity ratios taken from the LC-MS/MS analysis (S3 Table). Orthologs or best hits (indicated by an asterisk) from the C. parapsilosis reference strain CDC317, C. albicans, and S. cerevisiae are shown. (PDF) [file pgen.1009815.s016.pdf]

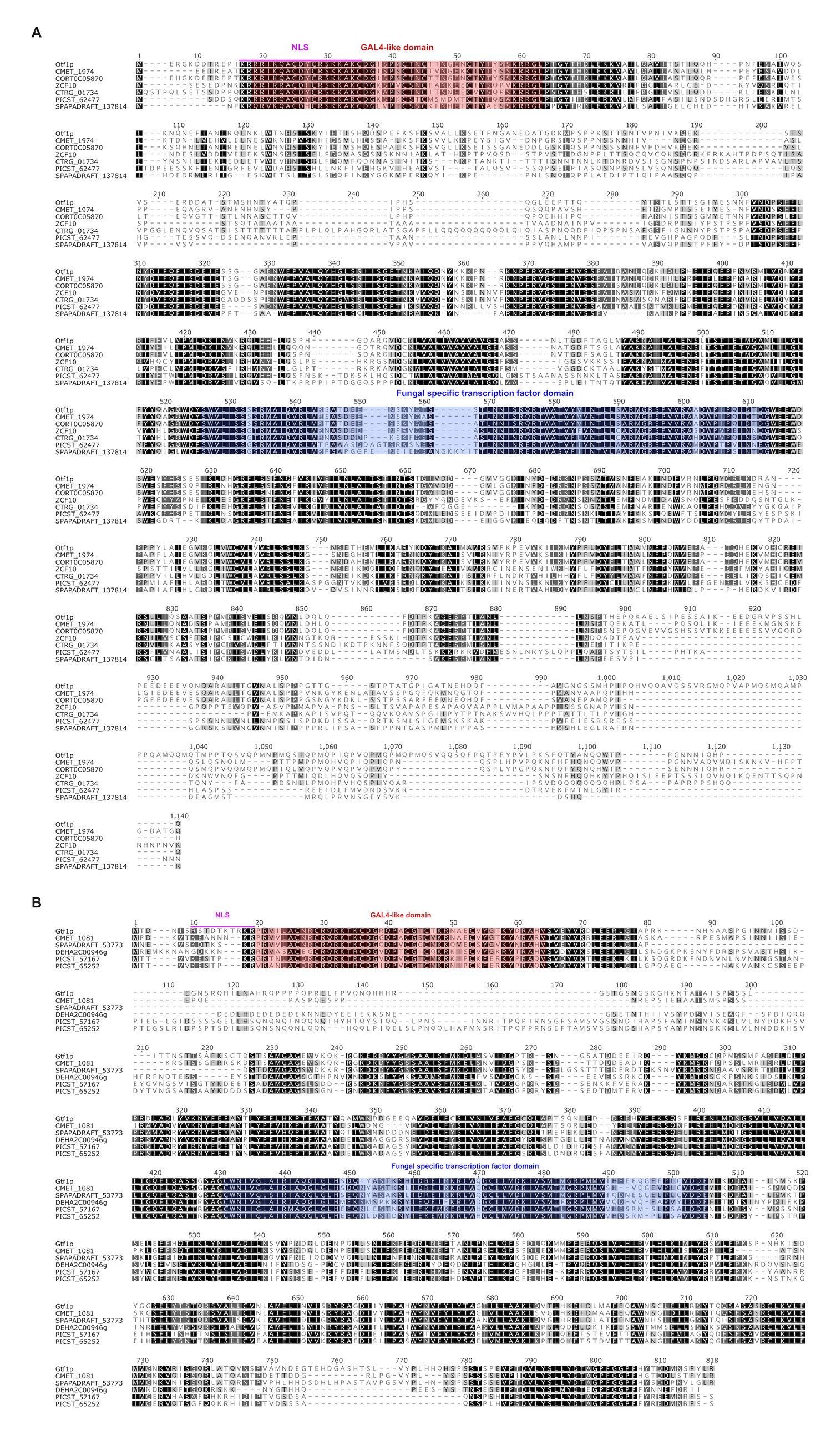

Supplement: S7 Fig — (A) Amino acid sequence alignment of C. parapsilosis Otf1p with the counterparts from C. metapsilosis (CMET_1974), C. orthopsilosis (CORT0C05870), C. albicans (ZCF10), C. tropicalis (CTRG_01734), Scheffersomyces stipitis (PICST_62477), and Spathaspora passalidarum (SPAPADRAFT_137814). (B) Amino acid sequence alignment of C. parapsilosis Gtf1p with the counterparts from C. metapsilosis (CMET_1081), S. passalidarum (SPAPADRAFT_53773), Debaryomyces hansenii (DEHA2C00946g), and S. stipitis (PICST_57167 and PICST_65252). The alignments were calculated using MAFFT [95]. The GAL4-like domain (red shading) and fungal specific transcription factor domain (blue shading) were predicted using SMART 8.0 [100]. Nuclear localisation signal (NLS, shown in magenta) was identified using SeqNLS [101]. (TIF) [file pgen.1009815.s017.tif]

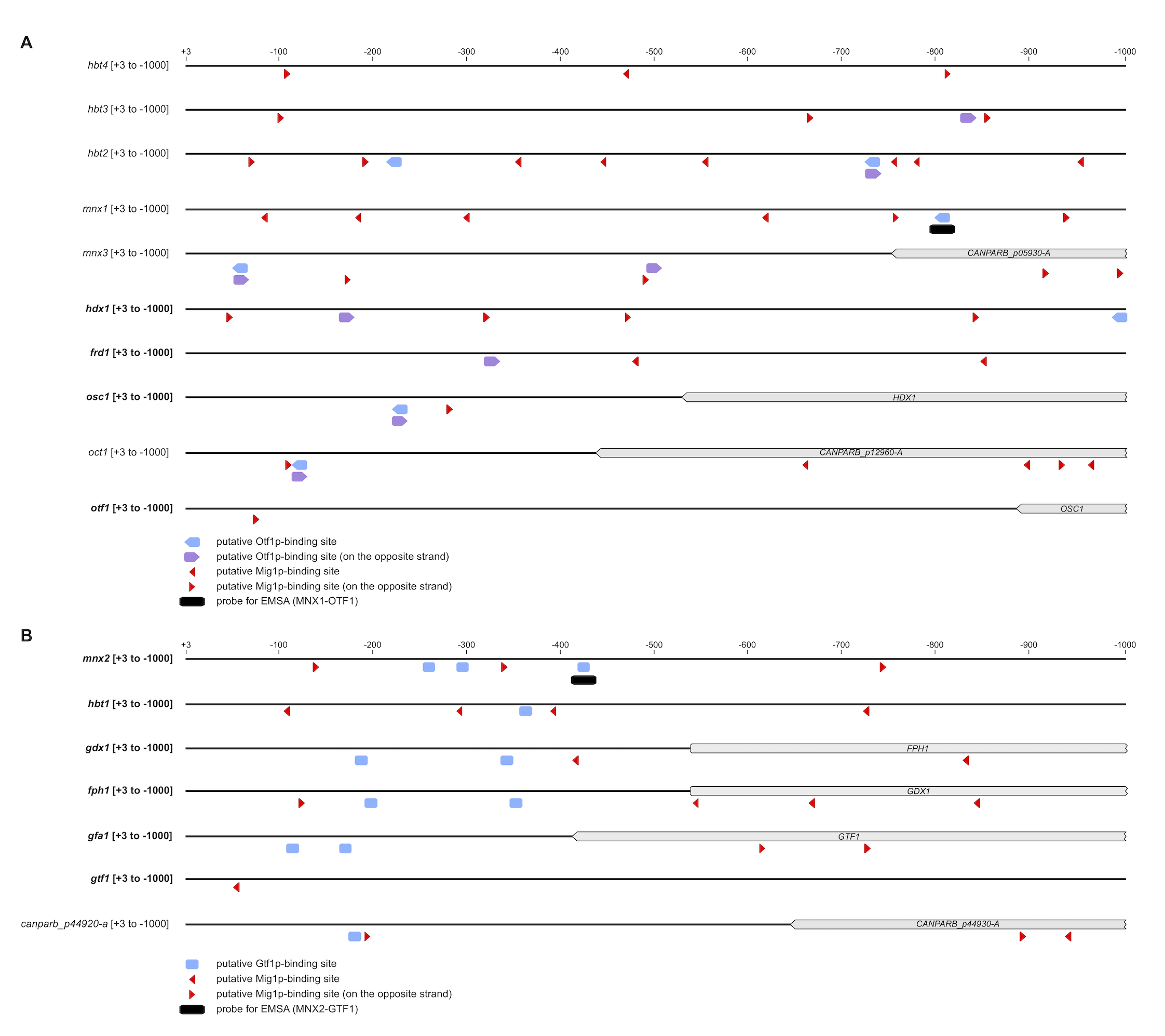

Supplement: S9 Fig — The occurrence of putative Otf1p (A) and Gtf1p (B) binding sites in the upstream regions (+3 to -1000) of the genes encoding the components of the 3-OAP and GP, respectively. The sequences arranged in the 3-OAP or GP gene clusters are indicated in bold. Putative Mig1p-binding sites and the positions of probes used in the EMSA experiments are also depicted. (TIF) [file pgen.1009815.s019.tif]

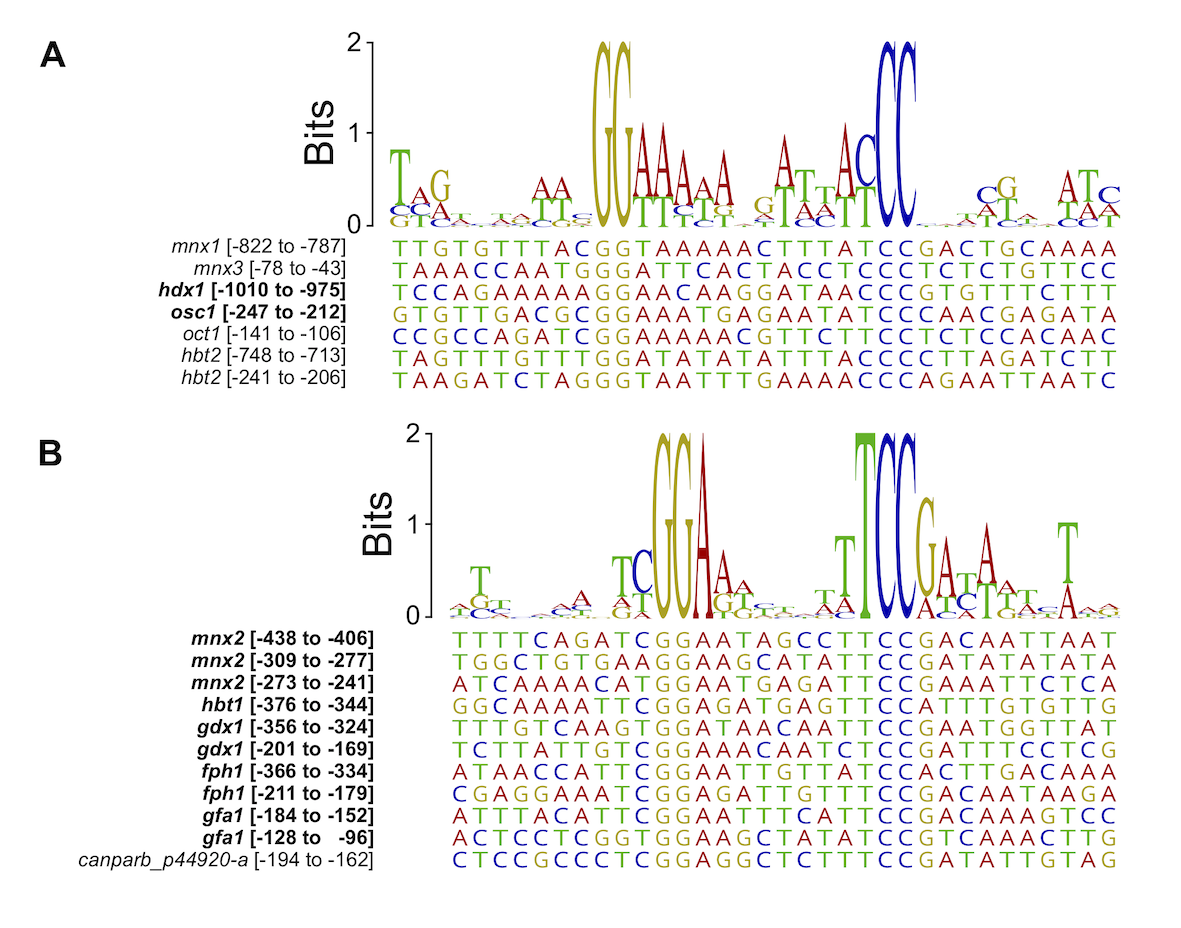

Supplement: S10 Fig — (A) Otf1p binding motif. (B) Gtf1p binding motif. The sequence logos were derived from predicted binding sites identified in the promoter sequences shown in S9 Fig. The sequences arranged in the 3-OAP or GP gene clusters are indicated in bold. Note that the Otf1p binding motif is asymmetrical and only the sites oriented toward the corresponding coding sequence (shown in blue in S9 Fig) were used in the alignment. (TIF) [file pgen.1009815.s020.tif]

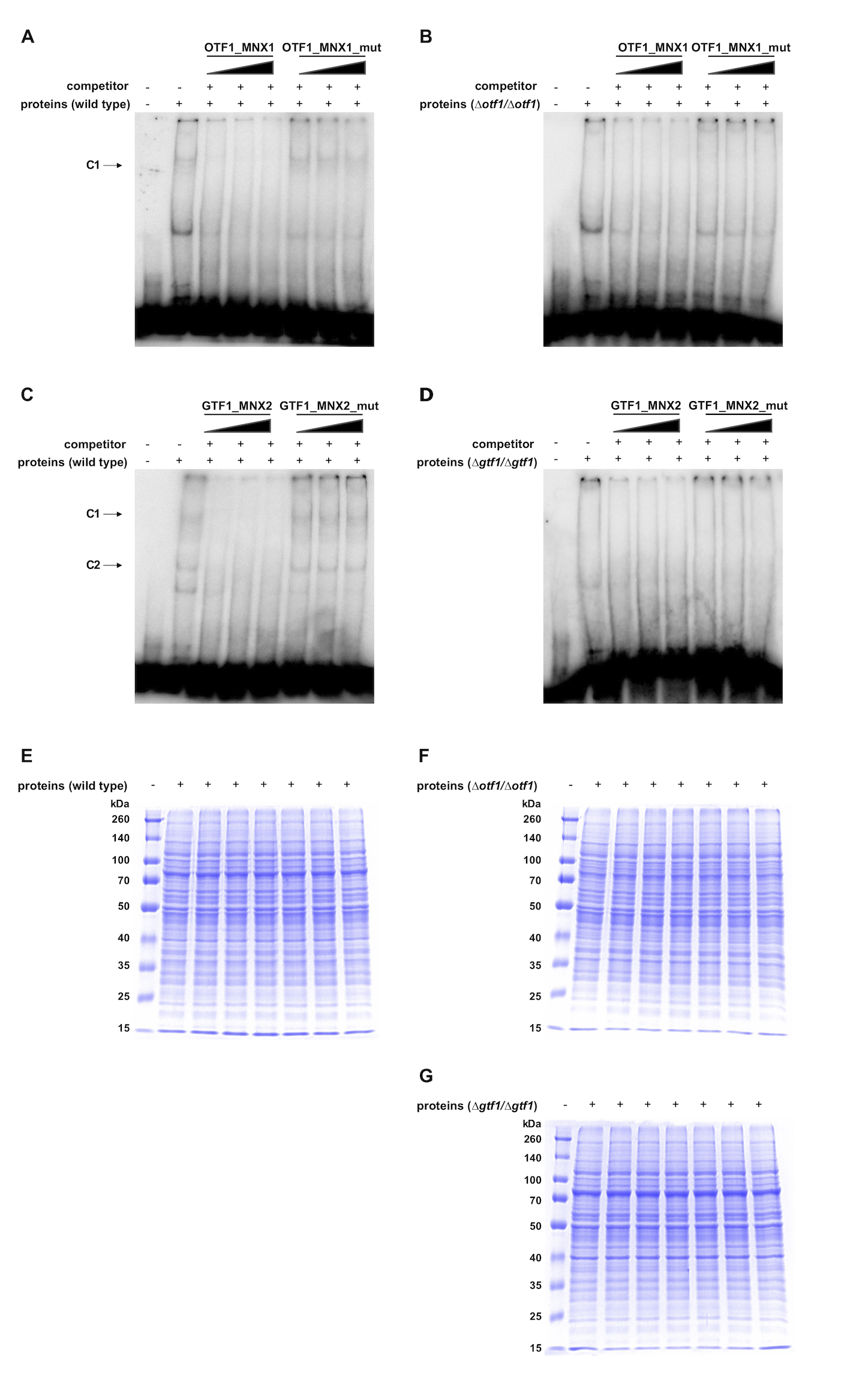

Supplement: S11 Fig — The EMSA experiments were performed using the protein extracts (i.e. ~ 15 μg) prepared from CPL2H1 (A),(C), Δotf1/Δotf1 (B), and Δgtf1/Δgtf1 (D) cells and the 5’ end-labeled dsDNA probes containing the predicted Otf1p-binding site from the MNX1 promoter (OTF1_MNX1; (A),(B)) or the Gtf1p-binding site from the MNX2 promoter (GTF1_MNX2; (C),(D)). The ds oligonucleotide competitors containing either the wild type (OTF1_MNX1, GTF1_MNX2) or mutated binding motifs (OTF1_MNX1_mut, GTF1_MNX2_mut) were used with increasing amounts of 100, 300, and 500 ng as indicated above lanes. The equivalent aliquots of protein extracts from CPL2H1 (E), Δotf1/Δotf1 (F), and Δgtf1/Δgtf1 (G) cells were also examined by SDS-PAGE and stained with PageBlue Protein Staining Solution (Thermo Scientific). Spectra Multicolor Broad Range Protein Ladder (Thermo Scientific; 10 μl) was used as a molecular weight standard. The gels were photographed using a GelDoc-It2 Imager (UVP) and the images were processed by VisionWorks Acquisition and Analysis Software (Analytik Jena). The gels are shown in Coomassie Blue pseudocolor. (TIF) [file pgen.1009815.s021.tif]

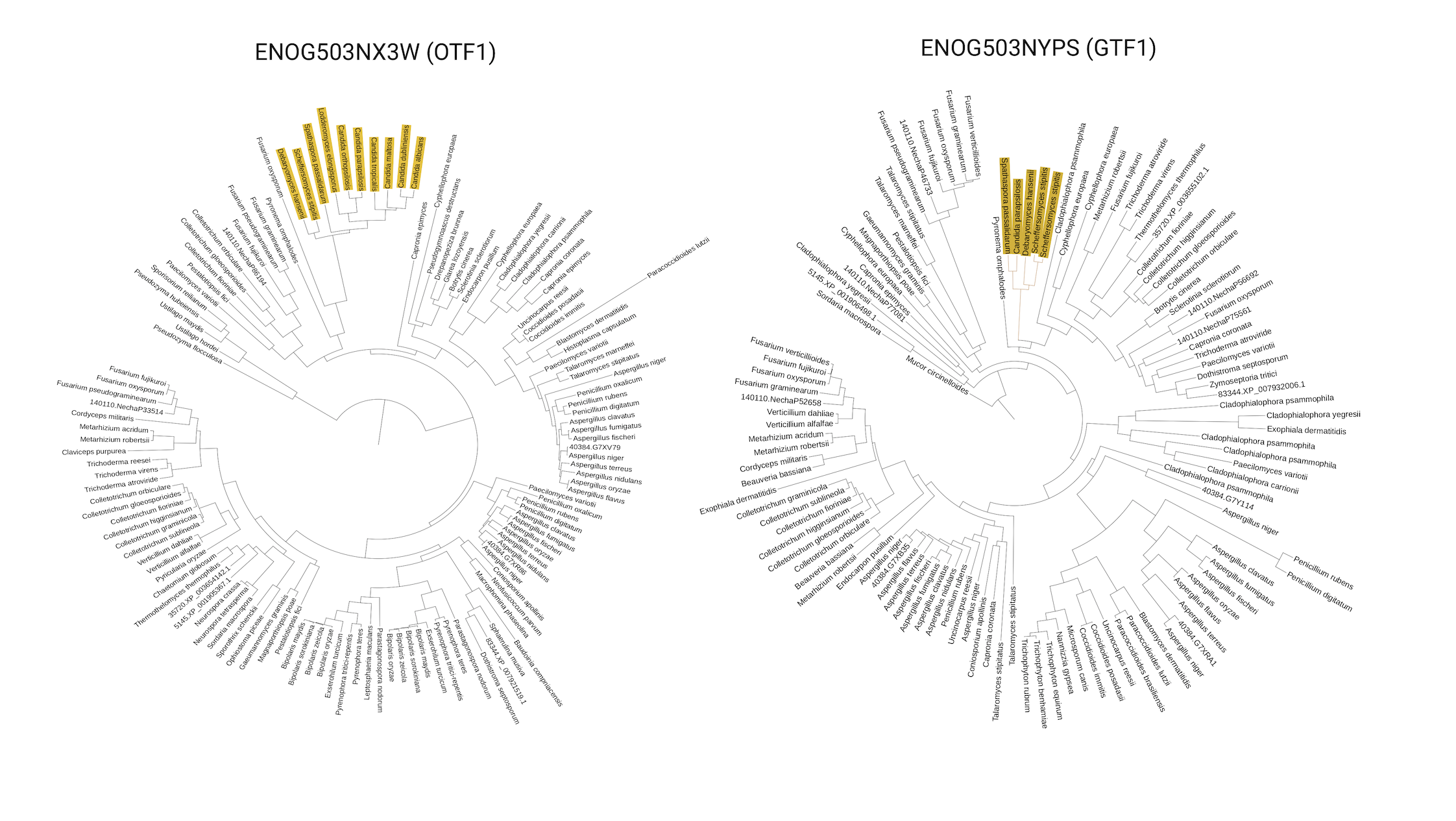

Supplement: S12 Fig — (TIF) [file pgen.1009815.s022.tif]

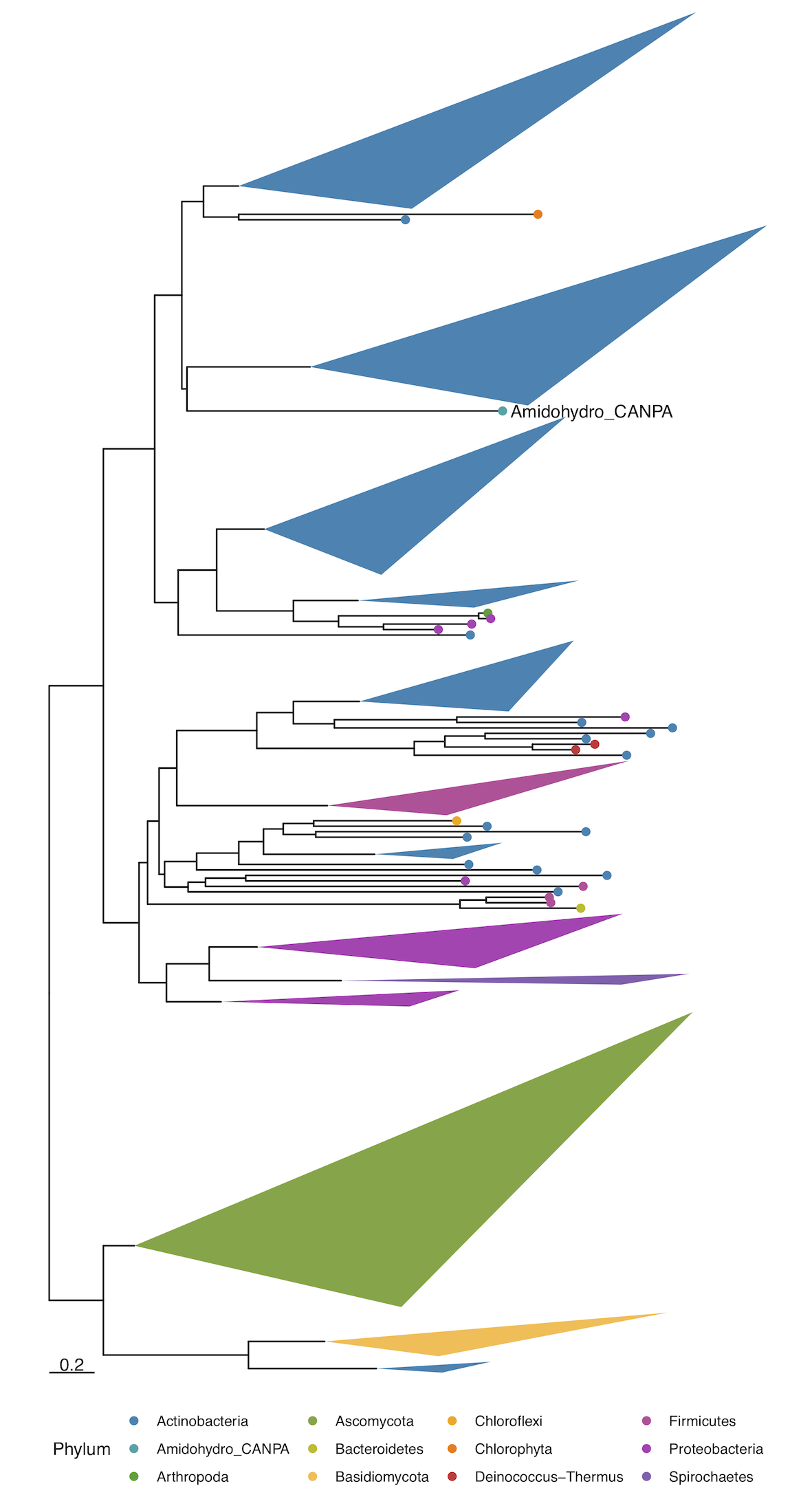

Supplement: S13 Fig — Maximum Likelihood phylogenetic tree of the subset of the 250 closest homologs to the C. parapsilosis amidohydrolase gene (marked as Amidohydro_CANPA). The tree was rooted at midpoint and monophyletic nodes representing species from the same phylum were collapsed and color coded. (TIF) [file pgen.1009815.s023.tif]

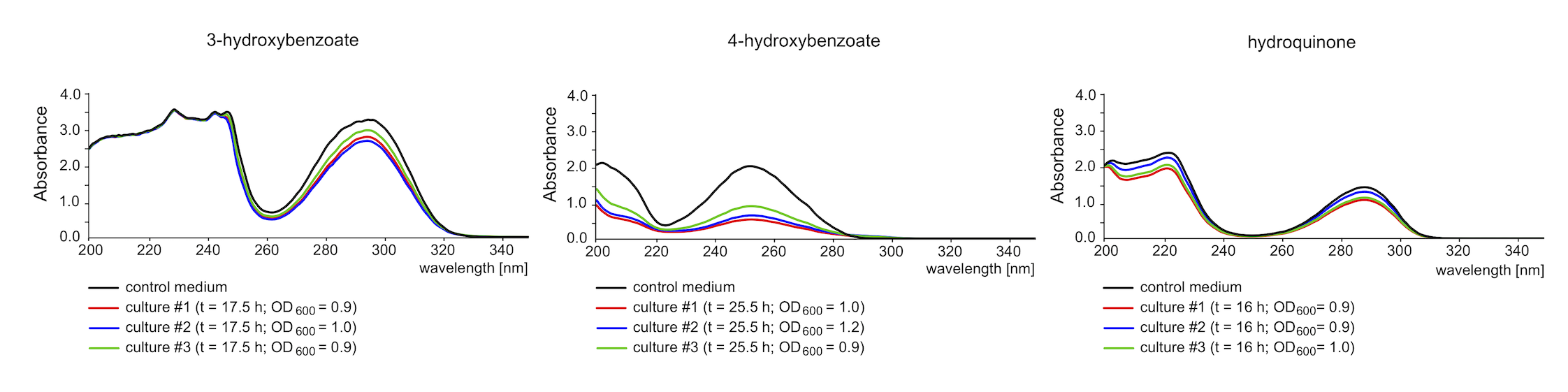

Supplement: S14 Fig — C. parapsilosis CLIB214 cells grown in the synthetic media containing a hydroxyaromatic substrate as a sole carbon source at 28°C till OD600 ~ 1. Substrate consumption was inferred from the absorption spectra (200–350 nm) measured in the media of three parallel cultures (shown in red, blue, and green) after cultivation (t = 17.5, 25.5, and 16 hours for 3-hydroxybenzoate, 4-hydroxybenzoate, and hydroquinone, respectively) as well as in the control medium. Each measurement was performed in three technical replicates. The samples were diluted 2-, 20-, and 5-fold prior analysis of 3-hydroxybenzoate, 4-hydroxybenzoate, and hydroquinone consumption, respectively. The dataset for each panel is shown in S7 Table. (TIF) [file pgen.1009815.s024.tif]
