# Supplementary material for: Transcriptome and proteome profiling reveals complex adaptations of Candida parapsilosis cells assimilating hydroxyaromatic carbon sources
Source: PLoS Genet. 2022 Mar 7;18(3):e1009815. doi: 10.1371/journal.pgen.1009815 (PMC8929692; doi:10.1371/journal.pgen.1009815)
Supplement: S2 Fig — The heatmaps show the expression profiles obtained by the RNA-Seq and LC-MS/MS analyses. The log2 fold change values obtained by the RNA-Seq analysis (S1 Table) are shown on the left panel. Only the genes that are upregulated (log2 fold change ≥ 2; adjusted p-value ≤ 0.05) on at least one hydroxyaromatic substrate and code for protein products classified as metabolic enzymes (based on the searches using the BlastKOALA (https://www.kegg.jp/blastkoala/; [98]) and KEGG Mapper tools (https://www.kegg.jp/kegg/tool/map_pathway.html; [80]) are included. Note that the values that are not statistically significant (i.e. adjusted p-value > 0.05) are shown in parentheses. The values on the right panel represent log2 of mean LFQ intensity ratios taken from the LC-MS/MS analysis (S3 Table). Note that the LFQ values imputed from a normal distribution were used for proteins that were not identified on all carbon sources (shown in parentheses). Proteins CANPARB_p24940-A and CANPARB_p24960-A, and similarly also CANPARB_p56420-A and CANPARB_p56500-A, have almost identical sequences and therefore could not be distinguished by the LC-MS/MS analysis. Orthologs or best hits (indicated by an asterisk) from the C. parapsilosis reference strain CDC317, C. albicans, and S. cerevisiae, and the KEGG IDs are indicated. (PDF) [file pgen.1009815.s012.pdf]

Colour scale (both panels): 10, 5, 0, −5, −10

RNA-Seq S3OH vs. SGal	RNA-Seq S4OH vs. SGal	RNA-Seq SHyd vs. SGal	RNA-Seq S3OH vs. S4OH	RNA-Seq SHyd vs. S4OH	LC-MS/MS S3OH vs. SGal	LC-MS/MS S4OH vs. SGal	LC-MS/MS SHyd vs. SGal	LC-MS/MS S3OH vs. S4OH	LC-MS/MS SHyd vs. S4OH	CLIB214	CDC317	Ortholog or best hit (*) C. albicans	Ortholog or best hit (*) S. cerevisiae	KEGG
3.11	1.42	1.19	1.65	(−0.14)	−0.91	−1.11	−0.59	0.20	0.51	CANPARB_p00120-A	CPAR2_200120	CPR6	CPR6	K05864
3.17	4.20	2.32	−1.06	−1.80	2.17	2.19	1.59	−0.02	−0.60	CANPARB_p01230-A	CPAR2_201240	CR_08920W_A		K00249
2.13	3.67	2.40	−1.55	−1.18	2.61	3.17	2.71	−0.55	−0.46	CANPARB_p01480-A	CPAR2_201500	CYB2	CYB2	K00101
1.84	2.36	(0.10)	(−0.52)	−2.16	n/a	n/a	n/a	n/a	n/a	CANPARB_p02080-A	CPAR2_202110	CR_04230W_A	NRK1	K10524
1.79	5.04	2.36	−3.13	−2.74	2.48	2.08	1.38	0.41	−0.70	CANPARB_p03410-A	CPAR2_203440	FDH1*	FDH1*	K00122
4.57	0.86	4.23	3.64	3.43	−1.41	−2.02	−0.76	0.61	1.26	CANPARB_p03420-A	CPAR2_203450	FDH1	FDH1	K00122
2.18	1.89	0.89	(0.28)	−0.92	n/a	n/a	n/a	n/a	n/a	CANPARB_p03590-A	CPAR2_203620	C1_11530C_A	JLP1	K19245
(0.04)	1.04	2.36	−1.01	1.41	n/a	n/a	n/a	n/a	n/a	CANPARB_p03700-A	CPAR2_203730	PTC1	PTC1	K19704
1.27	3.29	2.13	−2.02	−1.09	0.86	2.50	1.69	−1.64	−0.81	CANPARB_p03710-A	CPAR2_203740	C1_09980C_A	YJU3	K01054
1.18	3.97	3.48	−2.78	(−0.41)	2.39	2.11	2.34	0.28	0.23	CANPARB_p04580-A	CPAR2_204620	GST3		K00799
2.76	3.29	2.49	−0.55	−0.71	n/a	n/a	n/a	n/a	n/a	CANPARB_p05310-A	CPAR2_205350	CR_05750W_A		K21065
3.41	5.55	4.48	−2.17	−1.08	2.30	3.98	4.36	−1.68	0.38	CANPARB_p05470-A	CPAR2_205500	ECI1	ECI1	K13239
0.68	2.25	1.82	−1.58	−0.33	n/a	n/a	n/a	n/a	n/a	CANPARB_p05690-A	CPAR2_205730	CR_08550W_A	FBP26	K19029
0.48	8.03	7.30	−7.49	−0.73	2.14	8.88	8.48	−6.74	−0.39	CANPARB_p05940-A	CPAR2_205970 / MNX3	CR_07940W_A		K03380
0.73	(0.31)	2.17	(0.38)	1.95	n/a	n/a	n/a	n/a	n/a	CANPARB_p06800-A	CPAR2_206840	NAA20	NAT3	K17972
2.86	3.30	2.03	−0.46	−1.18	1.22	1.55	0.69	−0.32	−0.85	CANPARB_p07360-A	CPAR2_207380	C1_10310W_A	TMT1*	K22438
2.38	3.99	2.68	−1.64	−1.25	2.81	3.82	3.50	−1.01	−0.32	CANPARB_p07760-A	CPAR2_207780	CAT1	CTA1	K03781
(0.41)	3.06	1.57	−2.65	−1.39	n/a	n/a	n/a	n/a	n/a	CANPARB_p07820-A	CPAR2_207840	C1_11300C_A	PRP28	K12858
1.97	4.00	5.44	−1.99	1.50	n/a	n/a	n/a	n/a	n/a	CANPARB_p08830-A	CPAR2_208870	C1_07640C_A	RCK2*	K23785
(0.00)	2.17	0.99	−2.18	−1.08	n/a	n/a	n/a	n/a	n/a	CANPARB_p09580-A	CPAR2_209610	C2_04820W_A	MRM1	K15507
1.72	2.81	(−0.16)	−1.09	−2.86	n/a	n/a	n/a	n/a	n/a	CANPARB_p09810-A	CPAR2_209830	TES1	TES1*	K10805
1.62	2.23	0.82	−0.63	−1.31	n/a	n/a	n/a	n/a	n/a	CANPARB_p10390-A	CPAR2_210400	C2_01650W_A	IME4	K05925
5.68	4.78	−0.31	0.81	−4.99	0.65	0.86	0.18	−0.22	−0.68	CANPARB_p11020-A	CPAR2_211040	UGA1	UGA1	K13524
1.54	3.52	3.73	−1.99	(0.28)	1.13	0.99	1.00	0.15	0.01	CANPARB_p11110-A	CPAR2_211130	C2_04280W_A	RIM11*	K03083
(0.80)	2.10	1.80	−1.27	(−0.22)	n/a	n/a	n/a	n/a	n/a	CANPARB_p11150-A	CPAR2_211160	LIG4	DNL4	K10777
(1.07)	4.03	3.00	−2.76	(−0.87)	n/a	n/a	n/a	n/a	n/a	CANPARB_p11170-A	CPAR2_101810*	AYR1*	AYR1*	K06123
2.46	3.52	0.45	−1.09	−3.00	1.72	2.61	1.89	−0.89	−0.72	CANPARB_p12210-A	CPAR2_212210 / ALD5	ALD5	ALD5	K00128
1.58	7.28	5.75	−5.69	−1.52	1.80	10.31	9.72	−8.50	−0.59	CANPARB_p12970-A	CPAR2_212970 / OCT1	POT1-2	POT1*	K07513
(0.15)	1.14	2.43	−1.02	1.38	0.65	0.99	0.54	−0.34	−0.45	CANPARB_p13150-A	CPAR2_213150	C2_00630C_A	DAL2	K01477
(0.40)	2.74	4.01	−2.36	1.35	1.77	−0.08	0.39	1.85	0.47	CANPARB_p13580-A	CPAR2_213600	C2_00180C_A		K00365
3.98	6.30	5.28	−2.37	−1.39	n/a	n/a	n/a	n/a	n/a	CANPARB_p13910-A	CPAR2_213940 / CHA1	CHA1	CHA1*	K17989
1.54	2.99	3.76	−1.45	0.82	1.46	2.57	1.92	−1.11	−0.65	CANPARB_p14120-A	CPAR2_214150	C2_01540W_A		K23516
1.81	2.19	2.92	(−0.40)	0.82	n/a	n/a	n/a	n/a	n/a	CANPARB_p14130-A	CPAR2_214160	SMP2	PAH1	K15728
1.75	3.73	1.86	−1.97	−1.78	n/a	n/a	n/a	n/a	n/a	CANPARB_p14230-A	CPAR2_100100	PEX4	PEX4	K10689
3.10	4.34	2.07	−1.24	−2.19	1.23	0.93	0.56	0.30	−0.38	CANPARB_p14950-A	CPAR2_100820	CAR1	CAR1	K01476
(0.14)	2.40	2.98	−2.25	0.65	n/a	n/a	n/a	n/a	n/a	CANPARB_p15350-A	CPAR2_101230	PUT1	PUT1	K00318
0.55	0.80	2.06	(−0.28)	1.35	n/a	n/a	n/a	n/a	n/a	CANPARB_p16100-A	CPAR2_101960	MSS4	MSS4	K00889
1.47	2.91	2.22	−1.45	−0.60	n/a	n/a	n/a	n/a	n/a	CANPARB_p16390-A	CPAR2_102250	C3_05360C_A	PIM1*	K01338
3.76	5.66	3.74	−2.02	−2.14	5.76	4.12	1.90	1.64	−2.22	CANPARB_p16570-A	CPAR2_102570	FAA21*	FAA2*	K01897
6.19	7.47	5.25	−1.38	−2.22	5.14	4.85	5.23	0.29	0.38	CANPARB_p16580-A	CPAR2_102560	FAA21*	FAA2*	K01897
3.75	5.22	1.81	−1.47	−3.34	2.02	2.93	2.37	−0.91	−0.56	CANPARB_p16590-A	CPAR2_102550	FAA21	FAA2	K01897
4.27	8.67	5.54	−4.41	−3.15	6.10	10.18	8.50	−4.08	−1.68	CANPARB_p16940-A	CPAR2_102790 / MNX1			K00480
2.21	3.93	2.56	−1.76	−1.43	0.88	2.97	0.81	−2.09	−2.15	CANPARB_p17410-A	CPAR2_103260	CTN3	YAT2	K00624
4.28	5.03	2.52	−0.79	−2.44	3.62	1.75	1.79	1.87	0.04	CANPARB_p18000-A	CPAR2_103840	C2_04480W_A	ADH3*	K13953
2.54	3.83	1.75	−1.32	−2.00	1.30	0.92	0.85	0.38	−0.07	CANPARB_p18360-A	CPAR2_104190	GDH2	GDH2	K15371
2.12	3.20	0.63	−1.10	−2.47	2.55	3.00	2.56	−0.45	−0.44	CANPARB_p18610-A	CPAR2_104430	AMO2		K00276
(0.38)	1.28	2.41	−0.92	1.22	n/a	n/a	n/a	n/a	n/a	CANPARB_p18680-A	CPAR2_104500 / DBF2	DBF2	DBF2	K06684
0.84	2.22	2.23	−1.38	(0.10)	n/a	n/a	n/a	n/a	n/a	CANPARB_p19160-A	CPAR2_104990	C2_07430C_A	URH1	K01240
0.94	1.11	2.53	(−0.19)	1.50	n/a	n/a	n/a	n/a	n/a	CANPARB_p19200-A	CPAR2_105030	C2_07360W_A	TGS1	K14292
1.28	2.06	2.22	−0.78	(0.26)	n/a	n/a	n/a	n/a	n/a	CANPARB_p19400-A	CPAR2_105230	C1_04560W_A	RMD5	K23333
2.88	3.10	2.70	(−0.56)	(−0.69)	−1.22	1.15	0.68	−2.37	−0.47	CANPARB_p19440-A	CPAR2_105270 / ICL1	ICL1	ICL1	K01637
2.14	3.63	1.71	−1.51	−1.83	2.22	2.63	2.13	−0.40	−0.49	CANPARB_p20420-A	CPAR2_106260	C1_02270C_A	SPS19*	K13237
1.09	1.27	2.14	(−0.20)	0.97	0.18	0.52	0.08	−0.34	−0.44	CANPARB_p20470-A	CPAR2_106310	C1_02320C_A	YLR345W	K19030
−1.17	4.43	4.62	−5.53	(0.25)	n/a	n/a	n/a	n/a	n/a	CANPARB_p21520-A	CPAR2_107350	PRN1		K06911
0.73	4.55	5.02	−3.81	0.54	1.56	5.25	6.98	−3.70	1.73	CANPARB_p21750-A	CPAR2_107580	PRN4		K06911
0.73	1.75	2.07	−1.03	(0.40)	−0.12	−0.29	−0.38	0.18	−0.09	CANPARB_p22330-A	CPAR2_108180	C1_02040C_A	UBP16	K11874
2.41	3.95	2.95	−1.55	−0.92	1.00	1.44	1.23	−0.44	−0.21	CANPARB_p22830-A	CPAR2_108690	EHD3	EHD3	K05605
1.71	2.53	2.31	−0.84	(−0.13)	1.38	1.10	0.52	0.28	−0.58	CANPARB_p23030-A	CPAR2_108910	DAO1		K00273
1.00	0.62	2.27	(0.34)	1.72	n/a	n/a	n/a	n/a	n/a	CANPARB_p23640-A	CPAR2_109510	CAB3*	SIS2*	K01598
1.89	2.91	1.60	−1.21	−1.39	0.75	2.98	0.85	−2.23	−2.13	CANPARB_p24520-A	CPAR2_110400	CTN1	YAT1	K00624
3.12	(0.13)	(0.25)	2.91	(0.21)	0.05	−1.03	−0.62	1.08	0.40	CANPARB_p24550-A	CPAR2_110430	C5_03770C_A*	FDH1*	K00122
2.20	2.62	−0.73	(−0.47)	−3.25	2.23	1.32	2.14	0.91	0.83	CANPARB_p24630-A	CPAR2_800020	POT1	POT1	K07513
2.17	3.89	(−0.10)	−1.74	−3.90	n/a	n/a	n/a	n/a	n/a	CANPARB_p24660-A	CPAR2_800050	CHT1	CTS1*	K01183
0.79	2.26	2.76	−1.45	(0.66)	n/a	n/a	n/a	n/a	n/a	CANPARB_p24730-A	CPAR2_800120	C3_04350C_A*	AYR1*	K06123
4.27	6.79	4.17	−2.53	−2.58	(3.21)	(3.46)	(1.56)	−0.25	−1.90	CANPARB_p24940-A	CPAR2_800330	C3_04350C_A	AYR1*	K06123
1.53	4.42	3.00	−3.08	−1.57	n/a	n/a	n/a	n/a	n/a	CANPARB_p24950-A	CPAR2_800340	C3_04350C_A*	AYR1*	K06123
3.34	4.10	2.24	−0.96	−1.93	(3.21)	(3.46)	(1.56)	−0.25	−1.90	CANPARB_p24960-A	CPAR2_800350	C3_04350C_A*	AYR1*	K06123
2.14	2.29	1.44	(−0.18)	−0.76	0.17	2.14	2.41	−1.98	0.26	CANPARB_p24980-A	CPAR2_800370	CR_00350W_A	NMA1	K06210
3.36	4.74	3.69	−1.40	−0.99	2.10	3.26	3.11	−1.16	−0.15	CANPARB_p25860-A	CPAR2_801250	IDP2	IDP2	K00031
2.34	0.83	1.96	1.46	1.21	n/a	n/a	n/a	n/a	n/a	CANPARB_p25950-A	CPAR2_801340	UGT51C1	ATG26	K05841
2.00	2.67	0.49	−0.72	−2.07	−0.17	2.44	0.33	−2.60	−2.10	CANPARB_p26640-A	CPAR2_802010 / CIT1	CIT1	CIT1	K01647
(−0.01)	0.88	2.40	−0.91	1.60	n/a	n/a	n/a	n/a	n/a	CANPARB_p26670-A	CPAR2_802040	NOT4	MOT2	K10643
(−0.18)	1.55	2.12	−1.75	0.66	n/a	n/a	n/a	n/a	n/a	CANPARB_p27180-A	CPAR2_802550	RIB1	RIB1	K01497
2.31	2.90	1.23	−0.60	−1.58	1.98	0.07	1.07	1.91	1.00	CANPARB_p28140-A	CPAR2_803510	C1_09440W_A	PPA2	K01507
2.39	−2.64	−2.71	4.91	(0.07)	−0.12	−0.54	−0.37	0.42	0.17	CANPARB_p28470-A	CPAR2_803840 pseudogene	CAT1*	CTA1*	K03781
−1.21	−0.57	2.23	(−0.66)	2.86	2.87	2.90	4.09	−0.03	1.19	CANPARB_p28480-A	CPAR2_803850	CAT1*	CTA1*	K03781
3.02	3.91	3.05	(−1.08)	(−1.07)	−0.53	1.10	1.05	−1.63	−0.05	CANPARB_p28520-A	CPAR2_803890 / MLS1	MLS1	MLS1	K01638
3.40	−0.91	−1.07	4.23	(−0.05)	1.11	0.10	−0.06	1.01	−0.16	CANPARB_p29240-A	CPAR2_804600 / GAD1	GAD1	GAD1	K01580
2.08	4.07	1.41	−2.00	−2.55	4.43	5.08	4.39	−0.65	−0.69	CANPARB_p29390-A	CPAR2_804750	ADH2*	ADH3*	K13953
0.59	0.72	2.28	(−0.16)	1.64	n/a	n/a	n/a	n/a	n/a	CANPARB_p29570-A	CPAR2_804940	CR_06040W_A*	HAL5*	K08286
1.44	2.93	1.50	−1.50	−1.26	n/a	n/a	n/a	n/a	n/a	CANPARB_p30030-A	CPAR2_805400	FAA2-3*	FAA2*	K01897
2.42	3.48	1.92	−1.04	−1.33	n/a	n/a	n/a	n/a	n/a	CANPARB_p30040-A	CPAR2_805410	FAA2-3*	FAA2*	K01897
4.33	5.61	3.06	−1.35	−2.49	−0.65	3.56	2.44	−4.21	−1.11	CANPARB_p30050-A	CPAR2_805420	FAA2-3	FAA2*	K01897
2.97	1.87	0.39	1.05	−1.36	n/a	n/a	n/a	n/a	n/a	CANPARB_p30910-A	CPAR2_806280	PHO13	PHO13*	K01101
1.86	4.44	1.84	−2.57	−2.51	n/a	n/a	n/a	n/a	n/a	CANPARB_p31540-A	CPAR2_806910	GTT13	GTT1*	K00799
3.88	4.88	3.14	−1.32	−1.99	n/a	n/a	n/a	n/a	n/a	CANPARB_p31570-A	CPAR2_806940	GTT12	GTT1	K00799
3.09	5.01	3.71	−1.94	−1.26	5.91	4.53	3.34	1.38	−1.19	CANPARB_p31860-A	CPAR2_807230	FAA2	FAA2*	K01897
4.00	5.20	3.05	−1.25	−2.10	2.70	3.66	2.93	−0.96	−0.73	CANPARB_p31870-A	CPAR2_807240	FAA2*	FAA2*	K01897
5.03	7.12	5.70	−2.08	−1.38	2.23	3.38	2.51	−1.16	−0.87	CANPARB_p32230-A	CPAR2_807600	PXP2	POX1*	K00232
5.31	5.23	1.99	(0.01)	−3.19	1.29	2.17	1.05	−0.88	−1.12	CANPARB_p32330-A	CPAR2_807700	POX1-3	POX1	K00232
1.59	2.11	(−0.19)	−0.55	−2.21	0.39	1.94	0.91	−1.54	−1.02	CANPARB_p32340-A	CPAR2_807710	POX1-3*	POX1*	K00232
2.83	3.76	−1.06	−0.96	−4.75	1.95	3.10	2.19	−1.15	−0.91	CANPARB_p32350-A	CPAR2_807720	POX1-3*	POX1*	K00232
1.67	3.27	1.90	−1.60	−1.27	1.71	1.80	1.14	−0.10	−0.66	CANPARB_p33130-A	CPAR2_808510	C3_06730W_A	EHD3*	K12663
0.89	2.26	−0.64	−1.40	−2.79	0.90	1.41	3.23	−0.51	1.82	CANPARB_p33540-A	CPAR2_808920	PLB5*	PLB1*	K13333
0.84	2.10	(0.00)	−1.28	−2.00	n/a	n/a	n/a	n/a	n/a	CANPARB_p33720-A	CPAR2_400030	PTC8	PTC7*	K17508
0.95	1.42	3.17	(−0.49)	1.84	n/a	n/a	n/a	n/a	n/a	CANPARB_p34270-A	CPAR2_400570	C4_01300W_A	NEM1	K17617
0.92	2.10	1.24	−1.19	−0.76	n/a	n/a	n/a	n/a	n/a	CANPARB_p34770-A	CPAR2_401090	C4_00950C_A	TGL3	K14675
2.80	3.34	−0.71	−0.60	−3.95	2.17	2.47	1.84	−0.30	−0.63	CANPARB_p34790-A	CPAR2_401110	SPS20	SPS19	K13237
2.69	4.10	3.49	−1.41	−0.53	1.56	1.92	1.57	−0.36	−0.35	CANPARB_p34980-A	CPAR2_401300	CAT2	CAT2	K00624
3.64	2.46	2.41	1.14	(0.04)	2.19	2.56	1.96	−0.37	−0.61	CANPARB_p35170-A	CPAR2_401490	GST2*	URE2*	K00799
2.23	0.81	1.59	1.39	0.88	n/a	n/a	n/a	n/a	n/a	CANPARB_p35220-A	CPAR2_401540	C4_03050C_A	CPS1	K01293
1.19	1.28	3.08	(−0.12)	1.89	n/a	n/a	n/a	n/a	n/a	CANPARB_p35340-A	CPAR2_401650	HOS3	HOS3	K11484
2.87	4.30	2.55	−1.44	−1.68	0.86	1.43	0.98	−0.57	−0.45	CANPARB_p36380-A	CPAR2_402700	CAR2	CAR2	K00819
3.37	1.89	3.39	1.44	1.58	n/a	n/a	n/a	n/a	n/a	CANPARB_p36830-A	CPAR2_403150	C4_05250W_A		K09561
1.52	3.29	0.97	−1.79	−2.22	−1.83	0.22	−0.36	−2.05	−0.58	CANPARB_p37220-A	CPAR2_403550	ECM38		K00681
3.57	7.70	3.34	−4.16	−4.32	3.53	5.59	3.60	−2.06	−1.99	CANPARB_p37660-A	CPAR2_301880	C6_02660C_A	AMD2	K01426
0.72	2.39	3.22	−1.69	0.91	n/a	n/a	n/a	n/a	n/a	CANPARB_p38320-A	CPAR2_302540	C5_03150W_A	PRR1	K08286
(0.25)	1.73	2.62	−1.49	0.97	n/a	n/a	n/a	n/a	n/a	CANPARB_p39080-A	CPAR2_303300	HST3	HST3	K11121
0.82	2.02	2.73	−1.22	0.79	n/a	n/a	n/a	n/a	n/a	CANPARB_p40510-A	CPAR2_300350	C7_01150W_A		K15777
0.93	2.04	2.20	−1.12	(0.24)	n/a	n/a	n/a	n/a	n/a	CANPARB_p41650-A	CPAR2_301460	C7_00870W_A	GUD1	K01487
2.08	−0.74	−0.54	2.75	(0.30)	(−1.12)	(−1.32)	(−1.88)	(0.21)	(−0.56)	CANPARB_p41840-A	CPAR2_301660	C1_09520C_A	YMR209C	K19787
6.19	5.81	(0.12)	(0.34)	−5.62	1.88	3.92	0.81	−2.04	−3.11	CANPARB_p42170-A	CPAR2_404090	C3_03470W_A	UGA2	K00135
3.00	3.50	(0.36)	−0.53	−3.06	−0.38	1.23	0.53	−1.61	−0.70	CANPARB_p42180-A	CPAR2_404100	ALT1	ALT1	K00814
0.96	2.89	1.05	−1.92	−1.73	n/a	n/a	n/a	n/a	n/a	CANPARB_p43000-A	CPAR2_404920	C3_02060W_A		K01455
3.64	0.42	1.49	3.17	1.17	0.34	0.60	0.19	−0.26	−0.41	CANPARB_p43880-A	CPAR2_405780	PDC11*	PDC1*	K01568
1.74	2.60	2.33	−0.94	(−0.26)	n/a	n/a	n/a	n/a	n/a	CANPARB_p43900-A	CPAR2_405800	REV3	REV3	K02350
1.06	2.99	−0.87	−1.92	−3.70	1.46	2.67	1.69	−1.21	−0.98	CANPARB_p43910-A	CPAR2_405810	ACS1	ACS1	K01895
(−0.36)	5.54	5.10	−5.86	(−0.41)	2.81	8.84	8.28	−6.03	−0.56	CANPARB_p44540-A	CPAR2_406450 / OSC1	C2_07240C_A		K01027
1.93	2.01	1.37	(−0.11)	−0.54	1.69	2.02	1.61	−0.34	−0.41	CANPARB_p44640-A	CPAR2_406550	C2_07070W_A	ZTA1	K00344
−0.74	3.11	3.54	−3.83	0.51	−2.41	0.82	2.02	−3.24	1.19	CANPARB_p44900-A	CPAR2_406810	PST1	PST2*	K03809
6.45	(1.31)	(−0.13)	5.01	−1.38	n/a	n/a	n/a	n/a	n/a	CANPARB_p44920-A				K22213
(−0.16)	3.37	4.01	−3.53	0.72	−0.54	4.43	6.01	−4.98	1.58	CANPARB_p44930-A	CPAR2_406820	PST2	PST2*	K03809
−0.92	2.94	3.33	−3.84	0.47	−2.41	0.82	2.02	−3.24	1.19	CANPARB_p44940-A	CPAR2_406830	PST1*	PST2*	K03809
0.77	1.87	2.13	−1.12	(0.35)	n/a	n/a	n/a	n/a	n/a	CANPARB_p45100-A	CPAR2_406980	C2_08420W_A	MAG1	K01247
1.25	1.58	2.65	(−0.36)	1.16	0.96	0.55	0.04	0.40	−0.51	CANPARB_p45570-A	CPAR2_407450	C2_10050W_A	UBP15	K11838
(0.88)	3.88	3.53	−2.87	(−0.28)	(0.34)	(0.21)	(1.76)	(0.13)	1.55	CANPARB_p46120-A	CPAR2_700020	GCY1*	GCY1*	K18097
0.96	2.03	1.66	−1.08	(−0.27)	2.27	(0.09)	(0.14)	(2.18)	(0.04)	CANPARB_p46170-A	CPAR2_700070	NAG6	SEY1	K22698
(0.09)	1.24	2.01	−1.16	0.87	n/a	n/a	n/a	n/a	n/a	CANPARB_p46180-A	CPAR2_700080	HXK1	HXK1	K00844
1.76	2.11	2.94	(−0.38)	0.90	n/a	n/a	n/a	n/a	n/a	CANPARB_p46200-A	CPAR2_700100 / DAC1	DAC1		K01443
1.61	2.59	1.15	−1.00	−1.34	0.94	1.38	0.78	−0.44	−0.60	CANPARB_p46570-A	CPAR2_700470	C1_14320C_A	FMP41	K01557
4.83	1.94	2.72	2.83	0.86	2.99	1.02	1.14	1.97	0.13	CANPARB_p47230-A	CPAR2_701130	PLB3*	PLB3*	K13333
2.48	5.41	5.11	−2.92	(−0.27)	n/a	n/a	n/a	n/a	n/a	CANPARB_p47330-A	CPAR2_701230	CDG1		K00456
6.44	2.59	1.23	3.45	−1.39	7.53	5.51	4.37	2.02	−1.13	CANPARB_p47680-A	CPAR2_701660	FAA2-3*	FAA2*	K01897
1.53	2.10	1.42	−0.60	−0.60	1.44	0.09	0.80	1.35	0.71	CANPARB_p47850-A	CPAR2_701830	AAT21	AAT2	K14454
3.19	−0.45	−0.48	3.57	(0.07)	2.94	0.23	0.41	2.71	0.18	CANPARB_p48070-A	CPAR2_702060	EBP7	OYE3*	K00354
1.44	2.04	1.61	−0.62	(−0.33)	0.80	1.19	0.88	−0.39	−0.31	CANPARB_p48750-A	CPAR2_702750	AHP2	AHP1*	K14171
2.98	−0.87	−0.26	3.79	0.71	−0.83	−1.71	−1.14	0.88	0.57	CANPARB_p48970-A	CPAR2_806620*	C5_03770C_A*	FDH1*	K00122
2.87	3.44	1.39	−0.61	−1.97	3.07	2.64	1.75	0.44	−0.89	CANPARB_p49000-A	CPAR2_702980	C7_02010C_A	HFD1*	K00128
3.25	4.33	2.49	−1.11	−1.76	2.70	2.94	2.69	−0.24	−0.25	CANPARB_p49610-A	CPAR2_703580	C7_02920W_A	AMD2*	K01426
0.91	3.26	(0.31)	−2.25	−2.80	(0.42)	(2.63)	(−0.07)	(−2.21)	−2.69	CANPARB_p49620-A	CPAR2_703590	C7_02920W_A*	AMD2*	K01426
0.67	2.29	0.88	−1.62	−1.30	n/a	n/a	n/a	n/a	n/a	CANPARB_p49980-A	CPAR2_703950	IFE1	BDH2	K00004
9.11	0.67	−1.37	8.28	−1.92	5.06	0.34	2.56	4.72	2.22	CANPARB_p50340-A	CPAR2_704320 / MNX2			K00480
9.17	(0.22)	−1.85	8.83	−1.95	6.02	−0.29	2.53	6.31	2.82	CANPARB_p50360-A	CPAR2_704340 / GDX1			K00450
0.77	2.13	1.87	−1.36	(−0.18)	n/a	n/a	n/a	n/a	n/a	CANPARB_p50720-A	CPAR2_500330	SOD1*	SOD1*	K04565
3.35	3.01	0.88	(0.28)	−2.03	0.95	1.96	1.29	−1.01	−0.67	CANPARB_p51270-A	CPAR2_500880	ALD6	ALD4*	K00140
−0.46	0.56	2.10	−1.04	1.63	n/a	n/a	n/a	n/a	n/a	CANPARB_p51440-A	CPAR2_501050	TOP2	TOP2	K03164
(0.74)	4.16	1.78	−3.31	−2.29	n/a	n/a	n/a	n/a	n/a	CANPARB_p52170-A	CPAR2_501780	ADH2*	ADH2*	K13953
3.25	2.94	(0.05)	(0.25)	−2.80	2.95	2.82	2.51	0.13	−0.31	CANPARB_p53450-A	CPAR2_503040	FOX2	FOX2	K14729
0.94	2.40	1.15	−1.47	−1.15	n/a	n/a	n/a	n/a	n/a	CANPARB_p53460-A	CPAR2_503050	C3_00400C_A	TGL4	K14674
1.50	3.39	3.36	−1.88	(0.03)	0.66	0.84	1.14	−0.18	0.30	CANPARB_p53820-A	CPAR2_503420	DOT5	DOT5	K03564
2.56	2.83	(0.23)	(−0.34)	−2.51	2.04	0.59	2.07	1.45	1.48	CANPARB_p54100-A	CPAR2_503690	FOX3	POT1*	K07513
(0.51)	2.65	(−0.01)	−2.13	−2.54	n/a	n/a	n/a	n/a	n/a	CANPARB_p54370-A	CPAR2_503960 / TPT1	TPT1	TPT1	K10669
1.95	2.31	2.51	(−0.39)	(0.28)	n/a	n/a	n/a	n/a	n/a	CANPARB_p54520-A	CPAR2_504110	DAL1	DAL1	K01466
4.33	6.00	3.59	−1.88	−2.55	(4.30)	(4.03)	(3.43)	0.27	−0.60	CANPARB_p54790-A	CPAR2_600160	C6_03880W_A*	SPS19*	K00065
1.87	2.38	1.47	−0.54	−0.82	0.43	0.29	0.21	0.14	−0.08	CANPARB_p54820-A	CPAR2_600190	C6_03960W_A	YOL057W	K01277
3.48	6.60	5.68	−3.11	−0.86	4.05	3.68	3.09	0.37	−0.58	CANPARB_p54900-A	CPAR2_600270	AYR2	AYR1*	K06123
3.54	(0.44)	(0.03)	3.04	(−0.32)	n/a	n/a	n/a	n/a	n/a	CANPARB_p55320-A	CPAR2_600710	EBP7*	OYE3*	K00354
1.43	2.10	1.69	−0.69	−0.31	1.83	2.07	1.54	−0.24	−0.53	CANPARB_p55570-A	CPAR2_600960	C4_03050C_A*	CPS1*	K01293
5.01	4.66	2.66	(0.28)	−1.92	1.36	2.43	1.72	−1.06	−0.70	CANPARB_p55750-A	CPAR2_601180	HPD1		K23146
1.91	2.45	1.15	(−0.64)	−1.27	−0.63	0.55	0.52	−1.18	−0.03	CANPARB_p56420-A	CPAR2_601860* / GCV1*	GCV1*	GCV1*	K00605
1.97	2.54	1.36	−0.66	−1.15	−0.63	0.55	0.52	−1.18	−0.03	CANPARB_p56500-A	CPAR2_601860 / GCV1	GCV1	GCV1	K00605
0.63	2.62	2.32	−1.97	(−0.19)	n/a	n/a	n/a	n/a	n/a	CANPARB_p56620-A	CPAR2_602050	C6_02980C_A	CAB1	K09680
1.49	2.42	1.37	−0.94	−0.96	n/a	n/a	n/a	n/a	n/a	CANPARB_p57020-A	CPAR2_602440	C6_01470W_A		K23012
