# Supplementary material for: Transcriptome and proteome profiling reveals complex adaptations of Candida parapsilosis cells assimilating hydroxyaromatic carbon sources
Source: PLoS Genet. 2022 Mar 7;18(3):e1009815. doi: 10.1371/journal.pgen.1009815 (PMC8929692; doi:10.1371/journal.pgen.1009815)

A

CLIB214	CDC317	RNA-Seq S3OH vs. SGal	RNA-Seq S4OH vs. SGal	RNA-Seq SHyd vs. SGal	RNA-Seq S3OH vs. S4OH	RNA-Seq SHyd vs. S4OH	LC-MS/MS S3OH vs. SGal	LC-MS/MS S4OH vs. SGal	LC-MS/MS SHyd vs. SGal	LC-MS/MS S3OH vs. S4OH	LC-MS/MS SHyd vs. S4OH
CANPARB_p00620-A	*CPAR2_200640*	0.57	1.29	0.62	−0.75	(−0.58)	1.60	1.43	1.05	0.17	−0.38
CANPARB_p16570-A	*CPAR2_102570*	3.76	5.66	3.74	−2.02	−2.14	5.76	4.12	1.90	1.64	−2.22
CANPARB_p16580-A	*CPAR2_102560*	6.19	7.47	5.25	−1.38	−2.22	5.14	4.85	5.23	0.29	0.38
CANPARB_p16590-A	*CPAR2_102550*	3.75	5.22	1.81	−1.47	−3.34	2.02	2.93	2.37	−0.91	−0.56
CANPARB_p30020-A	*CPAR2_805390*	(0.07)	(0.19)	−0.45	(−0.15)	−0.54	n/a	n/a	n/a	n/a	n/a
CANPARB_p30030-A	*CPAR2_805400*	1.44	2.93	1.50	−1.50	−1.26	n/a	n/a	n/a	n/a	n/a
CANPARB_p30040-A	*CPAR2_805410*	2.42	3.48	1.92	−1.04	−1.33	n/a	n/a	n/a	n/a	n/a
CANPARB_p30050-A	*CPAR2_805420*	4.33	5.61	3.06	−1.35	−2.49	−0.65	3.56	2.44	−4.21	−1.11
CANPARB_p31860-A	*CPAR2_807230*	3.09	5.01	3.71	−1.94	−1.26	5.91	4.53	3.34	1.38	−1.19
CANPARB_p31870-A	*CPAR2_807240*	4.00	5.20	3.05	−1.25	−2.10	2.70	3.66	2.93	−0.96	−0.73
CANPARB_p47680-A	*CPAR2_701660*	6.44	2.59	1.23	3.45	−1.39	7.53	5.51	4.37	2.02	−1.13
CANPARB_p51210-A	*CPAR2_500820*	(−0.08)	(0.49)	−0.66	−0.60	−1.06	2.29	1.74	2.15	0.55	0.41

Color scale: 10, 5, 0, −5, −10

B

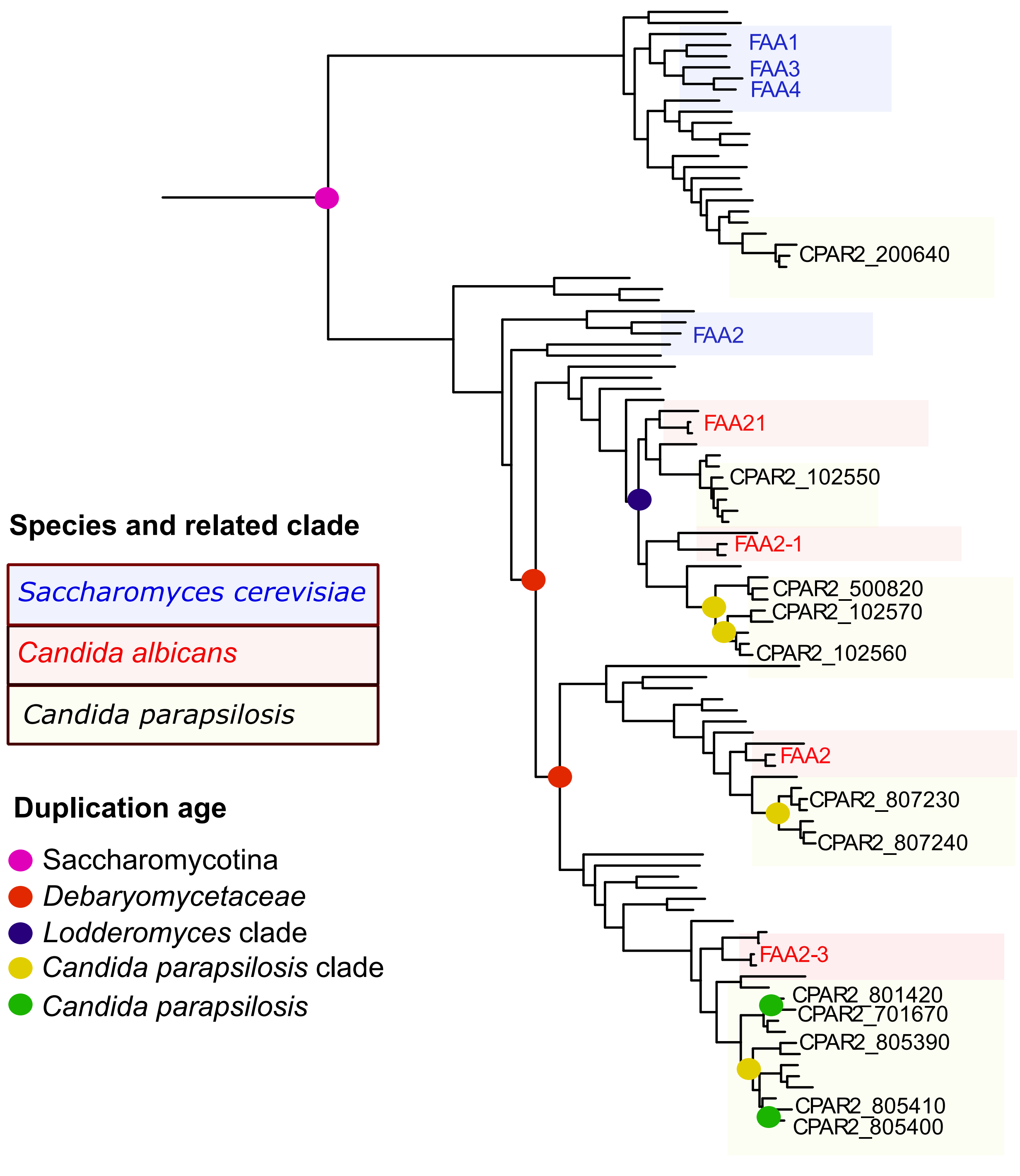

Supplement: S4 Fig — (A) The heatmaps show the expression profiles of C. parapsilosis FAA genes. The log2 fold change values obtained by the RNA-Seq analysis (S1 Table) are shown on the left panel. Note that the values that are not statistically significant (i.e. p > 0.05) are shown in parentheses. The values on the right panel represent log2 of mean LFQ intensity ratios taken from the LC-MS/MS analysis (S3 Table). (B) Phylogenetic relationships of C. parapsilosis FAA genes and their homologs in other yeasts. The CPAR2_200640 gene tree in phylome 498 from PhylomeDB (Candida inconspicua genome, described in [99]) was used as a template to create this figure, which is only shown partially here. Sequences from C. parapsilosis (black), C. albicans (red), and S. cerevisiae (blue) are highlighted with their names. Shadowed rectangles around them indicate, respectively, the spread of species from the C. parapsilosis sensu lato, C. albicans / C. dubliniensis / C. tropicalis clade, and Saccharomyces / Nakaseomyces clade. Colored circles indicate duplication nodes, with different colors indicating the relative age inferred from this duplication (see legend). (PDF) [file pgen.1009815.s014.pdf]
