# Supplementary material for: Transcriptome and proteome profiling reveals complex adaptations of Candida parapsilosis cells assimilating hydroxyaromatic carbon sources
Source: PLoS Genet. 2022 Mar 7;18(3):e1009815. doi: 10.1371/journal.pgen.1009815 (PMC8929692; doi:10.1371/journal.pgen.1009815)
Supplement: S8 Fig — The heatmap shows the genes downregulated (log2 fold change ≤ -2; adjusted p-value ≤ 0.05; S2 Table) in the mutants Δgtf1/Δgtf1 and Δotf1/Δotf1 compared to the parental strain CPL2H1 (Δgtf1/Δgtf1 vs. CPL2H1 and Δotf1/Δotf1 vs. CPL2H1). The cells were grown in an SMix15 medium containing three hydroxyaromatic carbon sources (i.e. 3-hydroxybenzoate, 4-hydroxybenzoate, and hydroquinone). Note that the values that are not statistically significant (i.e. adjusted p-value > 0.05) are shown in parentheses. Orthologs or best hits (indicated by an asterisk) from C. parapsilosis CDC317, C. albicans, and S. cerevisiae, and KEGG IDs are indicated. (PDF) [file pgen.1009815.s018.pdf]

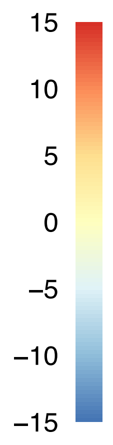

Δgtf1/Δgtf1 vs. CPL2H1	Δotf1/Δotf1 vs. CPL2H1	CLIB214	CDC317	Ortholog or best hit (*) C. albicans	S. cerevisiae	KEGG
−2.83	−0.43	*CANPARB_p00510−A*	*CPAR2_200520*	*FDH1**	*FDH1**	K00122
−2.15	(0.11)	*CANPARB_p00560−A*	*CPAR2_200580*	*AHA1*	*AHA1*	
(0.54)	−2.26	*CANPARB_p00790−A*	*CPAR2_200810*	*CR_09940W_A*	*SPS4*	
(1.56)	−2.06	*CANPARB_p00960−A*	*CPAR2_200970*	*CR_09390C_A*	*VBA1**	
−2.13	(−0.17)	*CANPARB_p01180−A*	*CPAR2_201190*	*CR_08880C_A*		
−2.46	−0.33	*CANPARB_p01350−A*	*CPAR2_201370*	*ASR2*		
−2.27	(−1.23)	*CANPARB_p02370−A*	*CPAR2_202400*	*CR_03940W_A*	*IPI1*	K14827
−3.17	(0.36)	*CANPARB_p02840−A*	*CPAR2_202870*			
−3.20	(0.24)	*CANPARB_p02850−A*	*CPAR2_202880*			
−2.26	(−0.17)	*CANPARB_p03420−A*	*CPAR2_203450*	*FDH1*	*FDH1*	K00122
−3.05	(−0.54)	*CANPARB_p03690−A*	*CPAR2_203720*	*CRP1*	*CCC2**	K17686
(0.26)	−3.56	*CANPARB_p03740−A*	*CPAR2_203770*			
−2.68	(0.18)	*CANPARB_p04100−A*	*CPAR2_204130*			
−3.52	0.74	*CANPARB_p05250−A*	*CPAR2_205290*	*RBR1*		
−2.73	(−0.14)	*CANPARB_p05870−A*	*CPAR2_205900*	*PGA13*		
−3.21	−0.48	*CANPARB_p06520−A*	*CPAR2_206560*	*WH11**	*HSP12**	
−4.07	−0.81	*CANPARB_p06530−A*	*CPAR2_206570*	*WH11*	*HSP12**	
−2.77	0.39	*CANPARB_p07280−A*	*CPAR2_207300*	*C1_10360C_A*	*YCR061W*	
(0.54)	−4.46	*CANPARB_p08450−A*	*CPAR2_208480*	*C1_07960W_A*	*LRP1*	K12592
−2.36	(−0.27)	*CANPARB_p08550−A*	*CPAR2_208580*	*OYE2**	*OYE3**	K00354
(−0.73)	−3.80	*CANPARB_p09230−A*	*CPAR2_209270*	*C2_04000C_A*		
(0.17)	−2.42	*CANPARB_p09560−A*	*CPAR2_209590*			
(0.00)	−2.47	*CANPARB_p10170−A*	*CPAR2_210190*			
−3.30	(0.06)	*CANPARB_p11020−A*	*CPAR2_211040*	*UGA1*	*UGA1*	K13524
−2.40	(−0.01)	*CANPARB_p11560−A*	*CPAR2_211560*	*HSP78*	*HSP78*	K03695
−2.01	(0.01)	*CANPARB_p11640−A*	*CPAR2_211640*	*C2_03110W_A*		
−3.15	(1.49)	*CANPARB_p12840−A*	*CPAR2_212850*	*HGT8*	*GAL2**	K08139
−2.94	−0.75	*CANPARB_p13360−A*	*CPAR2_213380*			
−3.74	0.52	*CANPARB_p14450−A*	*CPAR2_100330*	*C5_03080C_A*	*ESBP6*	
−5.21	(−0.59)	*CANPARB_p14690−A*	*CPAR2_100565*	*C5_02800C_A*		
−3.56	(0.05)	*CANPARB_p14700−A*	*CPAR2_100570*	*GAP1**	*GAP1**	K16261
−5.90	(−0.50)	*CANPARB_p14960−A*	*CPAR2_100830*	*C5_04480C_A*		
−2.38	(−0.09)	*CANPARB_p15020−A*	*CPAR2_100890*	*C5_04420W_A*	*AIM25*	
−2.47	−0.12	*CANPARB_p15800−A*	*CPAR2_101670*	*HSP12*	*HSP12**	
−0.39	−2.67	*CANPARB_p16940−A*	*CPAR2_102790 / MNX1*			K00480
−2.32	(−0.90)	*CANPARB_p17230−A*	*CPAR2_103080*	*GLX3*	*HSP31*	K22211
−2.23	(−0.09)	*CANPARB_p17510−A*	*CPAR2_103360*	*C3_03080W_A*		
−2.11	(0.07)	*CANPARB_p17800−A*	*CPAR2_103640*	*YVC1*	*YVC1*	
−2.23	−0.15	*CANPARB_p17850−A*	*CPAR2_103690*	*PGA45*		
−2.43	−0.71	*CANPARB_p18690−A*	*CPAR2_104510*			
−2.49	(−0.37)	*CANPARB_p19930−A*	*CPAR2_105760*	*ENA2*	*ENA2*	K01536
−3.30	(−0.14)	*CANPARB_p20260−A*	*CPAR2_106080*	*MIA40*	*MIA40*	K17782
−2.60	(0.15)	*CANPARB_p20330−A*	*CPAR2_106160 / GAL102*	*GAL102*	*GAL10**	
−2.06	0.41	*CANPARB_p20340−A*	*CPAR2_106170 / GAL7*	*GAL7*	*GAL7*	K00965
−3.00	(−0.51)	*CANPARB_p21360−A*	*CPAR2_107200*	*MFALPHA*		
−1.65	−3.93	*CANPARB_p21740−A*	*CPAR2_107570*	*C1_05830W_A*	*TMT1*	K22438
−3.59	0.51	*CANPARB_p22500−A*	*CPAR2_108360*	*HGT2**	*RGT2**	
−3.21	0.85	*CANPARB_p22510−A*	*CPAR2_108370*	*HGT1*	*HXT11**	
1.49	−2.90	*CANPARB_p22930−A*	*CPAR2_108810*	*C1_03440C_A*		
−2.07	(−0.01)	*CANPARB_p23400−A*	*CPAR2_109270*	*C1_01510W_B*		
−3.14	−0.23	*CANPARB_p24550−A*	*CPAR2_110430*	*C5_03770C_A**	*FDH1**	K00122
−3.91	−0.16	*CANPARB_p24790−A*	*CPAR2_800180*	*CR_02880W_A*		
−2.54	(−0.01)	*CANPARB_p25650−A*	*CPAR2_801040*	*CR_02570C_A*	*LPX1*	
−3.37	0.44	*CANPARB_p25910−A*	*CPAR2_801300 / PGA23*	*PGA23*		
−2.49	(0.73)	*CANPARB_p26280−A*	*CPAR2_801660*	*C1_12930C_A*	*CIN10**	
−2.32	(−0.42)	*CANPARB_p26700−A*	*CPAR2_802070*	*HXT5*	*ITR1**	
−3.11	(−0.01)	*CANPARB_p27610−A*	*CPAR2_802980*	*CSP37**		
−3.06	(−0.12)	*CANPARB_p27710−A*	*CPAR2_803090*	*CSP37*	*OM45*	
−2.02	−0.56	*CANPARB_p28220−A*	*CPAR2_803590*			
−3.77	−0.53	*CANPARB_p28470−A*	*CPAR2_803840*	*CAT1**	*CTA1**	K03781
−2.08	−0.21	*CANPARB_p29240−A*	*CPAR2_804600 / GAD1*	*GAD1*	*GAD1*	K01580
(−0.41)	−3.94	*CANPARB_p30970−A*	*CPAR2_806340*	*C2_09920W_A*	*RRG9*	
−2.09	−1.20	*CANPARB_p31570−A*	*CPAR2_806940*	*GTT12*	*GTT1*	K00799
−2.76	(−0.07)	*CANPARB_p32210−A*	*CPAR2_807580*	*C3_04690C_A*		
−2.89	(0.08)	*CANPARB_p32220−A*	*CPAR2_807590*	*C3_04730C_A*		
−2.17	0.44	*CANPARB_p32690−A*	*CPAR2_808070*	*C3_07760C_A*		K22369
−2.70	−0.67	*CANPARB_p32870−A*	*CPAR2_808250*	*C3_07590W_A*		
(1.41)	−2.84	*CANPARB_p32970−A*	*CPAR2_808350*	*C7_02260W_A*		
−4.95	−1.15	*CANPARB_p33250−A*	*CPAR2_808630*			
−2.57	−0.62	*CANPARB_p33540−A*	*CPAR2_808920*	*PLB5**	*PLB1**	K13333
−2.00	−0.47	*CANPARB_p35170−A*	*CPAR2_401490*	*GST2**	*URE2**	K00799
−3.07	(0.32)	*CANPARB_p35600−A*	*CPAR2_401900*	*C4_06220C_A*		
−2.11	(−0.64)	*CANPARB_p36410−A*	*CPAR2_402730*	*C4_00290C_A*		
−2.04	(0.09)	*CANPARB_p36550−A*	*CPAR2_402870*	*MEP2*	*MEP2*	K03320
−2.46	(−0.17)	*CANPARB_p36600−A*	*CPAR2_402920*	*RBT5*		
−2.17	−0.61	*CANPARB_p36830−A*	*CPAR2_403150*	*C4_05250W_A*		K09561
−2.12	(−0.05)	*CANPARB_p36840−A*	*CPAR2_403160*	*C1_11200W_A**		
−2.18	−2.98	*CANPARB_p37550−A*	*CPAR2_403880*			
−3.06	(−0.84)	*CANPARB_p37720−A*	*CPAR2_301940*	*C4_03160C_A*		
−2.40	0.42	*CANPARB_p38210−A*	*CPAR2_302430*	*C4_04810C_A*	*TRM8*	K03439
−2.45	−1.47	*CANPARB_p40820−A*	*CPAR2_300660*	*C7_00240W_A*	*YKL070W*	K07734
−2.68	(0.01)	*CANPARB_p40930−A*	*CPAR2_300770*	*TPO2*	*TPO1**	K08157
−2.19	(0.24)	*CANPARB_p41560−A*	*CPAR2_301390*	*HGT13*	*STL1**	
−2.23	−0.43	*CANPARB_p41790−A*	*CPAR2_301610*	*C7_00350C_A*		
−2.18	−0.42	*CANPARB_p44130−A*	*CPAR2_406040 / OYE32*	*OYE32*	*OYE3**	
−2.42	(−0.41)	*CANPARB_p44180−A*	*CPAR2_406090*	*C2_09600C_A**		
−2.48	−0.49	*CANPARB_p44300−A*	*CPAR2_406210*	*C2_07630C_A*	*RTC3*	
(0.36)	n/a	*CANPARB_p44550−A*	*CPAR2_406460 / OTF1*	*ZCF10*	*CAT8**	
−8.44	0.38	*CANPARB_p44910−A*				
−8.15	0.79	*CANPARB_p44920−A*				K22213
−2.45	(−0.09)	*CANPARB_p45020−A*	*CPAR2_406905*	*C2_08520C_A*		
−2.16	(0.25)	*CANPARB_p45090−A*	*CPAR2_406970*	*PGA46*		
−2.72	0.69	*CANPARB_p45400−A*	*CPAR2_407280*	*C2_09800C_A*		
−2.45	(−0.19)	*CANPARB_p45610−A*	*CPAR2_407490*	*C2_10120W_A*	*PLP2*	
−2.96	−0.50	*CANPARB_p47050−A*	*CPAR2_700950*	*AOX1*		
−4.55	−0.61	*CANPARB_p47680−A*	*CPAR2_701660*	*FAA2-3**	*FAA2**	K01897
−3.15	(−0.04)	*CANPARB_p48990−A*	*CPAR2_702970*	*C7_01990C_A*	*YIL166C**	
(−0.73)	−3.84	*CANPARB_p49120−A*				
−2.48	(−0.05)	*CANPARB_p50280−A*	*CPAR2_704260*	*C7_03030W_A*		
−14.45	−0.12	*CANPARB_p50340−A*	*CPAR2_704320 / MNX1*			K00480
−12.74	0.57	*CANPARB_p50350−A*	*CPAR2_704330 / HBT1*	*C3_03440C_A**	*TPO2**	
−6.52	0.17	*CANPARB_p50360−A*	*CPAR2_704340 / GDX1*			K00450
−7.12	−0.07	*CANPARB_p50370−A*	*CPAR2_704350 / FPH1*	*C2_07980W_A*	*FMP41**	
−7.56	−0.52	*CANPARB_p50380−A*	*CPAR2_704360 / GFA1*			
n/a	(0.16)	*CANPARB_p50390−A*	*CPAR2_704370 / GTF1*	*ZCF25**	*ASG1**	
−2.38	(−0.07)	*CANPARB_p51070−A*	*CPAR2_500680*	*C4_05850C_A*	*YGR111W*	
−3.04	−0.30	*CANPARB_p52710−A*	*CPAR2_502310*	*C5_00100C_A*		
(−1.88)	−2.27	*CANPARB_p52850−A*	*CPAR2_502450*	*MRV4*		
−2.52	(−0.73)	*CANPARB_p53130−A*	*CPAR2_502730*			
−2.26	−0.61	*CANPARB_p54360−A*	*CPAR2_503950*	*ALK2**	*DIT2**	
−2.26	−0.99	*CANPARB_p55210−A*	*CPAR2_600600*	*CR_06510W_A*		
−2.72	(−0.32)	*CANPARB_p55250−A*	*CPAR2_600640*			
−2.15	−0.60	*CANPARB_p55750−A*	*CPAR2_601180*	*HPD1*		K23146
−2.39	−0.41	*CANPARB_p56100−A*	*CPAR2_601530*	*C6_03240W_A*	*GRE2*	
−2.39	−0.49	*CANPARB_p56400−A*		*C6_02480W_A**	*ADH7**	K00002
−2.03	−0.48	*CANPARB_p56520−A*	*CPAR2_601840*	*C6_02480W_A*	*ADH7*	K00002
(−0.25)	−2.19	*CANPARB_p56960−A*	*CPAR2_602380*			
−2.50	−0.31	*CANPARB_p57520−A*	*CPAR2_602960*	*C6_00760W_A*	*FES1*	K09562
